# High-throughput analysis of hematopoietic stem cell engraftment after intravenous and intracerebroventricular dosing

**DOI:** 10.1016/j.ymthe.2022.05.022

**Published:** 2022-05-25

**Authors:** Robert N. Plasschaert, Mark P. DeAndrade, Fritz Hull, Quoc Nguyen, Tara Peterson, Aimin Yan, Mariana Loperfido, Cristina Baricordi, Luigi Barbarossa, John K. Yoon, Yildirim Dogan, Zeenath Unnisa, Jeffrey W. Schindler, Niek P. van Til, Luca Biasco, Chris Mason

**Affiliations:** 1 AVROBIO, Inc, Cambridge, MA 02139, USA; 2 Department of Child Neurology, Amsterdam Leukodystrophy Center, Emma Children’s Hospital, Amsterdam University Medical Centers, VU University, and Amsterdam Neuroscience, Amsterdam, the Netherlands; 3 Great Ormond Street Institute of Child Health, University College London, London, UK; 4 Advanced Centre for Biochemical Engineering, University College London, London, UK

**Keywords:** hematopoietic stem cells, microglia, autologous transplant, gene therapy, lentiviral vector, Parkinson’s disease, frontotemporal dementia, neurodegenerative disease, single cell analysis, hematopoiesis

## Abstract

Hematopoietic stem/progenitor cell gene therapy (HSPC-GT) has shown clear neurological benefit in rare diseases, which is achieved through the engraftment of genetically modified microglia-like cells (MLCs) in the brain. Still, the engraftment dynamics and the nature of engineered MLCs, as well as their potential use in common neurogenerative diseases, have remained largely unexplored. Here, we comprehensively characterized how different routes of administration affect the biodistribution of genetically engineered MLCs and other HSPC derivatives in mice. We generated a high-resolution single-cell transcriptional map of MLCs and discovered that they could clearly be distinguished from macrophages as well as from resident microglia by the expression of a specific gene signature that is reflective of their HSPC ontogeny and irrespective of their long-term engraftment history. Lastly, using murine models of Parkinson’s disease and frontotemporal dementia, we demonstrated that MLCs can deliver therapeutically relevant levels of transgenic protein to the brain, thereby opening avenues for the clinical translation of HSPC-GT to the treatment of major neurological diseases.

## Graphical abstract

**Figure:**
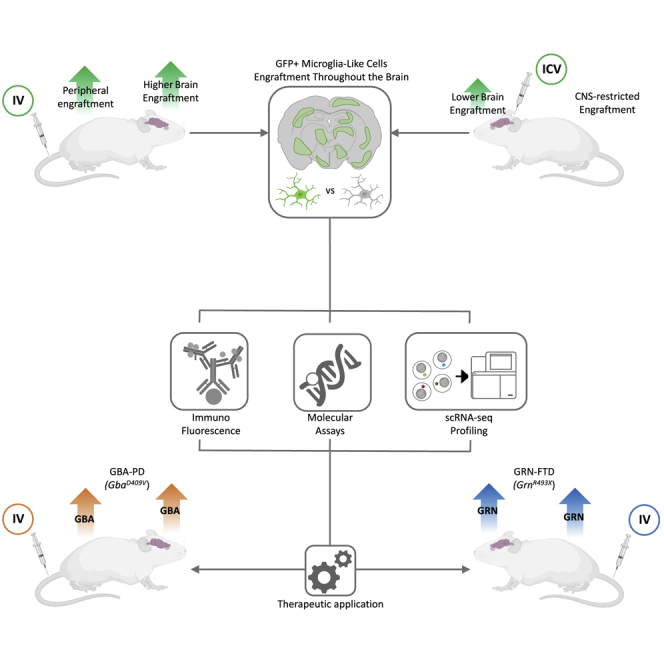

## Teaser

Administration of genetically modified hematopoietic stem cells has lasting clinical benefit for certain rare neurological diseases. This study characterizes engrafted cells in the brain after this process using two routes of administration in mice and shows the method’s ability to provide potentially therapeutic transgene in two models of neurodegenerative disease.

## Introduction

Hematopoietic stem/progenitor cell gene therapy (HSPC-GT) is a well-established paradigm for treating monogenic diseases, with more than 300 patients currently dosed.^1^, ^2^, ^3^, ^4^ Clinical applications of HSPC-GT include treatments of inherited immune deficiencies (e.g., X-linked severe combined immunodeficiency [SCID-X1], adenosine deaminase [ADA]-SCID, Wiskott-Aldrich syndrome), blood disorders (e.g., sickle cell disease, transfusion-dependent beta-thalassemia, X-linked chronic granulomatous disease), and lysosomal storage disorders (e.g., Hurler syndrome, Hunter syndrome, Fabry disease).^5^, ^6^, ^7^, ^8^, ^9^, ^10^, ^11^, ^12^, ^13^ In general, a patient’s bone marrow cells are mobilized and collected from the periphery, and HSPCs are isolated and transduced *ex vivo* with a lentiviral vector carrying a therapeutic payload. The genetically engineered cells are then intravenously infused back into the patient after a conditioning regimen using a myeloablative agent, such as busulfan. In mice, non-human primates, and humans, gene-modified HSPCs can engraft long term throughout the body, including within the hematopoietic compartment as cells of the bone marrow and blood, within the peripheral organs as tissue-resident macrophages, and within the brain and spinal cord as microglia-like cells (MLCs).^14^, ^15^, ^16^, ^17^

The migration of genetically engineered cells across the blood-brain barrier and their engraftment within the brain parenchyma is key to HSPC-GT’s ability to address neurological dysfunction. The conditioning agent regimen is thought to cause a partial depletion of microglia that can be repopulated by genetically modified MLCs that produce a therapeutic protein.^18^ These MLCs can secrete this therapeutic protein and thereby provide a local source for uptake by neighboring cells, such as neurons, astrocytes, and oligodendrocytes. Late-stage HSPC-GT clinical trials for metachromatic leukodystrophy (MLD) and cerebral adrenoleukodystrophy (CALD) have demonstrated halting of expected CNS-related disease progression, with most patients being free of severe motor and cognitive dysfunction.^19^, ^20^, ^21^ The fact that HSPC-GT can address the neurological symptoms of rare genetic diseases supports its possible translation to more common neurodegenerative disorders, such as Parkinson’s disease and dementia.

Still, a wider application of HSPC-GT to neurological diseases requires a deeper understanding of the nature and dynamics of genetically engineered MLCs and their engraftment in the brain. Unknowns include the extent and durability of MLC engraftment within the CNS, the effect of different routes of HSPC administration on MLCs, and the comparability of gene-modified MLCs to endogenous microglia. In the present study, we have addressed these key questions in preclinical murine models of HSPC-GT and demonstrated that this platform can provide expression of a transgene at potentially therapeutic levels throughout the periphery and the brain in two mouse models of neurodegenerative disease.

## Results

### Biodistribution of HSPC-GT-derived cells after intravenous (i.v.) and intracerebroventricular (i.c.v.) administration

**Figure 1:**
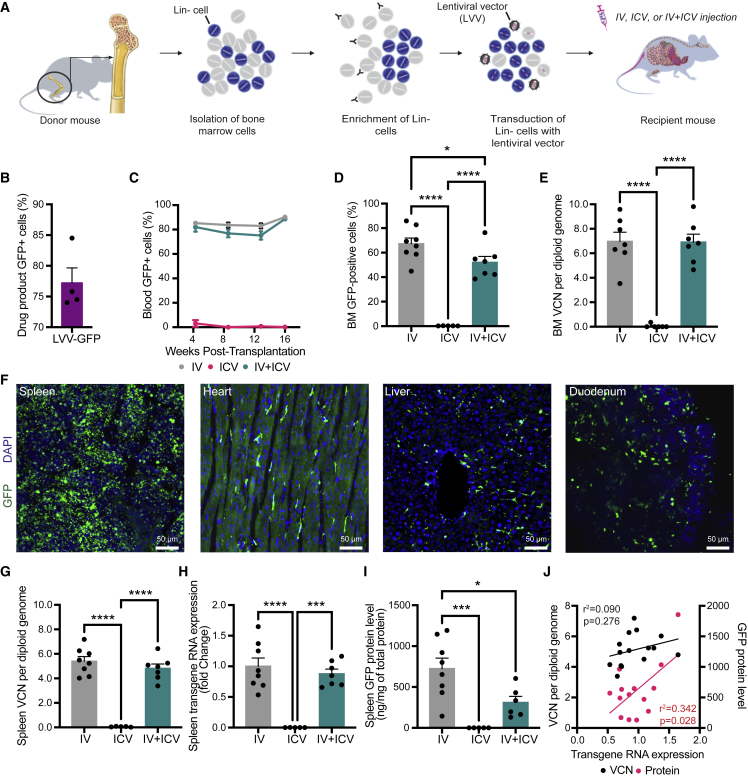
Peripheral engraftment HPSC-derived cells following administration via i.v., i.c.v., or i.v. + i.c.v. (A) Total bone marrow cells were isolated from donor mice, enriched for lineage-negative (Lin-) hematopoietic stem/progenitor cells, and then transduced with a lentiviral vector (LVV.GFP). Busulfan-conditioned recipient animals received cell doses via i.v., i.c.v., or both (i.v. + i.c.v.). (B–D) Flow cytometry for GFP was performed on the transduced Lin-drug product, at 4 week intervals on peripheral blood, and on the bone marrow (BM) at necropsy (16 weeks post-transplantation). (E) Vector copy number (VCN) in the BM was determined by qPCR. (F) Peripheral organs were harvested, fixed, sectioned, and GFP-positive cells were observed in all organs examined for all i.v.-dosed animals examined, including the spleen, heart, liver, and duodenum. No such engraftment was observed in any animals dosed via i.c.v. alone. (G–I) VCN, transgene RNA expression, and GFP protein levels were measured in spleens from each treatment group at 16 weeks post-treatment. (J) Correlation analysis between splenic levels of RNA and VCN (black dots and bar) and splenic levels of RNA and protein (red dots and bar) for animals that received doses by i.v. or i.v. + i.c.v. Bars represent means ± standard error of the mean (SEM), ∗ represent p < 0.05, ∗∗∗ represent p < 0.001, and ∗∗∗∗ represent p <0.0001. Closed circles in each graph represent individual data points. For (C)–(E) and (G)–(I), a one-way ANOVA with Tukey’s multiple post-hoc comparison test was conducted. For (J), the Pearson correlation coefficient for each correlation is shown.

Works in murine disease models have shown evidence of engraftment and functional rescue in the periphery and the brain after HSPC-GT using i.v. administration.^22^, ^23^, ^24^, ^25^, ^26^, ^27^, ^28^ Additionally, it has been shown that i.c.v. administration of genetically engineered HSPCs directly into the brain results in widespread engraftment of cells with many characteristics of endogenous microglia.^18^ However, the extent of engraftment and durability of HSPC-derived cells in the brain and how the route of administration effects the nature of engrafted cells are still poorly understood. To shine light on these crucial aspects, we performed a comprehensive animal biodistribution study following i.v., i.c.v., or a combination of i.v. and i.c.v. dosing of genetically engineered HSPCs into C57BL/6J mice after busulfan conditioning (Figure 1A).

Lineage negative (Lin-) HSPCs were isolated (Figures S1A and S1B) and transduced with a lentiviral vector encoding GFP (LVV.GFP) at greater than 74% transduction efficiency (range: 74.0%–84.5% GFP-positive cells; Figure 1B) and approximately 6 copies of integrated vector per genome (Figure S1C). No changes to the proliferative potential of the drug product were observed after transduction, as measured using the colony-forming unit assay Figure S1D). Recipient animals received 4 days of 25 mg/kg/q.d. (cumulative dose of 100 mg/kg) of busulfan prior to cell administration and lost 10%–15% of their bodyweight, which was rapidly regained post-treatment (Figure S1E). As expected, robust chimerism of all major cell types in the peripheral blood and bone marrow was observed in animals that received an i.v. dose of genetically engineered HSPCs but not in animals that only received genetically engineered HSPCs via i.c.v. (Figures 1C–1E and S2). Similarly, engraftment of cells throughout the peripheral organs was limited to animals that received an i.v. dose (Figure 1F). In the spleen, we detected integrated vector (Figure 1G), transgene expression (Figure 1H), and GFP protein expression (Figure 1I) at 16 weeks after infusion only in animals that received cells via an i.v. dose. As expected, we observed a significant correlation between the levels of GFP transcript and total amount of GFP protein in the periphery, though this correlation was not significant between vector copy number (VCN) and RNA (Figure 1J).

**Figure 2:**
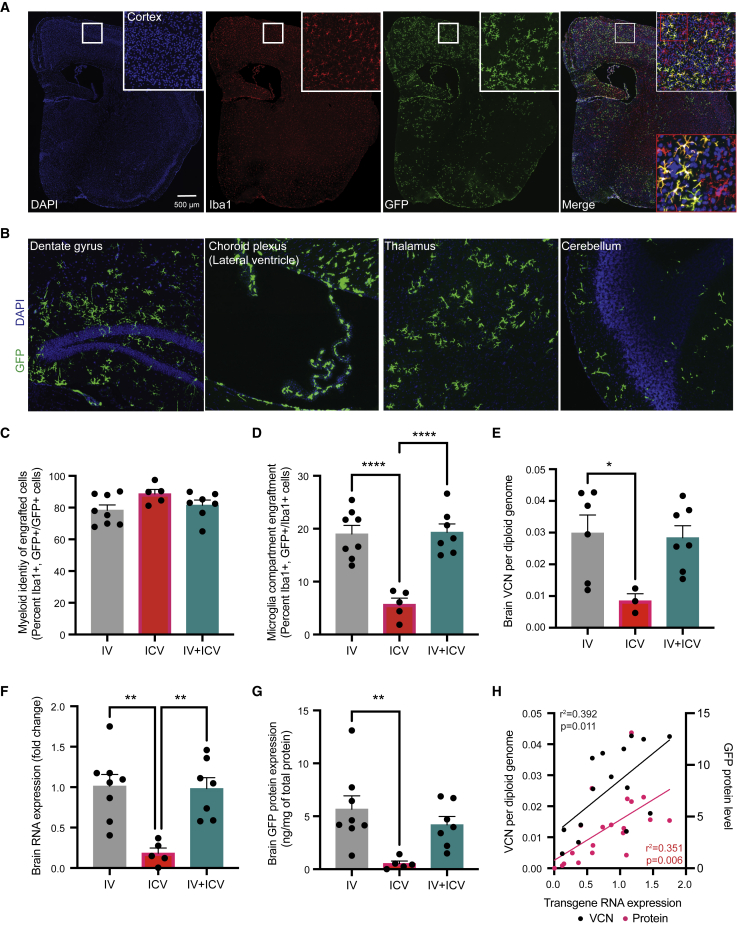
Engraftment of MLCs in the brain following different routes of administration of genetically engineered HSPCs (A) Representative image of a coronal slice from an i.v.-dosed animal visualizing Iba1, DAPI, and GFP at 16 weeks post-transplantation. Top right insert surrounded by white box is a digital magnification of a region of the primary motor cortex from the whole brain. Bottom right insert surrounded by red box is a magnification of the image in the white box. (B) Representative images from an i.v.-dosed animal at 16 weeks post-transplantation with GFP-positive cells located throughout the rostral-caudal axis of the brain, including the hippocampus, choroid plexus, thalamus, and cerebellum. (C) Quantification of GFP-positive engrafted cells that express the myeloid marker Iba1. (D) Quantification of Iba1+ cells that expressed GFP. (E–G) Quantification of integrated vector into the genome (VCN) via qPCR, transgene RNA expression via qRT-PCR, and GFP protein levels via ELISA in the brain for all treatment groups. (H) Correlation analysis of transgene RNA expression and VCN and transgene RNA expression and protein levels. Error bars represent means ± SEM, ∗ represent p < 0.05, ∗∗ represent p < 0.01, and ∗∗∗∗ represent p <0.0001. For (C)–(G), Tukey’s multiple post-hoc comparison test was conducted. For (H), the Pearson correlation coefficient for each correlation is shown.

Within the brain, all three routes of administration led to widespread engraftment of GFP-positive cells throughout the rostral-caudal axis, including the cortex, hippocampus, choroid plexus, thalamus, and cerebellum. Qualitatively, we observed widespread engraftment at 16 weeks post-transplantation (Figures 2A and 2B) and then again at 12–13 months (Figure S3A). At 16 weeks, 78%–89% of GFP-positive (GFP+) cells from all routes of administration co-expressed the microglial marker Iba1 (i.v.: 78.40% ± 3.30%; i.c.v.: 88.67% ± 2.78%; i.v. + i.c.v.: 81.57% ± 3.20%; Figure 2C). No significant differences in GFP+ cell engraftment was observed across the rostral-caudal axis for all routes of administration (Figure S4). Strikingly and in contrast to previous reports,^18^ we observed a significant difference in the level of engraftment between the i.v. and i.c.v. routes of administration. Animals dosed by i.v. alone had an average of 19.02% (range: 13.04%–25.44%) GFP+ engraftment in the microglia compartment compared with an average of 5.71% (range: 1.87%–8.26%) in animals dosed by i.c.v. alone (Figure 2D). Animals dosed using both routes of administration (i.v. + i.c.v.) had a similar level to animals dosed only via i.v. (range: 14.98%–26.64%; mean: 19.35% ± 1.57%; Figure 2D). Measurements of VCN per diploid genome (Figure 2E), transgene expression (Figure 2F) and GFP protein levels in the whole brain (Figure 2G) corroborated this difference in engraftment between i.v. and i.c.v. dosing. Statistically significant correlations were found between VCN, mRNA, and protein associated with the GFP transgene in the brain (Figure 2H). Of note, we observed a general lack of correlation of biodistribution metrics (VCN, mRNA, protein) in the peripheral tissues and the brain, consistent with previous clinical and preclinical reports^15^^,^^20^^,^^29^ that engraftment in these two compartments are independent events that occur from separate cell populations derived from the drug product (Figure S5).

### Single-cell transcriptional characteristics of HSPC-derived MLCs

The characteristics of HSPC-derived MLCs that engraft in the brain and their equivalence to microglia, which are derived from the embryonic yolk sac, remain contentious. Differences in the source of bone-marrow-derived progenitors (e.g., the bone marrow compartment versus an administered cell bolus), the nature of cell recruitment (e.g., native signaling versus induction from microglial ablation), and the state of the brain (e.g., healthy versus active disease) all influence MLCs and how they might compare to *bona fide* microglia. While transcriptional signatures that separate HSPC-derived MLCs and microglia have been described broadly, the single-cell heterogeneity of these populations and a high-resolution characterization of these differences in HSPC-GT have remained uncovered to date. Similarly, the comparison of transcriptional signatures of long-term engrafted MLCs that engraft via migration from the periphery (i.v. dosed) versus those that directly administered to the brain (i.c.v. dosed) has not been described.

**Figure 3:**
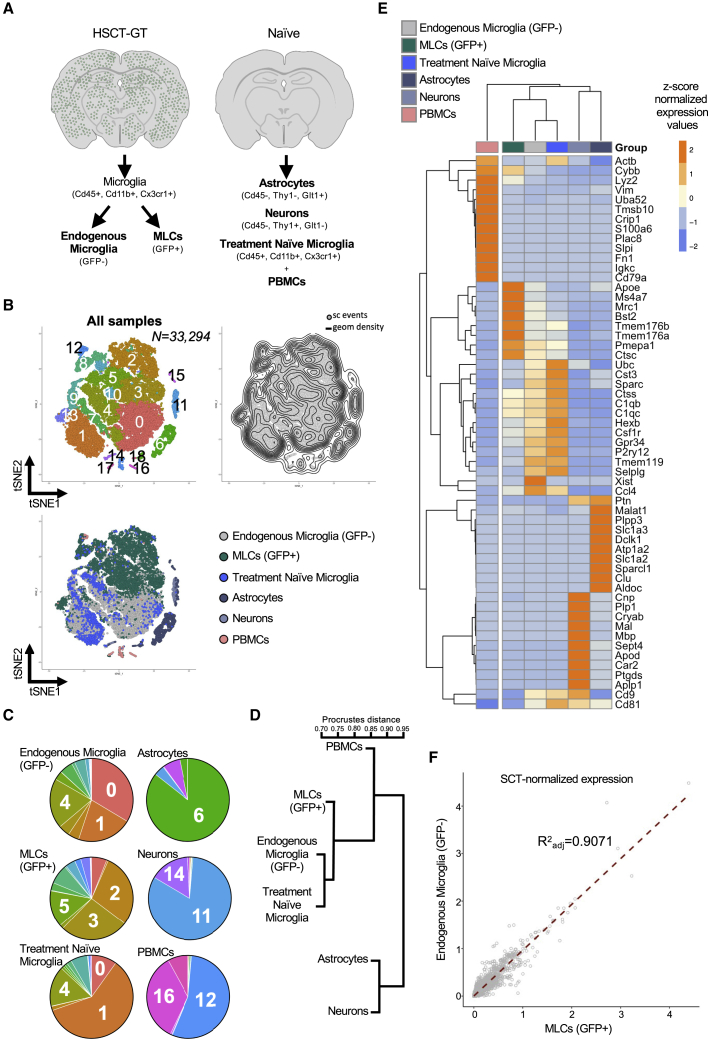
Single-cell comparison of MLCs, endogenous microglia, PBMCs, neurons, and astrocytes (A) MLCs and endogenous microglia were isolated via enzymatic digestion, FACS purified, and then single-cell sequenced from i.v.- and i.c.v.-dosed male and female animals. PBMCs, neurons, astrocytes, and microglia were similarly isolated from a treatment-naive animal. (B) Global tSNE plot generated from a total of 33,294 cells (n = 5 mice; 12 samples). The plot on the left shows the Louvain clustering (resolution 0.4), while the plot on the right shows the density of single-cell events on the map. (C) Pie charts showing the cluster breakdown by sample, with labels showing the top represented clusters for each sample type. Seven MLC-enriched clusters (2–5, 8, 10, 12, 13, 15), 5 microglia-enriched clusters (0, 1, 4, 7, 9), 1 neuron cluster (11), 1 astrocyte cluster (6), and 4 PBMC clusters (14, 16–18) were identified. (D) Unsupervised clustering based on the global expression profile of each sample type. (E) Heatmap showing top 10 differentially expressed genes for each sample type. Dendrogram at the top shows samples clustering based on expression profiles of these genes. (F) Correlation analysis of endogenous microglia versus MLCs based on normalized single-cell gene-expression data.

To address these aspects, we combined cell sorting with high-throughput single-cell RNA sequencing (scRNA-seq) to characterize long-term engrafted MLCs and endogenous microglia from the same animals. We isolated endogenous microglia (GFP-negative [GFP-]) and MLCs (GFP+) from enzymatically dissociated whole brains by fluorescence-activated cell sorting (FACS) based on the expression of cell surface markers (Cd45+, Cd11b+, Cx3cr1+) from one male and one female mouse 12–13 months after either i.v. or i.c.v. administration of HSPCs (Figures S3B–S3G). After sequence processing and data analysis, we obtained a total of 29,085 individual scRNA profiles across the eight combined datasets for MLCs and endogenous microglia. We also generated comparator datasets from a 12-week-old, treatment-naive C57BL/6J animal for several cell populations: microglia (Cd45+, Cd11b+, Cx3cr1+), neurons (Cd45-, Thy1+, Acsa2+), astrocytes (Cd45-, Thy1-, Acsa2+), and peripheral blood mononuclear cells (PBMCs; Figure 3A). By t-stochastic neighbor embedding (tSNE) dimensionality reduction of the most variable genes, we observed that MLCs are in distinct but neighboring clusters to endogenous microglia (Figures 3B, 3C, and S6). The majority of PBMCs, neurons, and astrocytes occupied largely distinct single-cell clusters compared with microglia and MLCs except, notably, a population of PBMC circulating monocytes, which cluster with MLCs. Hierarchical clustering analysis confirmed that MLCs are similar to endogenous microglia and share higher similarity with bone-marrow-derived PBMCs than with neurons and astrocytes (Figures 3D and 3E). We then directly compared the normalized levels of individual transcripts in all GFP+ MLCs with that of their endogenous GFP- microglia counterparts to assess the degree of similarity between these two populations. We observed a strong correlation across most transcripts (Figure 3F; adjusted R^2^ = 0.9071), supporting the notion that the transcriptome of HSPC-derived MLCs are distinguishable but largely similar to that of endogenous microglia.

**Figure 4:**
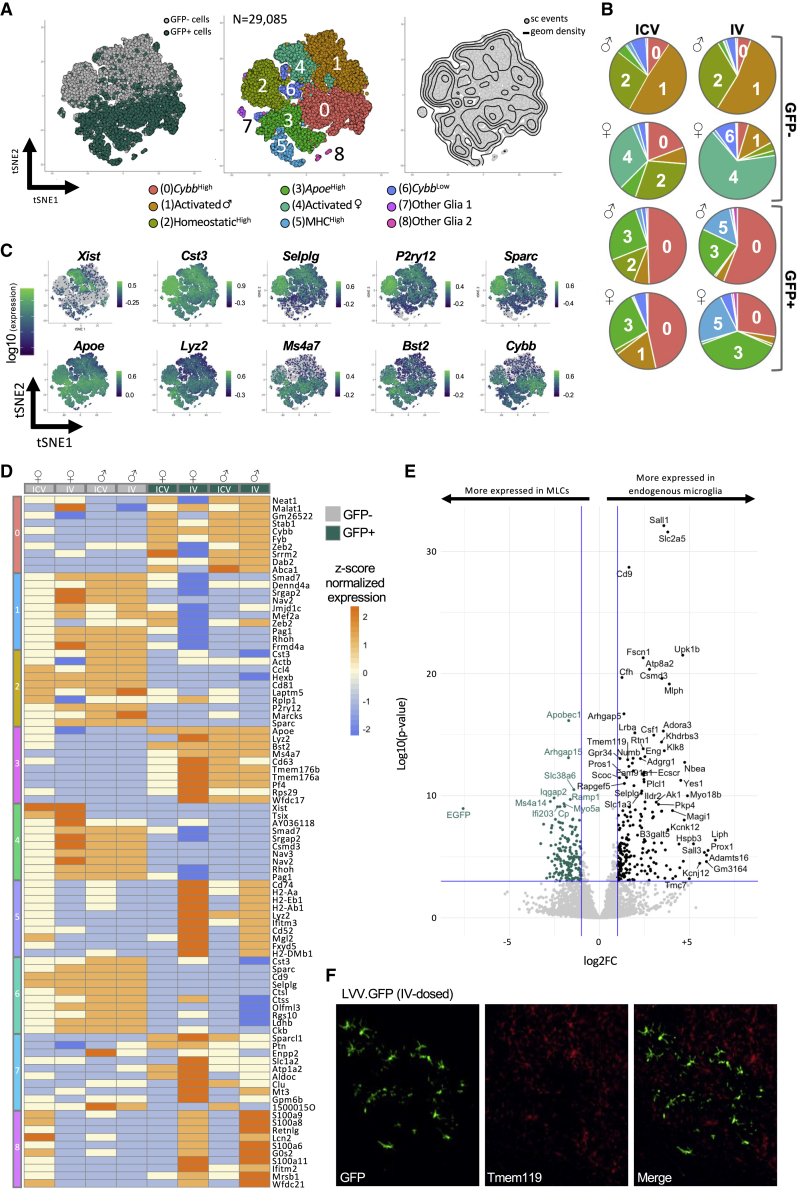
Characterization of the single-cell transcriptional landscape of MLCs versus endogenous microglia in treated mice (A) Global tSNE plot generated from a total of 29,085 single cells from endogenous microglia (GFP) and MLCs (GFP+, in green) (n = 4 mice; 8 samples). The plot on the left shows single cells separated in the main GFP- (gray dots) and GFP+ (green dots) groups. The plot in the middle shows the Louvain clustering (resolution 0.2). Putative functional associations and/or key markers differentiating each cluster are listed below. The plot on the right shows the density of single-cell events in the map. (B) Feature plots showing expression of individual genes characterizing the top (microglia enriched) or the bottom (MLC enriched) sections of the map. (C) Heatmap showing single-cell normalized expression of the top 10 differentially expressed for each cluster compared with the rest of the map (note: clusters might share top represented genes). Columns represent GFP- (gray squares) and GFP+ (green squares) cells from treated mice labeled according to their sex, identification number, and route of administration (i.c.v., i.v.). (D) Volcano plot highlighting differentially expressed genes between GFP+ MLCs (left-hand side highlighted in green) and GFP+ microglia (x axis log2 fold change in average gene expression, y axis log). (E) Representative IHC images of GFP and Tmem119 from an i.v.-dosed animal.

We then analyzed in detail the single-cell heterogeneity of GFP+ MLCs and GFP- endogenous microglia in our treated mice. By tSNE dimensionality reduction of highly variable genes and Louvain clustering of single cells, we detected 8 main single-cell clusters when comparing these two populations. Notably, we observed a clear separation of MLC and microglia populations using clustering analysis (Figures 4A, 4B, and S7). GFP- endogenous microglia were enriched in clusters 1, 2, 4, and 6, while GFP+ MLCs were enriched in clusters 0, 3, and 5. Clusters 7 and 8 were also composed of a very small number of contaminating glia (astrocytes and oligodendrocytes), which we preserved as an outgroup comparison. The top 10 significantly enriched genes defining each cluster highlight the developmental differences between MLCs and microglia and potential differences in their inflammatory state (Figures 4C and 4D). Clusters containing mostly GFP- cells were enriched for markers associated with microglia homeostasis including *Mef2a*, *Hexb*, *Ctsc3*, *P2ry12*, *Selplg*, and *Sparc*, with this signature being most highly enriched in clusters 2 and 6. Clusters 1 and 4 showed relatively lower levels of homeostatic genes, a hallmark of mild microglia activation that is likely in part a result of the enzymatic isolation process. Notably, cluster 1 clearly separates from cluster 4 based almost entirely on the sex of the recipient animal, with differences driven largely by the expression of the X-inactivation genes *Xist* and *Tsix*. This sex difference was not observed in GFP+ MLC-enriched clusters, given that all donor animals for this experiment were males (Figure S8). Clusters containing mostly GFP+ MLCs were enriched with core transcriptional markers previously associated with bone-marrow-derived “CNS-associated macrophages” (CAMs) *Ms4a7*, *Mrc1*, *Pf4*, and *Stab1*.^30^ Moreover, GFP+ enriched clusters also show stronger expression of activation markers of endogenous microglia including genes associated with reactive oxidation (*Cybb*, *Fyb*; cluster 0) and metabolism (*Apoe*, *Lyz2*; cluster 3) and genes that encode for the major histocompatibility complexes (MHCs) such as *Cd74*, *H2-Aa*, *H2-Eb1*, and *H2Ab1* (cluster 5).

To identify highly expressed markers that could distinguish MLCs from endogenous microglia, we first performed a differential gene expression analysis comparing the composite single-cell transcriptome of GFP+ and GFP- cells (Figure 4E). Homeostatic genes used as canonical microglia markers including *SallI*, *Csf1*, and *Tmem119* were enriched in GFP- endogenous microglia over GFP+ MLCs. Accordingly, Tmem119 expression was evident in endogenous microglia but largely excluded from GFP+ cells in the brain (Figure 4F). Genes enriched in MLCs include *Apobec1* and *Ms4a14*, markers that have been previously associated with bone marrow (BM)-derived, CAMs/peripheral nervous system (PNS)-associated macrophages when compared with endogenous microglia.^31^^,^^32^ Gene Ontology (GO) and pathway analysis comparing the two populations highlight differences in genes associated with immune function, including chemokine production, cell adhesion, and responses to interferon, all processes associated with the known functional roles of endogenous microglia in the brain (Figure S9).

**Figure 5:**
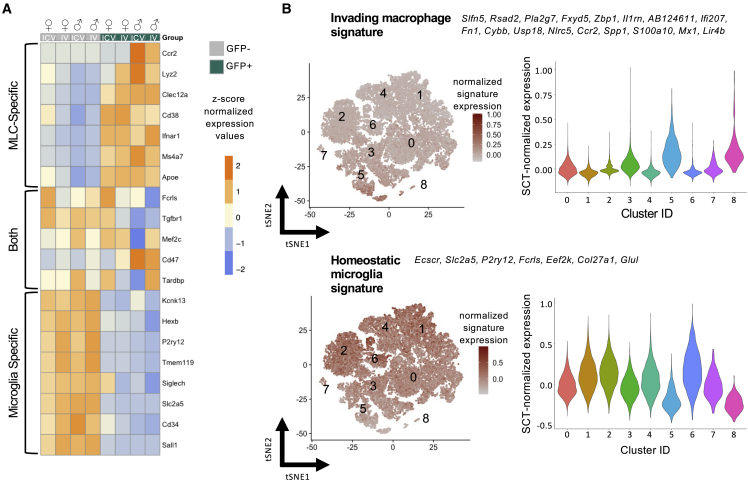
Projection of MLC-, macrophage-, and microglia-specific markers on the single-cell landscape (A) Heatmap showing the single-cell normalized average expression values of MLC-specific, microglia-specific, and shared gene markers as described in Shemer et al.^29^ (2018) in GFP- and GFP+ cells from treated mice. (B) Projection of invading versus homeostatic microglia gene signatures as described in DePaula-Silva et al. (2019) over the tSNE map of Figure 4A (left panels), and violin plots showing the expression of these signatures in the clusters identified in Figure 4A (right panels). (C) Violin plots showing the expression of genes associated with invading macrophages/monocytes (left panels) or homeostatic microglia (right panels) as described in Haage et al.^33^ (2019) in GFP- (gray squares) or GFP+ (green squares) treated mice labeled according to their sex and identification number.

Our analysis comparing MLCs and microglia confirms and expands upon the information from previous studies on CAMs/PNS-associated macrophages, where expression of markers associated with inflammation in parenchymal microglia are enriched in BM-derived macrophages isolated from healthy animals without overt neuroinflammation.^29^^,^^32^ In addition, our data show clear concordance with markers previously identified using bulk RNA-seq comparing MLCs and microglia after busulfan conditioning and i.v. dosing of HSPCs (Figure 5A).^29^ We then compared our datasets to previous work characterizing the transcriptional profiles of CNS-invading, inflammatory macrophages that are mobilized from the periphery in the context of disease or injury.^33^ Our comparison using this gene list associated with invading macrophages showed that our GFP+ MLCs show generally low expression of genes associated with invading macrophages and relatively higher expression of the homeostatic microglia signature (Figure 5B). These results suggest that certain differences we observed between MLCs and microglia may result from their distinct developmental origin rather than from distinct activation states.

Lastly, we wanted to investigate whether the route of administration impacted the transcriptional profile of MLCs. Surprisingly, and despite a vastly different engraftment history, we observed a significant overlap in the transcriptional signatures of i.v.-dosed GFP+ and i.c.v.-dosed GFP+ cells, with most transcripts showing strong correlation in expression (Figure S10A; R^2^ = 0.9134). i.v. and i.c.v. GFP+ cells had similar representation in each cluster, apart from cluster 5 (MHC^high^), which was enriched for i.v. GFP+ cells over i.c.v. GFP+ cells (Figure 4B). Expression of specific MHC genes was previously reported to be associated with border-associated macrophages (BAMs), which are largely yolk-sac-derived macrophages that occupy a niche near the blood-brain barrier.^34^ This signal is consistent with the engraftment history of i.v.-derived MLCs, which must migrate across the blood-brain barrier. Accordingly, clustering analysis show differences between i.v. and i.c.v. groups in the expression of several MHC genes, including *H2-a*, *H2-b*, and *Cd74* (Figure S10B).^35^

### LVV.GBA and LVV.GRN increase GCase/progranulin expression and secretion

**Figure 6:**
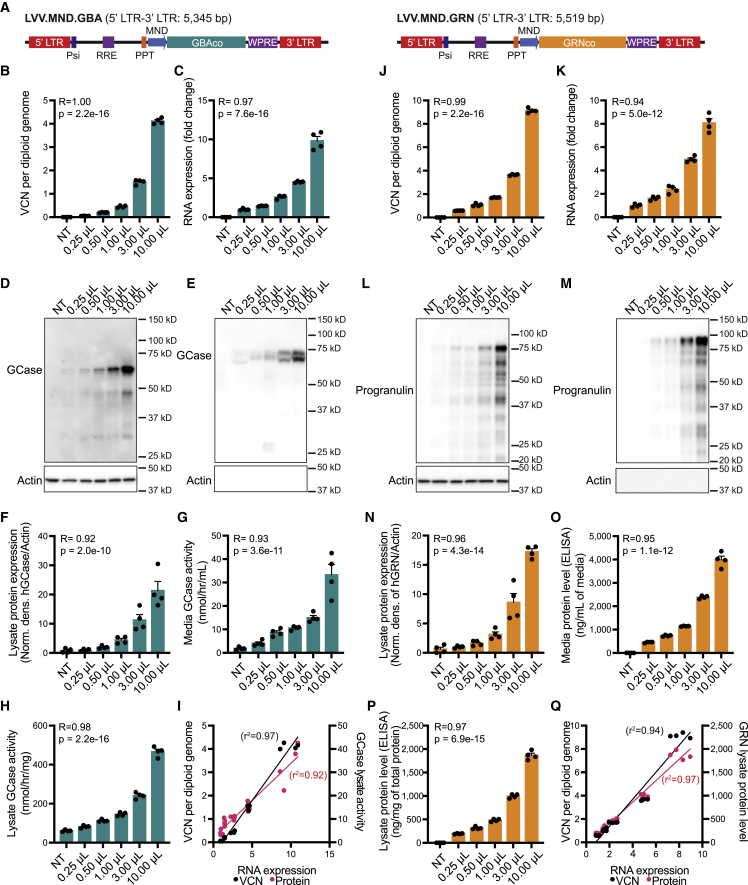
Characterization of lentiviral vectors for GBA and GRN in a mouse macrophage cell line (A) Schematic of integrating component of lentiviral vectors from the 5′ long terminal repeat (LTR) to 3′ LTR. RRE, Rev response element; PPT, polypurine tract; MND promoter, myeloproliferative sarcoma virus enhancer, negative control region deleted, dl587rev primer-binding site substituted; WPRE, woodchuck hepatitis virus posttranscriptional regulatory element. (B–I) LVV-GBA increased integrated VCN (B), transgene RNA expression (C), GCase protein expression in lysates (D and F) and media (E), GCase enzymatic activity levels in the lysate (H) and conditioned media (G). Correlation analysis of VCN per diploid genome to transgene RNA expression and transgene RNA expression to lysate GCase activity (I). (J–Q) LVV-GRN increased integrated VCN (J), transgene RNA expression (K), and progranulin protein levels in the lysate (L, N, and P) and conditioned media (M and O). Correlation analysis fo VCN per diploid genome to transgene RNA expression and RNA transgene expression to lysate GRN protein levels (Q). Bars represent means ± SEM. Pearson correlation analysis is shown where indicated, correlating the VCN to RNA expression for cells transduced with LVV.GBA (I) and LVV.GRN (Q).

Our characterization of MLCs and our assessment of biodistribution of gene-modified cells supports a wider application of HSPC-GT to address the peripheral and CNS components of neurodegenerative disease with well-defined genetic risk factors. Two such disorders are GBA-associated Parkinson’s disease (GBA-PD) and progranulin-associated frontotemporal dementia (GRN-FTD), both of which are associated with the heterozygous loss of function of a lysosomal protein (beta glucocerebrosidase and progranulin, respectively). We generated lentiviral vectors expressing either codon-optimized human GBA (LVV.GBA) or codon-optimized human GRN (LVV.GRN) (Figure 6A) and transduced mouse macrophage RAW264.7 cells with increasing amounts of vector. Increased integrated vector copies per diploid genome predictably increased the amount of transgene-derived RNA transcript and protein expression for both vectors (Figures 6B–6Q). Importantly, we observed active beta-glucocerebrosidase (GCase) and human progranulin (hGRN) in the conditioned media even at low VCNs, supporting that lentiviral-mediated, supraphysiological expression of these proteins results in their secretion from macrophage-like cells (Figures 6E, 6G, 6M, and 6O). This suggests that GCase or progranulin could be secreted by MLCs for uptake by other cells within the brain.

### HSPC-GT in a model of GBA-PD results in widespread distribution of gene-modified cells

**Figure 7:**
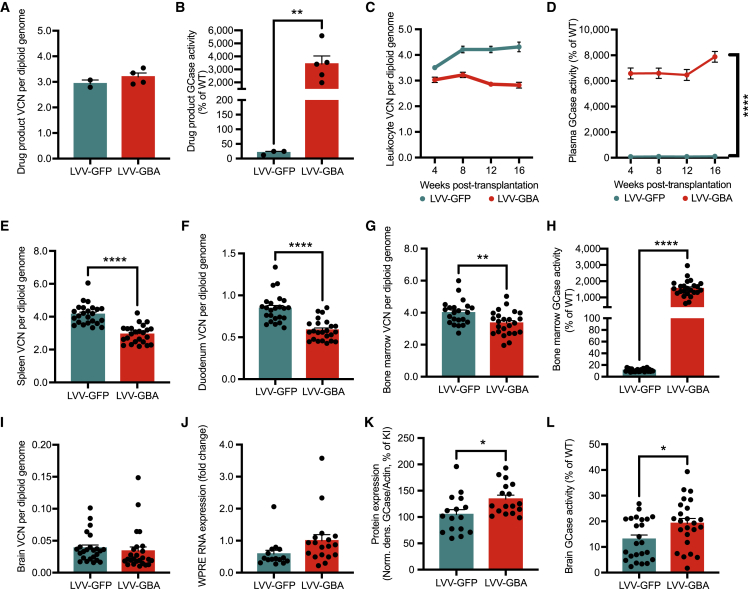
Biodistribution analysis after transplantation of LVV.GBA modified HSPCs into *Gba* mutant mice (A) VCN in the drug product in the LVV-GFP- and LVV-GBA- treated groups kept in culture for 4 days *in vitro* (DIV). (B) GCase enzymatic activity in the drug product at DIV 4. (C and D) VCN and GCase enzymatic activity in white blood cells and plasma, respectively, measured longitudinally every 4 weeks post-transplantation. (E and F) VCN in spleen and duodenum at 16 weeks post-transplantation. (G and H) VCN and GCase enzymatic activity in the BM at 16 weeks post-transplantation. (I–L) VCN, transgene RNA expression, GCase protein levels, and GCase enzymatic activity in the brain at 16 weeks post-transplantation. Bars represent means ± SEM, ∗ represent p < 0.05, ∗∗ represent p < 0.01, and ∗∗∗∗ represent p <0.0001. For (A), (B), and (E–L), t test was used for statistical analysis.

Mutations in *GBA* are causative for the lysosomal storage disorder Gaucher disease and are among the most prevalent genetic risk factors for PD^36^^,^^37^ and Lewy body dementia.^38^ To assess the utility of lentiviral HSPC-GT for patients with PD associated with a *GBA* mutation (GBA-PD), we conducted a biodistribution study of key organs in homozygous *Gba*^D409V^ mice, a model of GBA-PD.^39^ We isolated Lin- cells and transduced them with either a lentiviral vector encoding our previously characterized human GBA transgene (LVV.GBA) or a control GFP transgene (LVV.GFP) at a similar number of vector integrations per diploid genome (LVV.GBA: 3.12 ± 0.15, LVV.GFP: 2.93 ± 0.14; Figure 7A). As expected, the GCase activity in the cells transduced with the GBA transgene had significantly higher activity than those transduced with the GFP transgene (LVV.GBA: 3,418.10% ± 612.2%, LVV.GFP: 20.38% ± 4.31%; Figure 7B).

We then transplanted the genetically modified HSPCs into homozygous *Gba*^D409V^ knockin mice, which have lower levels of enzyme activity in the BM and brain compared with wild-type controls (Figures S11A–S11D). Transplantation of LVV.GBA HSPCs led to detectable and stable levels of VCNs in white blood cells (Figure 7C). Furthermore, we observed GCase activity in the plasma starting at 4 weeks post-transplantation (earliest time point) through 16 weeks (terminal time point). In contrast, there was minimal, if any, GCase activity in LVV.GFP-treated animals (Figure 7D). At 16 weeks, we observed engraftment in the spleen (Figure 7E), duodenum (Figure 7F), and BM (Figure 7G) in both LVV.GBA- and LVV.GFP-treated animals. Importantly, we measured higher levels of GCase activity in the BM of LVV.GBA-treated animals compared with animals transplanted with LVV.GFP HSPCs (LVV.GBA: 1,547.75% ± 102.2%, LVV.GFP: 11.06% ± 0.50%; Figure 7H). Within the brain, we saw detectable levels of integrated vector (Figure 7I) and transgene RNA expression (Figure 7J) in both animal groups. We observed higher levels of GCase protein (LVV.GBA: 134.4% ± 7.24%, LVV.GFP: 105% ± 9.16%; Figure 7K) and GCase enzymatic activity (LVV.GBA: 19.19% ± 1.93%, LVV.GFP: 13% ± 1.63%; Figure 7L) in the LVV.GBA cohort versus the LVV.GFP cohort. Taken together, we observed widespread and robust biodistribution of the gene-modified cells in key organs, including the gastrointestinal tract, spleen, BM, and CNS.

### HSPC-GT in a model of GRN-FTD increases progranulin and may address lysosomal dysfunction

**Figure 8:**
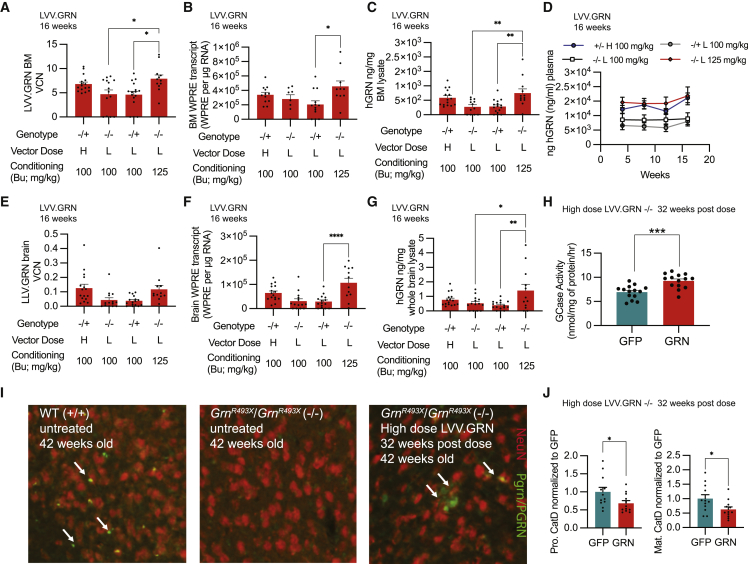
Genetically engineered HSPC administration in a mouse model of GRN-FTD shows widespread GRN in the periphery and the brain (A–C) VCN, transgene RNA, and human progranulin protein measurements in BM for all LVV.GRN-treated groups at 16 weeks post-transplantation. (D) Longitudinal human progranulin protein measurements in plasma for all LVV.GRN-treated groups up to 16 weeks post-transplantation. (E–G) VCN, transgene RNA, and human progranulin protein measurements in brain for all LVV.GRN-treated groups at 16 weeks post-transplantation. (H) GCase activity in whole brain lysate of homozygous mutant *Grn* mutant mice treated with LVV.GFP and LVV.GRN at 32 weeks post-transplantation. (I) IHC imaging of neurons (NeuN, red) and progranulin (PGRN/Pgrn, green) in the cortex of untreated wild-type, homozygous mutant *Grn* mutant mice, or homozygous mutant *Grn* mutant mice treated with LVV.GRN. (J) Quantification of levels of the pro form and the mature form of cathepsin D (CatD) in whole-brain lysates of homozygous mutant *Grn* mutant mice treated with LVV.GFP and LVV.GRN at 32 weeks post-transplantation. Bars represent means ± SEM. Tukey’s multiple post-hoc comparison test was conducted for all statistical measures shown, with only significant differences (p < 0.05) indicated (∗ represent p < 0.05, ∗∗ represent p < 0.01, ∗∗∗∗ represent p <0.0001).

We then applied our HSPC-GT platform to treat progranulin deficiency in a mouse model of genetic FTD (GRN-FTD). GRN-FTD is a familial form of neurodegeneration caused by the haploinsufficiency of GRN/progranulin, a lysosomal precursor protein that is widely expressed in the brain and secreted from microglia during overt disease.^40^ Heterozygous and homozygous *Grn*^*R493X*^ mutant mice, a model of GRN-FTD,^41^ were dosed with Lin- cells transduced with high or low vector doses of LVV.GFP or LVV.GRN after standard (100 mg/kg) or increased (125 mg/kg) levels of busulfan conditioning. As in our previous studies, we observed biodistribution via vector integration quantification and transgene expression in the BM and the brain for all treatment groups for LVV.GFP and LVV.GRN (Figures 8A, 8B, 8E, 8F, and S12A–S12D). Across all LVV.GRN-treated groups, we measured supraphysiological levels of human progranulin via ELISA in the plasma and the BM, equivalent to an average of 5-to 11-fold above previously reported wild-type levels (Figures 8C and 8D).^42^ We also observed human progranulin levels in whole-brain lysate of LVV.GRN-treated animals, equivalent to 30%–50% of previously reported mouse progranulin levels in the brain (Figure 8G).^43^ Using immunohistochemical visualization of mouse and human progranulin, we observed progranulin+ cells in the cortex of LVV.GRN-treated homozygous mutant animals, consistent with the engraftment of LVV.GRN+ cells in the brain (Figure 8I). We observed no progranulin staining in treatment-naive *Grn* mutant mice and saw restoration of progranulin puncta after LVV.GRN HSPC-GT. Many, but not all, of these cells colocalized with the microglia marker Iba1, consistent with our previous experiments (Figure S13). Finally, and notably, increased levels of busulfan conditioning resulted in significantly increased VCNs, transgene expression, and GRN protein levels in the periphery and the brain (Figures 8A–8G).

While the reasons GRN deficiency leads to FTD are still unclear, progranulin is thought to play a role in regulating lysosomal homeostasis.^43^ The protein and activity levels of several lysosomal enzymes, including decreased GCase and increased cathepsin D (CatD) have been observed in mouse models of GRN-FTD and human brain tissue from GRN-FTD patients.^44^^,^^45^ Other models of GRN-targeted therapeutics have shown the ability to normalize these changes after treatment.^44^ We observed similar changes of lysosomal dysfunction after treatment, with LVV.GRN-treated homozygous *Grn* mutant animals 32 weeks post-transplantation showing an average of 33% increased GCase activity levels compared with LVV.GFP-treated groups (Figure 8H) and decreased mature CatD protein (Figure 8J) compared with LVV.GFP-treated groups.

## Discussion

We show here that HSPC-GT using an i.v. route of administration in mice results in the widespread engraftment of genetically engineered cells throughout the periphery and engraftment of MLCs in the brain. Similar levels of engraftment in the brain were previously reported by others.^28^^,^^46^ In contrast, when HSPCs are administered via i.c.v., we observed that engraftment is limited to the CNS. Strikingly, and in contrast to earlier work,^18^ we observed significantly more engrafted MLCs using i.v. administration compared with i.c.v. administration. While our methods and time points were similar for all key aspects, subtle technical disparities could explain the difference (e.g., cell dose, injection location, and/or cell-administration timing). Further studies are needed to explore these parameters and how they affect engraftment after i.c.v. administration of HSPCs, if at all. We also observed that HSPC-derived MLCs were durably engrafted within the brain over a year post-transplantation and that these cells bore many of the transcriptional hallmarks of microglia, which suggests that they have the capacity to self-renew much like endogenous microglia, though careful lineage-tracing experiments would be required to explicitly confirm this. This is consistent with long-term follow up of HSPC-GT in non-human primates, where HSPC-derived myeloid cells in the brain were observed 10 years post-transplantation after i.v. dosing.^15^ It is similarly consistent with the durable phenotypic rescue in most patients enrolled in clinical trials for MLD and CALD who receive their cell doses i.v.^47^ Importantly, these data strongly support the use of murine models for preclinical and translational research, as they can effectively recapitulate the long-term engraftment dynamics of MLCs observed in humans.

Our comparison of MLCs with endogenous microglia suggests that most transcriptional features of myeloid cells in the brain are niche dependent, as the two cell populations are largely similar. This observation parallels the ones from studies where monocytes and macrophages isolated from various tissues take on functional and transcriptional characteristics of tissue-resident macrophages of the lung, kidney, and liver when transplanted.^48^, ^49^, ^50^, ^51^ Furthermore, we show here that MLCs do not exhibit the signatures of invading macrophages associated with disease and injury, likely because these signatures are niche dependent and reflect the damaged status of the brain. Our data show, however, that MLCs and microglia can be clearly distinguishable by specific genes not reprogrammed during engraftment, such as canonical genes associated with resident microglia like *Tmem119*. It is likely that this is in part driven by the developmental differences between BM-derived myeloid cells and embryonic yolk-sac-derived microglia. How HSPC-derived MLCs and microglia differ on a functional level as a result of these differences in developmental origin will require further study, though as stated, our characterization shows that the transcriptional signatures are highly similar. The differences we observe in MLCs versus endogenous microglia will be informative for the further development of MLCs as a therapeutic modality and to understand what may underlie any functional differences between them and endogenous microglia.

Our work applying HSPC-GT in murine models of neurodegenerative disease shows that for both GBA-PD and GRN-FTD, engrafted cells have the capacity to produce potentially therapeutic proteins at significant levels. Notably, gastrointestinal issues are a common symptom in patients with PD, and mutations in *GRN* are implicated in cardiac dysfunction.^52^^,^^53^ A systemic GT approach like HSPC-GT conceivably has advantages over other platforms including AAV GTs, where therapeutic effect is generally limited to either the periphery or the CNS. However, if CNS-restricted expression is therapeutically advantageous, our data suggest that i.c.v. administration of genetically engineered HSPCs can achieve this.

Furthermore, previous work has demonstrated that AAV administration into the brain parenchyma leads to extremely high levels of transgene expression close to the injection site but a vastly lower expression level in distal brain regions.^54^^,^^55^ Importantly, the cell type that expresses the therapeutic protein will depend on the AAV capsid and promoter used.^56^ Conversely, we showed here that the tiling behavior of MLCs expands the reach of HSPC-derived cells to the whole brain, resulting in an even and widespread distribution of genetically modified cells. It is worth noting that AAV-mediated delivery of GBA and GRN to neurons in mouse models of GBA-PD and GRN-FTD have been successful in modifying disease-associated phenotypes.^44^^,^^57^ Our biodistribution studies and our initial biochemical characterization of lysosomal dysfunction in *Grn* mutant mice support the capacity of reaching similar results with HSPC-GT. Further characterization is required to determine (1) whether there are potential changes to the characteristics of MLCs carrying a therapeutic transgene and (2) if HSPC-derived MLCs can provide the levels and modality of transgene expression necessary to correct the wider functional changes in the brain associated with these disorders, with careful inclusion of comparison to wild-type animals. These follow-up study will elucidate the true therapeutic potential of this platform.

While this work addresses fundamental questions around HSPC-GT in the CNS, such as the identity of MLCs relative to endogenous microglia and the durability of MLC engraftment, there are broader questions that remain unresolved. These include understanding how the myeloablative agent acts in the CNS. Our results clearly demonstrate that busulfan conditioning is instrumental in enabling brain engraftment, with higher doses leading to higher engraftment and protein expression. Notably, busulfan conditioning may also be associated with cellular senescence of endogenous microglia, making increased busulfan conditioning a potential detriment despite increased engraftment.^58^ Recent preclinical work has highlighted the use of CSF1R inhibition after treatment with busulfan, reporting up to 90% engraftment of the microglial niche, suggesting the potential for using other conditioning regimens outside of busulfan.^59^ In addition, the identity of the cell that crosses the blood-brain barrier (e.g., HSPC, myeloid progenitor cell) after i.v. administration or that first engrafts after i.c.v. administration is unclear. It is also worth examining if injection of myeloid cells alone could achieve similar levels of engraftment. Future studies should assess what the window of opportunity is for cells to cross the blood-brain barrier following myeloablation and how genetic payloads might change engraftment of MLCs.

In conclusion, our work provides critical information substantially expanding our understanding of the nature of MLCs derived from long-term HSPCs and opens new avenues for the potential use of genetically engineered MLCs for the treatment of common neurodegenerative disorders such as FTD and PD.

## Materials and methods

### Mouse models and tissue collection

All experiments were carried out with proper oversight by institutional review boards for animal care. Comparisons of i.v. and i.c.v. routes of administration were completed using wild-type (WT) C57BL/6J mice (Jackson Laboratory, stock number 000664). Proof-of-concept experiments for GBA-PD were completed using homozygous *Gba*^*D409V/D409V*^ mutant mice (Jackson Laboratory, C57BL/6N-*Gba*^*tm1*.*1Mjff*^/J, stock number 019106)^39^ and WT C57BL/6NJ mice (Jackson Laboratory, stock number 005304) as controls. Proof-of-concept experiments for GRN-FTD were completed using heterozygous and homozygous *Grn*^*R493X*^ mutant mice on the C57BL/6J background.^41^ Donor and recipient animals for all experiments were 8–12 weeks of age. Terminal collection for all animals occurred 14–16 weeks after cell administration except for the long-term cohort for scRNA-seq experiments (12–13 months post-transplantation) and the long-term cohort for the GRN-FTD proof-of-concept study (32–36 weeks post-transplantation). At necropsy, animals first underwent whole-body transcardial perfusion with either heparinized saline or PBS followed by tissue harvesting. Samples for biochemistry analysis were flash frozen and stored at -80°C. Samples for immunohistochemistry (IHC) analysis were fixed in 4% PFA in PBS overnight at 4°C.

### Generation of genetically engineered HSPC drug product

For all studies, Lin- cells were enriched from total BM isolated from femurs and tibias of donor mice (6–12 weeks of age) using the EasySep Mouse Hematopoietic Progenitor Cell Isolation Kit (STEMCELL Technologies) in conjunction with the RoboSep-S (STEMCELL Technologies) automated cell-isolation machine and confirmed by flow analysis. Cells were stained for lineage markers with a PE-Cy5-conjugated lineage marker cocktail including B220, Ter119, Tcrb, Cd8a, Cd3ε, Cd4, Ly6g/Ly6c, and Cd11b and with hematopoietic stem cell markers APC-conjugated anti-Cd117 (c-Kit) and PE-conjugated Ly6a/e (Sca-1) (BD Biosciences). Lin- cells were transduced with a lentiviral vector in a cell incubator at 2 × 10^6^ cells/mL in either StemSpan SFEM II (STEMCELL Technologies) or StemMACS HSC Expansion Media (Miltenyi Biotec) growth media freshly supplemented with TPO (10 ng/mL), SCF (100 ng/mL), and FLT3 (50 ng/mL). Approximately 16–22 h later, cells were collected, washed at least three times with DPBS (without Ca^2+^ and Mg^2+^), and resuspended in an appropriate volume for dosing. A small number of cells (3,000–4,000 cells) were set aside for use in the colony-forming unit assay using M3434 media (STEMCELL Technologies) and were analyzed 7–10 days later.

### Conditioning of recipient animals and drug-product administration

Four days prior to cell administration, recipient animals (6–12 weeks of age) received daily i.p. injections of busulfan (Busulfex, Otsuka Pharmaceutical) at 25 mg/kg for a cumulative dose of 100 or 125 mg/kg. For comparisons of routes of administration, drug-product pools were generated from an equal number of male and female mice. For scRNA-seq experiments, drug-product pools were generated from male mice only. For both i.v. and i.c.v. administration, animals were dosed with 5 × 10^5^ cells. i.c.v. administration was performed using a stereotaxically guided Hamilton syringe and infusion system (Harvard Apparatus). The single infusion was performed unilaterally in the lateral ventricle in the following coordinates: AP = -0.5 mm (anterior from bregma); ML = +1.0 mm; DV = -1.75 mm. For animals dosed using both i.v. and i.c.v., animals were dosed with 5 × 10^5^ for both routes for a total of 1 × 10^6^ cells. Five days after drug-product administration, all animals despite route of administration received an additional i.v. administration of non-transduced total BM cells (5 × 10^5^ cells in 50 μL per animal, resuspended in DPBS without Ca^2+^ or Mg^2+^) to support the survival of animals that only received i.c.v. administration of drug product. Subsequent studies (GBA-PD and GRN-FTD proof-of-concept studies) only administered drug product i.v., and the second total BM administration was eliminated. Additionally, in these studies, donors and recipients were sex matched, and for the GRN-FTD proof-of-concept study, an additional analysis of 5 days of busulfan conditioning was added.

### Lentiviral vector generation

VSV-G pseudotyped third-generation self-inactivating lentiviral vectors for LVV.GFP, LVV.GRN, and LVV.GBA were commercially generated (University of Cincinnati Vector Production Facility, VIVEbiotech, VectorBuilder) using HEK293T cells and standard protocols. Lentiviral vector functional titers ranged from 1.7 × 10^8^ to 3 × 10^9^ TU/mL.

### Sample preparation and molecular characterization

Genomic DNA (gDNA) was isolated using the QIAamp DNA Mini QIAcube Kit (Qiagen) in conjunction with the QIAcube (Qiagen). Immortalized cell line gDNA was isolated using the DNeasy Blood & Tissue kit (Qiagen). VCN quantification was completed either by digital droplet PCR (ddPCR) or quantitative PCR (qPCR). Specific primers targeting the WPRE element (WPRE v1) or HIV Psi element were used to detect the integrated lentiviral vector, and specific primers targeting *Gtdc1* or *Tfrc* were used as genomic reference (Table S1).

RNA isolation and reverse transcription for murine samples from the GRN-FTD proof-of-concept study was completed using the QIAsymphony RNA kit (Qiagen). Absolute quantification of transgene copies per microgram of tissue was completed using a standard curve of *in*-*vitro*-transcribed WPRE RNA and specific primers and probes targeting WPRE (WPRE v2; Table S1). RNA isolation and reverse transcription for all other samples were completed using the PureLink RNA Mini kit (ThermoFisher) followed by SuperScript IV VILO with ezDNase (ThermoFisher). Specific primers and probes targeting the WPRE element (WPRE v1; Table S1) of the integrating lentiviral vector and *Actb* (ThermoFisher), as a housekeeper, were used. Primers and probes, cDNA, and TaqMan Fast Advanced Master Mix (ThermoFisher) were combined according to the manufacturer’s guidelines and amplified using a QuantStudio 7 Flex (ThermoFisher). The 2^-ΔΔCt^ method was used to calculate transgene expression.

Protein was extracted using a detergent-based lysis buffer dependent on the assay followed by removal of cellular debris by centrifugation at 14,000 × *g* for 15 min. Protein concentration was determined using the Pierce BCA Protein Assay Kit (ThermoFisher). SDS-PAGE was used to measure beta GCase and progranulin protein expression. GCase enzymatic activity was measured using the artificial substrate 4-methylumbelliferyl b-D-glucopyranoside (4-MUG) following a similar method to that previously published.^60^^,^^61^ Progranulin protein levels were measured using a human-specific ELISA (Quantikine and DuoSet; R&D Systems) according to the manufacturer’s instructions, with minor modifications for the measurement of human progranulin in brain lysates. For brain samples, reagents were diluted in 0.05% Tween 20 in Tris-buffered saline (TBS). Seventy-five μg of protein was loaded of mouse brain lysate and 1.5 μg of protein was loaded for mouse BM, and plasma was measured at a final dilution of 1:500 for the human progranulin ELISA assay. Thirty to 500 ng of protein was used to measure transgene expression of progranulin in RAW264.7 cells. Animal cohort sizes and sample numbers for all analysis shown are listed in Table S2.

### Data and materials availability

Transcriptomic data discussed in this publication have been deposited in NCBI's Gene Expression Omnibus (GEO) database and are available through GEO: GSE201756. Additional data may be made available from AVROBIO, Inc., upon request with the appropriate material transfer agreement. All other data are available in the main or the supplemental information.

Additional methods and materials are included within the supplemental information.

## Supplemental information

Document S1. Supplemental methods, Supplementary Table 1, and Supplementary Figures S1–S13

Table S2. Description of sample and cohort sizes for all experiments in main text

Document S2. Article plus supplemental information
