# Supplementary material for: High-throughput analysis of hematopoietic stem cell engraftment after intravenous and intracerebroventricular dosing
Source: Mol Ther. 2022 May 25;30(10):3209–25. doi: 10.1016/j.ymthe.2022.05.022 (PMC9552809; doi:10.1016/j.ymthe.2022.05.022)
Supplement: Document S1. Supplemental methods, Supplementary Table 1, and Supplementary Figures S1–S13 [file mmc1.pdf]

YMTHE, Volume *30*

Supplemental Information

High-throughput analysis of hematopoietic stem cell engraftment after intravenous and intracerebroventricular dosing

Robert N. Plasschaert, Mark P. DeAndrade, Fritz Hull, Quoc Nguyen, Tara Peterson, Aimin Yan, Mariana Loperfido, Cristina Baricordi, Luigi Barbarossa, John K. Yoon, Yildirim Dogan, Zeenath Unnisa, Jeffrey W. Schindler, Niek P. van Til, Luca Biasco, and Chris Mason

SUPPLEMENTARY METHODS

SDS-PAGE of beta glucocerebrosidase, progranulin, and Cathepsin D

For RAW264.7 cells, 2.5 µg of protein lysate was mixed with 4x protein sample buffer (XT Sample Buffer, Bio-Rad) and water to a volume of 15 µL. The samples were heated at 95°C for 5 minutes. Samples were loaded into precast 10% Bis-Tris polyacrylamide gels (Bio-Rad) with MOPS buffer (Bio-Rad) and electrophoresed. For brain lysates, 10 µg of protein lysate was mixed with 4x protein sample buffer (Invitrogen or Bio-Rad) and water or lysis buffer to a volume of 15 µL. The samples were heated at 70-95°C for 5-10 minutes. Samples were loaded into precast 4-20% Tris-HCl polyacrylamide gels (Bio-Rad) with Tris/Glycine/SDS buffer (Bio-Rad) and electrophoresed. After completion of electrophoresis, gels were transferred to PVDF membranes using the iBlot 2 system (ThermoFisher). PVDF membranes were blocked for 1 hour with 5% BSA in 0.05% PBS-Tween. Primary antibodies (GCase, progranulin, cathepsin D, beta-actin) were diluted in 5% BSA in 0.05% PBS-Tween and incubated with PVDF membranes overnight at 4°C. PVDF membranes were washed three times with 0.05% PBS-Tween and then incubated with appropriate HRP secondary antibodies (Bio-Rad) for 1 hour at 1:50,000 dilution in 5% BSA in 0.05% PBS-Tween. Blots were washed three times in 0.05% PBS-Tween for 10 minutes each and then incubated with chemiluminescence reagent (SuperSignal West Femto, ThermoFisher). Images were acquired using a charge-coupled camera (G:BOX, Syngene). Bands were quantified using ImageJ.

Transduction of immortalized cell lines

RAW264.7 mouse macrophage cells (ATCC; confirmed by STR profiling, IDEXX) were transduced with increasing multiplicity of infections with LVV.GRN or LVV.GBA vector. Approximately 85,000 cells per well of a 6-well dish were seeded 24 hours prior to transduction in 2 mL of complete growth media (DMEM supplemented with 10% heat-inactivated FBS, pyruvate, GlutaMAX, and penicillin/streptomycin). Transduction media was removed after 16 hours and replaced with fresh complete growth media. Cells were collected 4 days post-transduction, washed with DPBS twice, pelleted by centrifugation at 500xg for 5 min, and frozen at -80°C prior to downstream molecular characterization.

Flow cytometry analysis for myeloid and lymphoid populations

For the blood and bone marrow samples, red blood cells were lysed and resuspended in RPMI medium. White blood cells were split into aliquots. The first aliquot was stained with CD45.1-APC (clone A20), CD45.2-PE (clone 104), CD3ε-PerCP-Cy5.5 (clone 17A2), and B220-APC-Cy7 (clone RA3-6B2) for 20 minutes at 4°C. The second aliquot was stained with CD45.1-APC (clone RB6-8C5), CD45.2-PE (clone 104), TER119-PerCP-Cy5.5, CD11b-PE-Cy7 (M1/70), GR1-APC-Cy7 (clone RB6-8C5) for 20 minutes at 4°C. After two washes, both aliquots were incubated with the viability marker Sytox Blue for 8 minutes at room temperature. Stained cells were measured using a MACSQuant Analyzer 10 (Miltenyi Biotec) and analyzed using FlowJo (FlowJo, LLC).

Quantification of GFP and Iba1 positive cells in the brain

Five sections across the rostral caudal axis of the brain (two sections from approximately +0.2 mm to +3.2 mm relative to bregma, two sections from approximately +0.2 mm to -4.06 mm relative to bregma, and one section covering the cerebellum from approximately -5.2 mm to -6.1 mm to bregma) were uploaded to the HALO platform (Indica Labs, version 3.1.1076.301) for analysis. A tissue classifying algorithm was trained to detect and select tissue from background, thereby creating a region of interest for subsequent analysis. Analysis outputs for each image set included positive signal area for both stains, colocalized positive signal area, cell count for each stain, and colocalized cell count.

Isolation of microglia/MLCs and single cell RNA-Seq

After transcardial perfusion, the brains of one male and female mice from IV and ICV groups were processed by enzymatic digestion (Neural Tissue Dissociation Kit (P), Miltenyi Biotec). The resulting single cell suspension was stained with viability dye and antibodies for microglia cells, neurons, astrocytes and endothelial cells (LIVE/DEAD Fixable Aqua Dead Cell Stain Kit, ThermoFisher; CD45.1 clone A20 in BV421 (BD); CD45.2 clone 104 in BV421 (BD); CD11b clone M1/70 in APC-780 (ThermoFisher); CX3CR1 clone SA011F11 in BV605 (BioLegend); PECy7 Thy1.1 (also known as CD90.1) clone OX-7 in PE-Cy7 (BD); Thy1.2 (also known as CD90.2) clone 53-2.1 in PE-Cy7 (BD);

ACSA-2 clone IH3-18A3 in PE (Miltenyi Biotec); CD31 clone 390 in PerCP/Cy5.5 (BioLegend). GFP-positve and GFP-negative fractions of microglia cells, defined as CD45+ CD11b+ Cx3cr1+, were FACS-sorted using a MA900 Multi-Application Cell Sorter (Sony Biotechnology). For the generation of single cell transcriptomes, $5x10^3$ target cells from each sorted population were run using the Chromium Controller (10x Genomics) using Chromium Next GEM Single Cell 3' Reagent Kits (10x Genomics). The libraries generated were then run on the NextSeq 550 Sequencing System (Illumina) using NextSeq 500/550 High Output v2.5 (150 cycles) Kit (Illumina). The Illumina raw BCL sequencing files were processed through the CellRanger software (10x Genomics) for generating FASTQ files and count matrixes (https://support.10xgenomics.com/single-cell-gene-expression/software/overview/welcome). The count matrixes were then used as input for the SEURAT V4.0 (https://satijalab.org/seurat/) and MONOCLE3 (http://cole-trapnell-lab.github.io/monocle-release/) R tools for single cell genomics analyses. Briefly, single cell barcodes were filtered for the ones containing mitochondrial gene content lower than 15%. Expression data then were normalized, scaled, and searched for variable features using the *SCTransform* function of SEURAT V4.0 followed by tSNE dimensionality reduction and clustering using the *FindClusters* function with resolution set at 0.2. For generating the maps shown in Fig.3b and Fig.4a we run the tSNE coordinates on the R package *ggplot2* (https://cran.r-project.org/web/packages/ggplot2/index.html). For visualization of gene expression shown in Fig.2b the SEURAT object was converted into a MONOCLE object and individual genes were plotted using the *plot_cells* function of MONOCLE3. The heatmaps of Fig. 3e, 4d and 5a were generated based on the average normalized gene expression per sample using the R package pheatmap (https://cran.r-project.org/web/packages/pheatmap/index.html).

Statistics

Statistical analysis was performed using either Prism (GraphPad) or R. For analyzing the relationship between vector volume and transgene metrics in Fig.6, we fit a linear regression model and measured the Pearson correlation and its statistical relevance (p-value). For scRNA-Seq data we used GSEAPreranked analysis to identify the gene sets that are enriched in GFP-negative and GFP-positive

samples. To perform this analysis, we firstly derived a score using log2FoldChange and p-value from differential gene expression analysis (score=-s*log10(p-value) with s=-1 if the log2FoldChange of gene <0, otherwise s=+1). We then sorted the genes based on the score value in decreasing order and used the whole ranked gene list file (including 11,992 genes and their score values) as input for the online tool WebGestalt (http://www.webgestalt.org) to perform GSEAPreranked analysis. We then generated a network-based enrichment map visualization for the 20 enriched gene sets. In this network, the edges correspond to the overlapping between connected gene sets and were calculated using the gene list of the leading-edge genes of each gene set from GSEA. The node size instead indicates the number of the leading-edge genes. The overlapping score was calculated using the following formula: $overlap = \frac{|A \cap B|}{\min(|A|,|B|)}$ where $|A \cap B|$ is the cardinality of the intersection between gene sets A and B while $|A|$ and $|B|$ are the cardinality of gene set A and gene set B, respectively. For the plot shown in Fig. 3D we applied the Procrustes tool from the R Package Vegan (https://cran.r-project.org/web/packages/vegan/vegan.pdf) to perform unsupervised clustering on the global gene*cell expression matrixes in different sample types. Since the number of cells for sample types are different, we downsampled all sample types to the smallest dataset and then calculated Procrustes distance for each sampled data sets and calculated and plotted the average from 10 random samplings. We then perform clustering using the averaged Procrustes distance. For all other data, a Student t-test (when only two groups were present), one-way ANOVA with Tukey's post hoc analysis (when multiple groups were present), or linear regression was used. Significance was defined as $p < 0.05$.

Immunofluorescence staining

For quantification of engraftment within the central nervous system, brains were paraffin embedded, sectioned at 20 μm, and 12 sections were stained for GFP and Iba1 and 12 sections were stained for GFP and TMEM119 at a 300 μm interval. Slides were scanned using VS200 Research Slide Scanner (Olympus). Images were uploaded and analyzed using the HALO platform (v3.1.1076.301, Indica Labs).

For qualitative analysis of GFP engraftment, peripheral tissues were fixed overnight in 4% PFA and then transferred to a solution of 30% sucrose until they sank to the bottom. Subsequently, tissues were mounted in tissue freezing media and stored at -80°C. Tissues were sectioned at 20-50 μm (depending on tissue) using a sliding microtome with a frozen stage (ThermoFisher) or cryostat (Leica). Sections were mounted on glass slides using a glycerol-based mounting media.

For progranulin IHC, brains were processed similar to peripheral tissues. Sections were first incubated with an antigen retrieval solution (Antigen Retrieval Reagent-Basic, R&D Systems) at 80°C for 30 minutes followed by blocking (2% non-fat dry milk, 0.3% Triton X-100, and 0.01% sodium azide in PBS). Next, sections were blocked again in 1% donkey serum in 0.3% Triton X-100 in PBS for 1hr at room temperature. Sections were then incubated with primary antibodies for progranulin (AF2420 R&D Systems) and NeuN (MAB377 MilliporeSigma) overnight at 4°C followed by the appropriate fluorescent secondary antibodies for one hour.

Supplemental Table 1. Primers and probe sequences

Target	Purpose	Primer	Sequence (5'-3')	Vendor
WPRE (v1)	VCN, Expression	Forward	TTCTGGGACTTTCGCTTTCC	IDT
WPRE (v1)	VCN, Expression	Reverse	CCGACAACACCACGGAATTA	IDT
WPRE (v1)	VCN, Expression	Probe	ATCGCCACGGCAGAACTCATCG	IDT
Tfrc	VCN	Forward	Not available	ThermoFisher, 4458367
Tfrc	VCN	Reverse	Not available	ThermoFisher, 4458367
Tfrc	VCN	Probe	Not available	ThermoFisher, 4458367
Gtdc1	VCN	Forward	GAAGTTCAGGTTAATTAGCTGCTG	IDT
Gtdc1	VCN	Reverse	GGCACCTTAACATTTGGTTCTG	IDT
Gtdc1	VCN	Probe	ACGAACTTCTTGGAGTTGTTTGCT	IDT
Actb	Expression	Forward	Not available	ThermoFisher, Mm02619580_g1
Actb	Expression	Reverse	Not available	ThermoFisher, Mm02619580_g1
Actb	Expression	Probe	Not available	ThermoFisher, Mm02619580_g1
WPRE (v2)	Expression	Forward	GGCTTTCRTTTTCTCCTCCTTGTAT	ThermoFisher
WPRE (v2)	Expression	Reverse	CGGGCCACAACTCCTCATAA	ThermoFisher
WPRE (v2)	Expression	Probe	AATCCTGGTTGCTGTCTC	ThermoFisher
Psi element	VCN	Forward	CAGGACTCGGCTTGCTGAAG	IDT
Psi element	VCN	Reverse	TCCCCCGCTTAATACTGACG	IDT
Psi element	VCN	Probe	CGCACGGCAAGAGGCGAGG	IDT

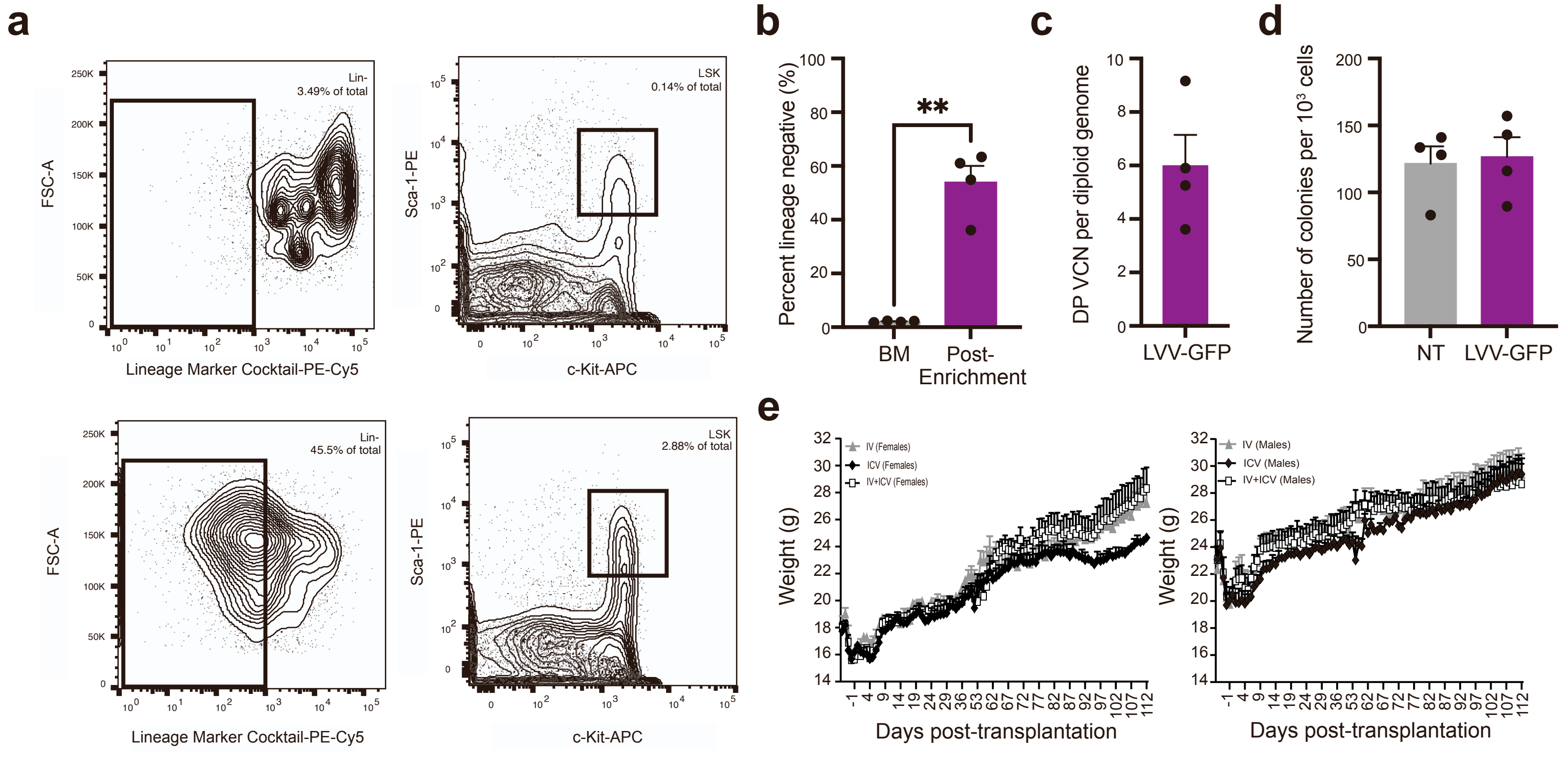

Supplementary Figure 1. Drug product characterization and post-transplantation body weights.
a Representative flow cytometry analysis plots of total bone marrow (top) and lineage negative cells after enrichment (bottom) for lineage markers (left panels) and c-Kit/Sca1 (right panels). **b** Quantification of lineage negative population in four drug product preprarations. **c** Vector copy number per diploid genome in the drug product. **d** Colony forming unit assay to assess pluripotency of lineage negative cells. **e** Body weights of female (left) and male (right) animals through the course of the study. LSK=Lineage negative, Sca1+, c-Kit+. T-test was used for statistical analysis. **, p<0.01. Bars represent means ± SEM.

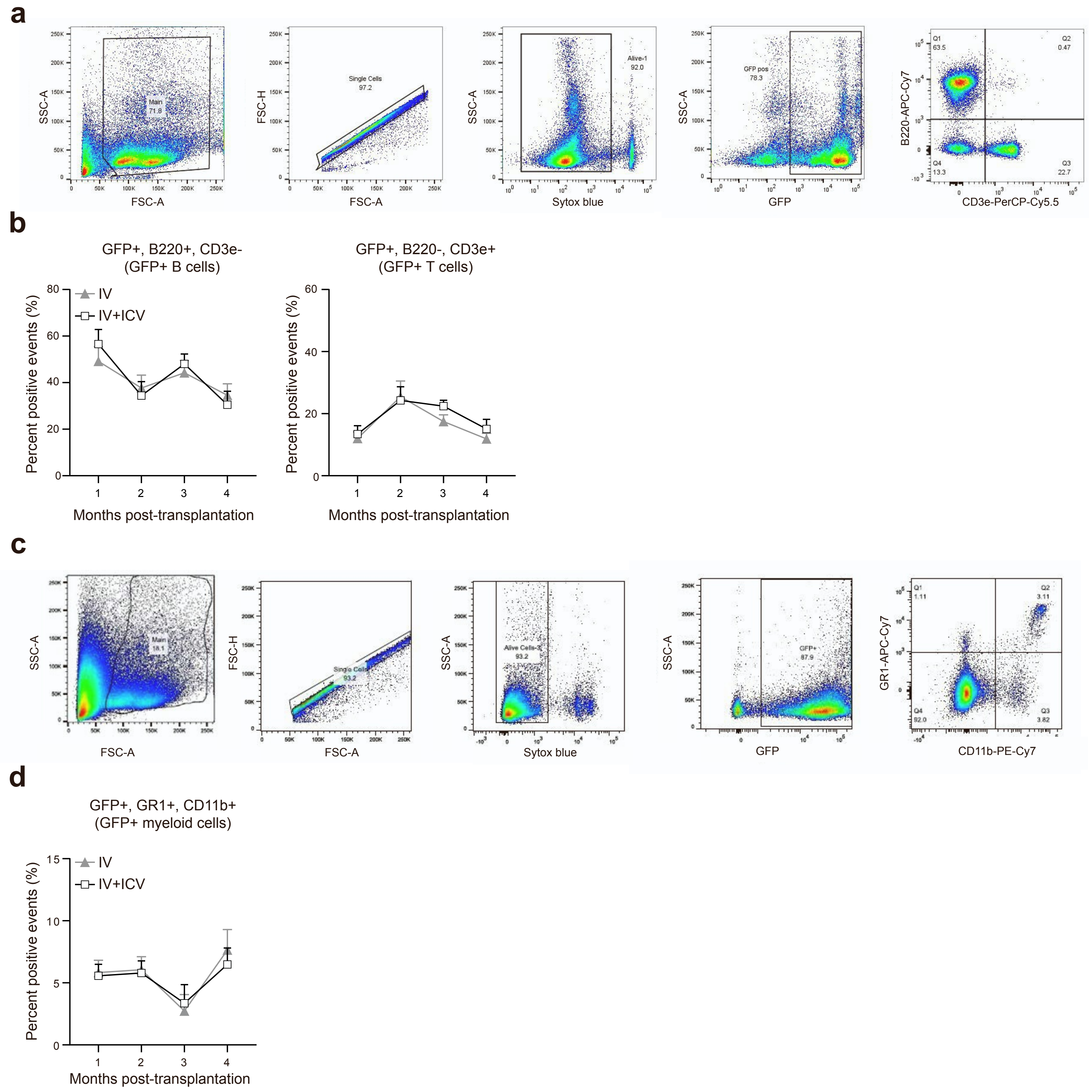

Supplementary Figure 2. Flow cytometry analysis of peripheral blood to measure engraftment in various cell compartments. a-b Representative flow cytometry plots and quantitation of GFP-positive (GFP+) B cells (B220+, CD3e-) and T cells (B220-, CD3e+) in IV and IV+ICV animals. **c-d** Representative flow cytometry plots and quantitation of GFP-positive myeloid cells (GR1+, CD11b+) in IV and IV+ICV animals. Symbols represent means ± SEM.

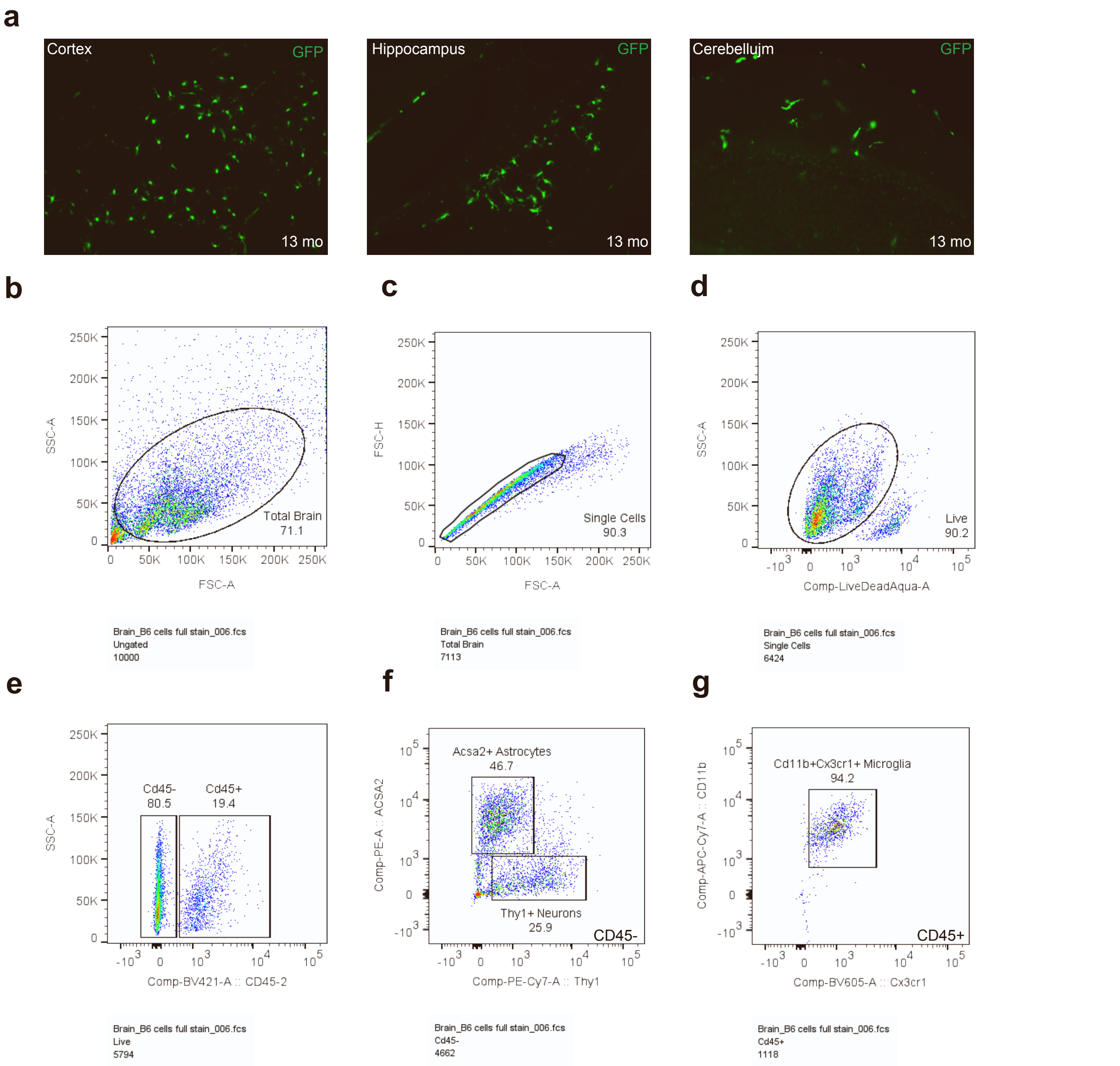

Supplementary Figure 3. Representative images of engraftment at 13 months post-treatment and gating strategy for FACS isolation of microglia, astrocytes, and neurons. (a) Representative images of GFP-positive cells in the cortex, hippocampus, and cerebellum at 13 months post-treatment. Whole adult mouse brains were enzymatically digested to single cell suspension and then stained with cell-surface marker antibodies to enable FACS-assisted isolation of specific cell populations. Representative gating strategy shows the selection of the single cell population **(b,c)**, selection for viability **(d)**, gating on the immune cell marker CD45 **(e)** selection of CD45- ACSA2+, Thy1- astrocytes, and CD45- ACSA2- Thy1+ neurons **(f)**, selection of CD45+ CD11b+, C3X3CR1+ microglia **(g)**. Percentages of parent population are listed next to each gate.

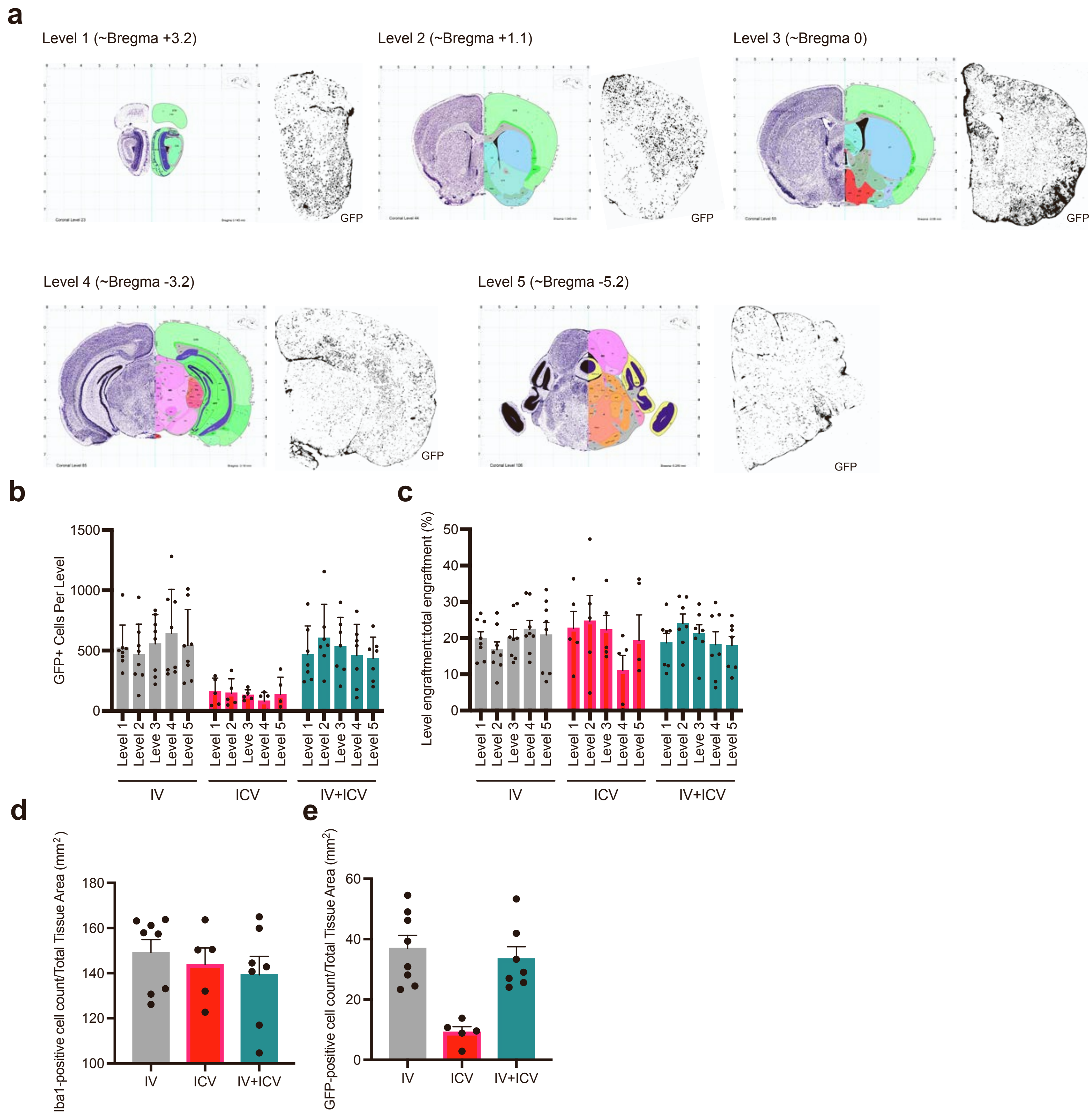

Supplementary Figure 4. Engraftment distribution and density in the brain. (a) Representative reference atlas images (from the Allen Mouse Brain Atlas and Allen Reference Atlas - Mouse Brain) and sudy images of GFP+ cells (threshold applied in ImageJ to remove background) for each level analyzed. (b) Number of GFP+ cells counted for each level analyzed. (c) Number of GFP+ cells for each level normalized to the total number of GFP+ cells found in the brain. (d) Number of Iba1+ cells per mm^2. (e) Number of GFP+ cells per mm^2.

Allen Institute for Brain Science (2004). Allen Mouse Brain Atlas [dataset]. Available from mouse.brain-map.org.Allen Institute for Brain Science (2011).
Allen Reference Atlas – Mouse Brain [brain atlas]. Available from atlas.brain-map.org.

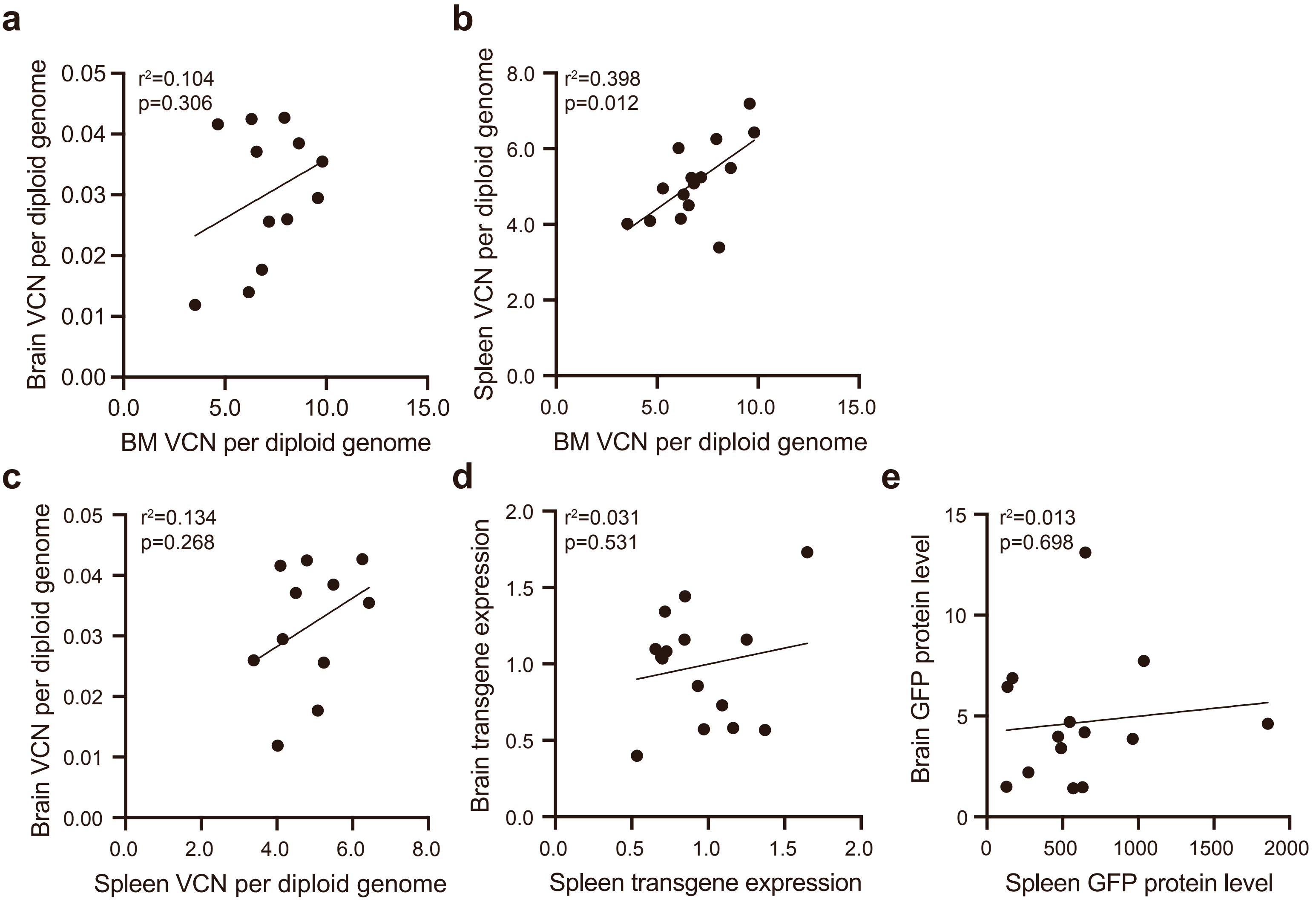

Supplementary Figure 5. Linear regression analysis of brain, bone marrow, and spleen biodistribution metrics from animals dosed IV or IV+ICV.

a Brain vector copy number (VCN) versus bone marrow (BM) VCN per diploid genome. **b** Spleen VCN versus BM VCN. **c** Brain VCN versus spleen VCN. **d** Brain transgene expression vs spleen transgene expression**. e** Brain GFP protein versus spleen GFP protein. Line and statistics represent linear fit after simple linear regression.

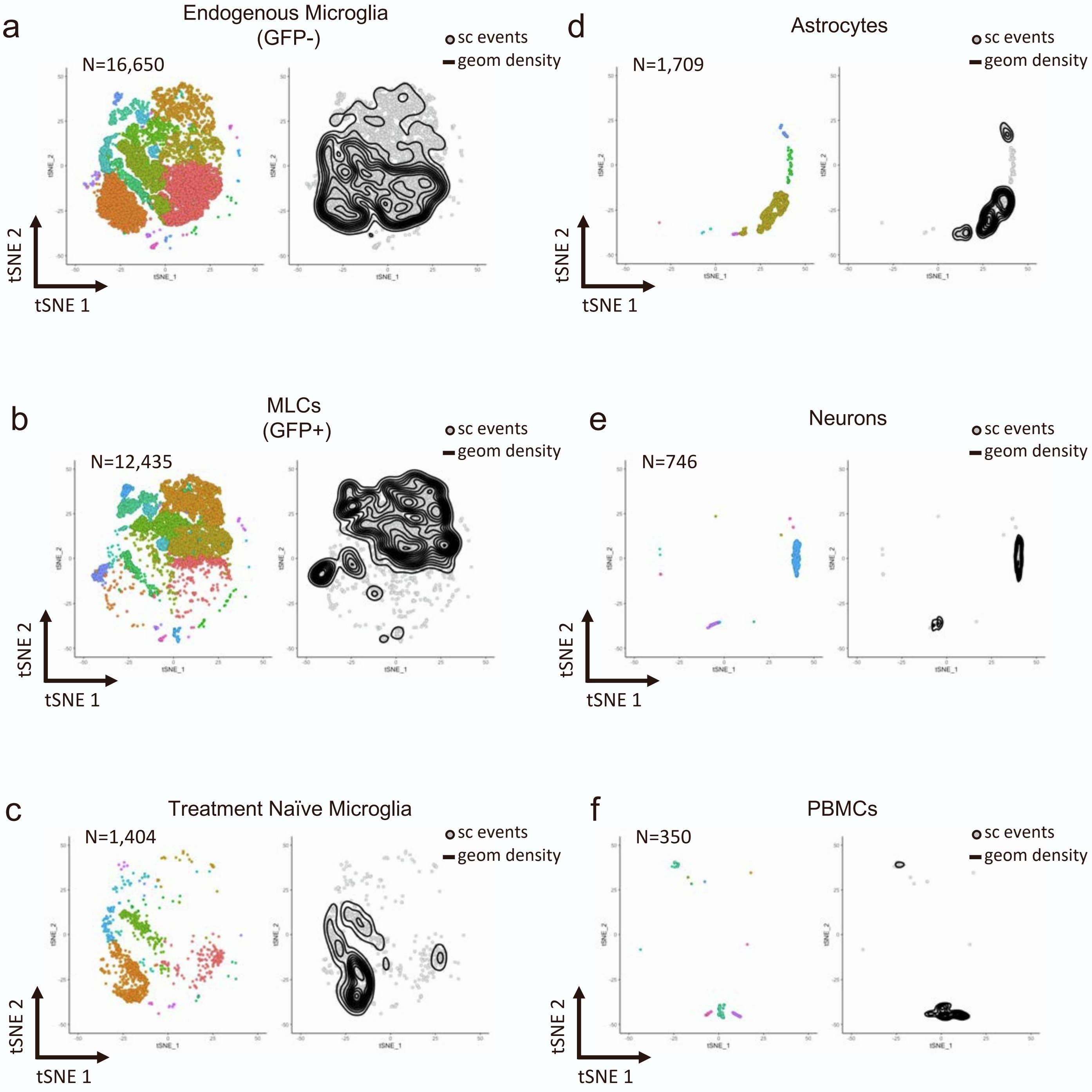

Supplemental Figure 6. tSNE plots for combined analysis from Figure 3 separated by sample
Shown are tSNE plots from the analysis shown in figure 3 separated by sample type: endogenous microglia (**a**), MLCs (**b**), treatment naïve microglia (**c**), astrocytes (**d**), neurons (**e**) and PBMCs (**f**).

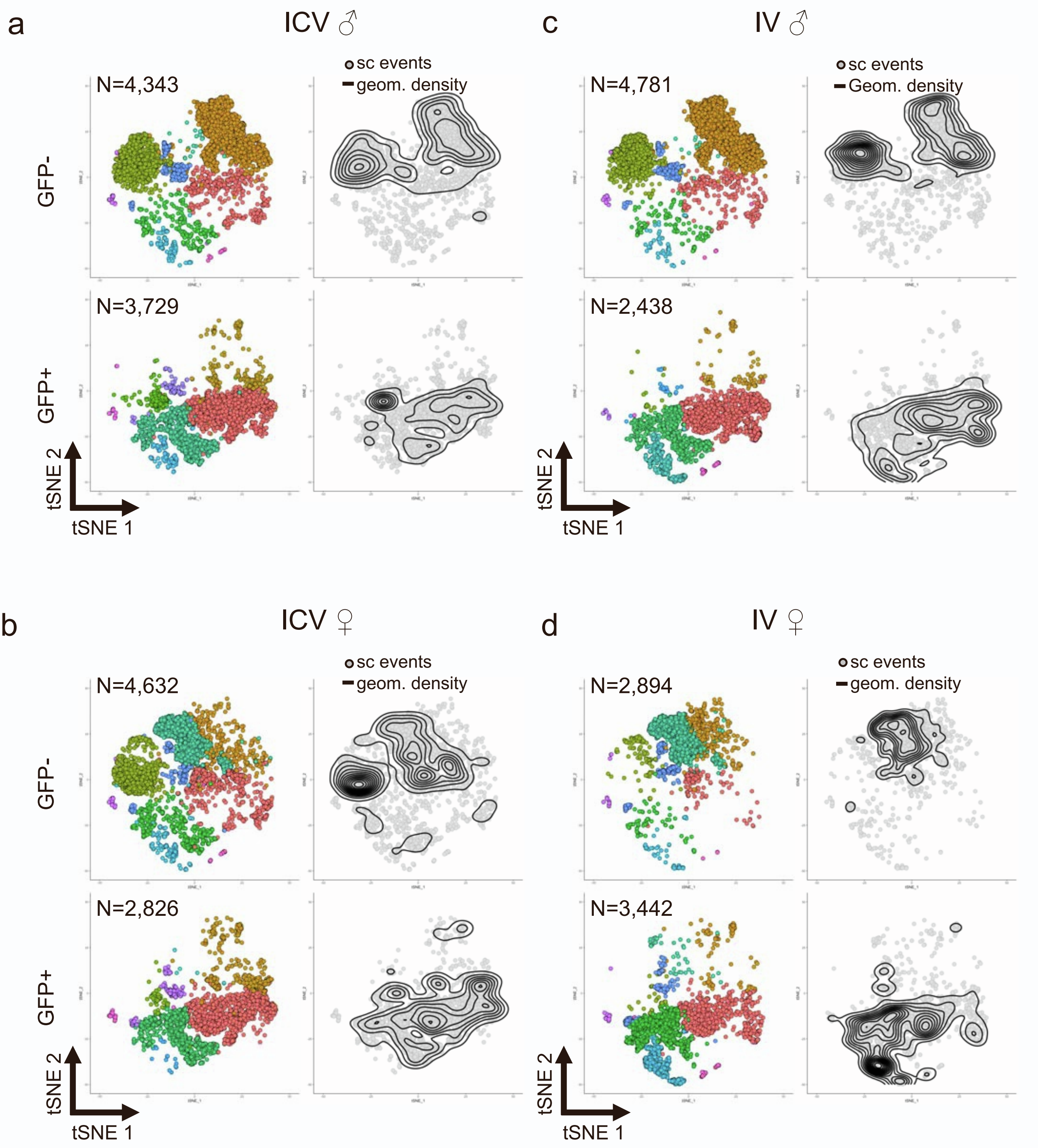

Supplemental Figure 7. tSNE plots for combined analysis from Figure 4 separated by sample type
Shown are tSNE plots from the analysis shown in figure 4 separated by animal. The GFP+ MLC and GFP- microglia samples are shown for the male (**a**) and female (**b**) dosed via ICV and for the male (**c**) and female (**d**) dosed via IV.

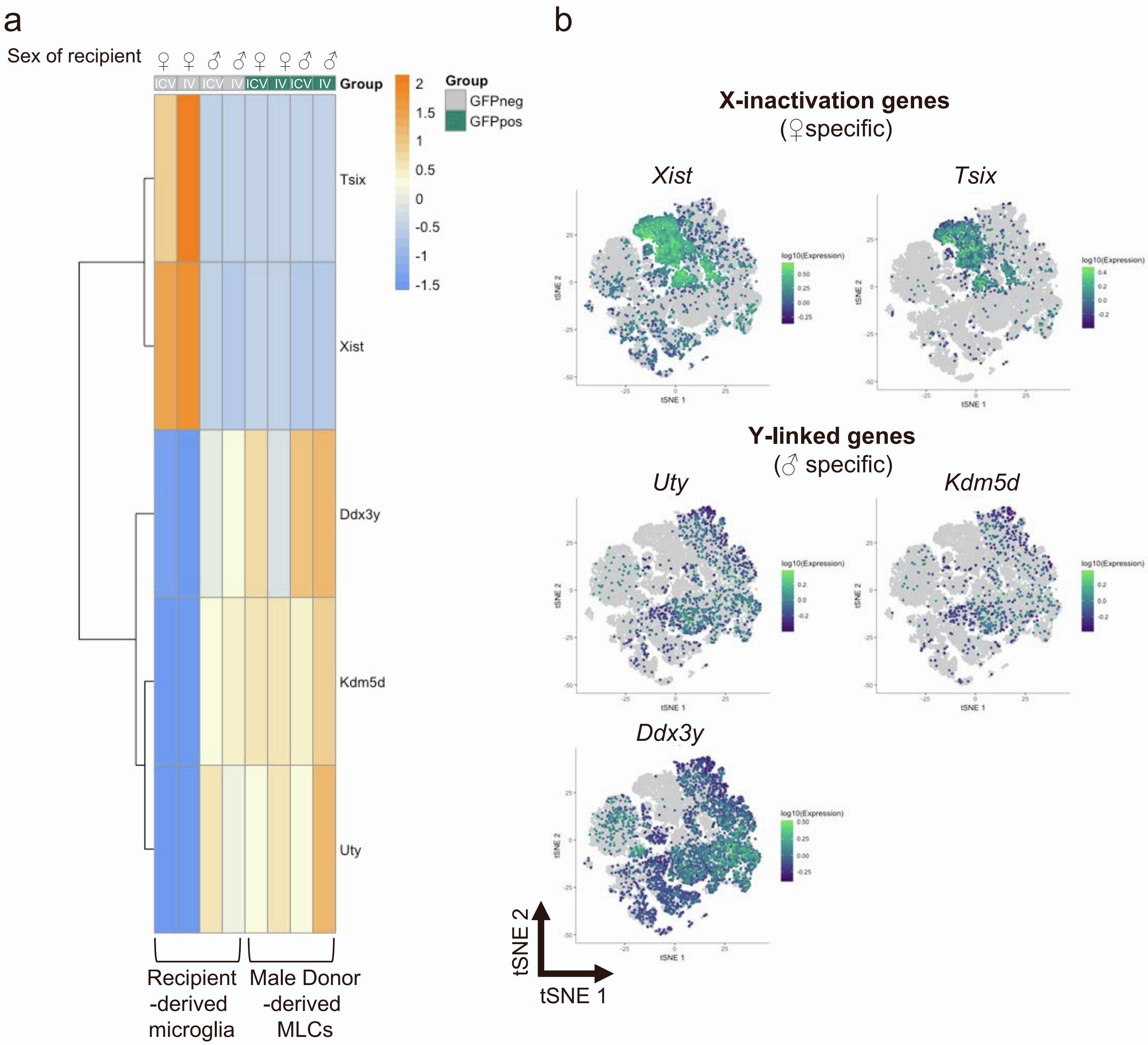

Supplemental Figure 8. Expression of sex-chromosome genes in MLCs and microglia.
a Heat maps of normalized expression for two X-linked genes associated with X-inactivation (*Xist*, *Tsix)* and three Y-linked genes (*Ddx3y*, *Uty*, *Kdm5d)* separated by sample type*.* **b** tSNE plot colored for expression of the X-linked and Y-linked genes.

a

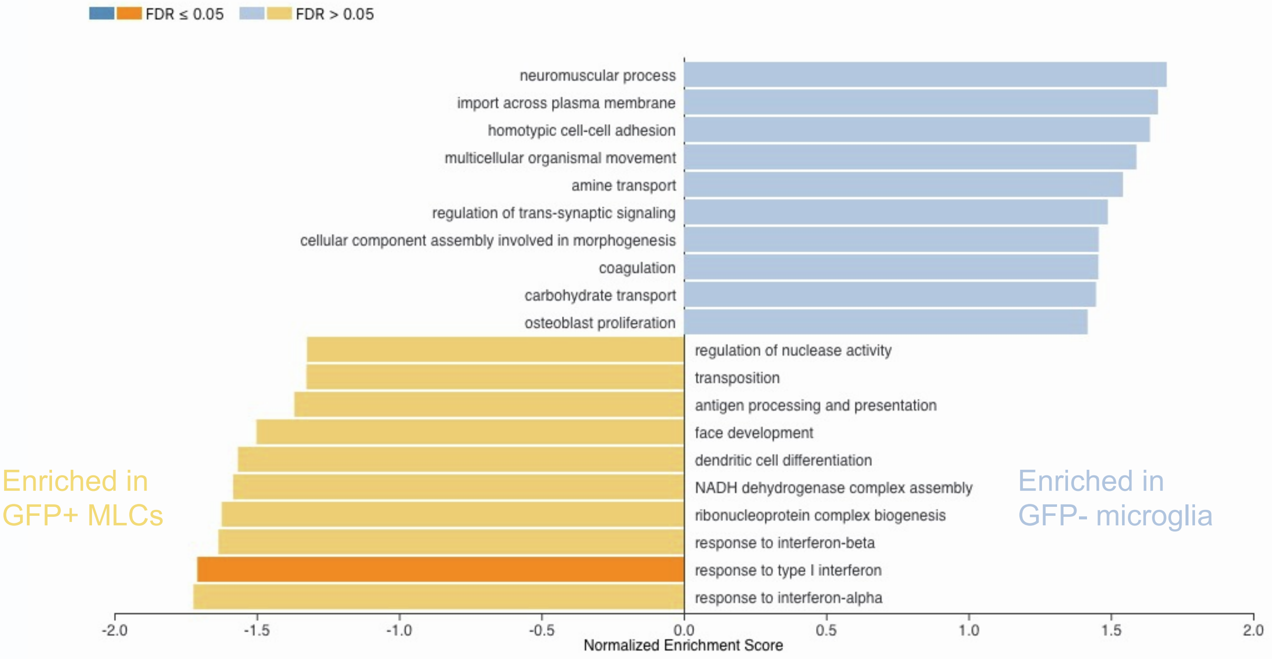

b

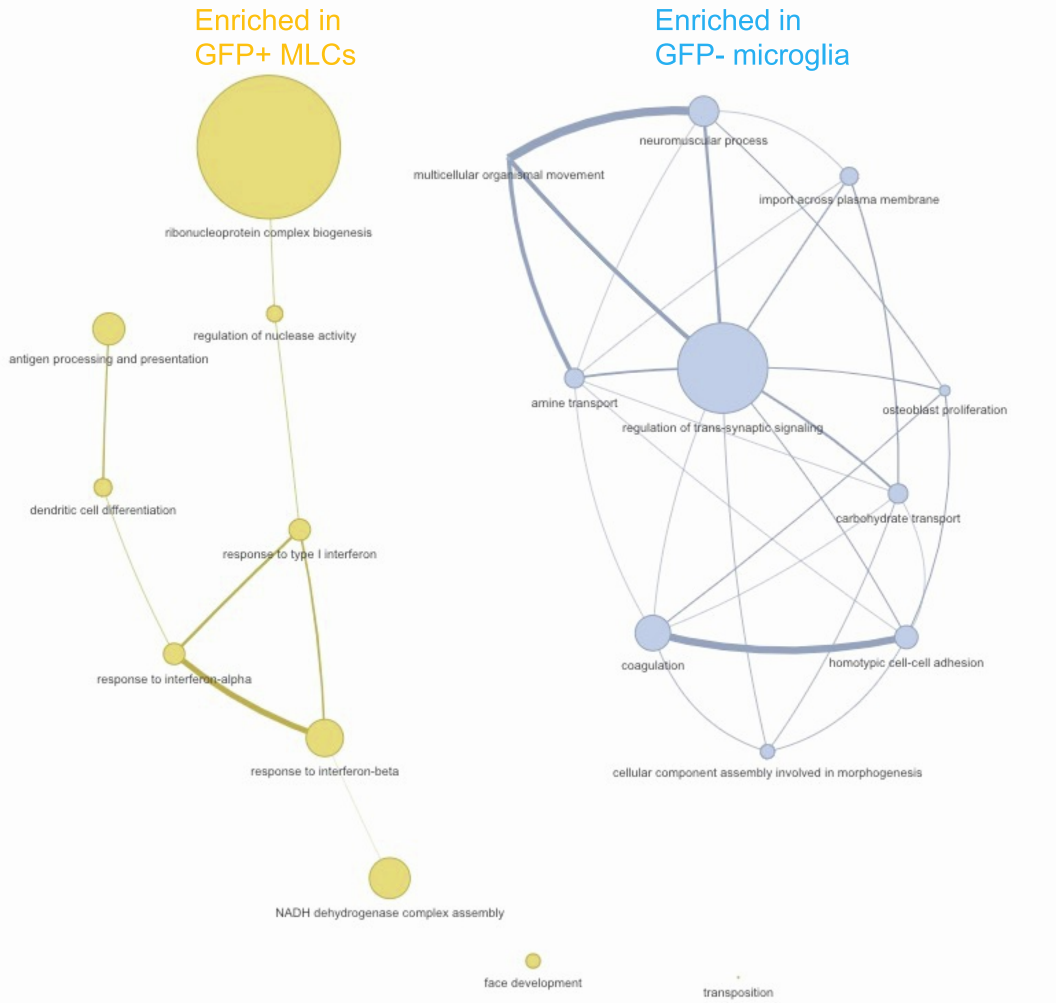

Supplemental Figure 9. Gene Ontology analysis comparing MLCs to microglia. a Top ten gene ontology terms associated with genes enriched in MLCS vs. endogenous microglia. GSEAPreranked analysis via webgestalt was used to generate GO term list. **b** Network-based enrichment map from GO terms is constructed by a R script using visNetwork R package.

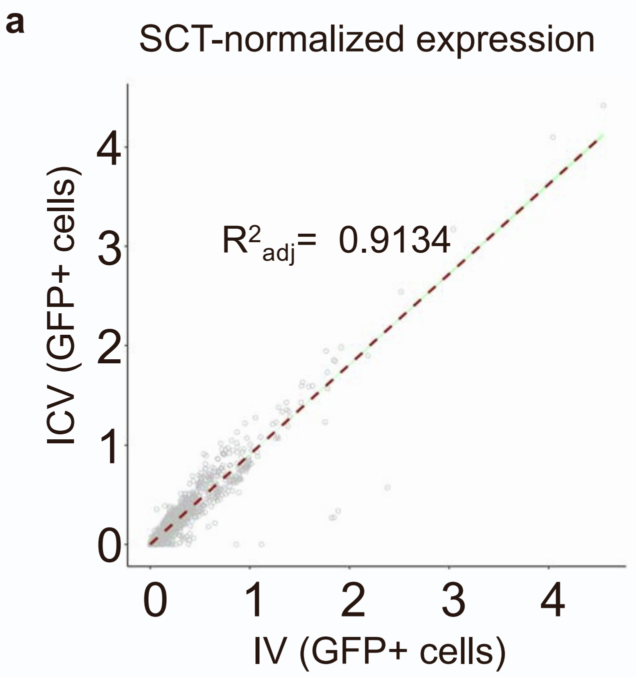

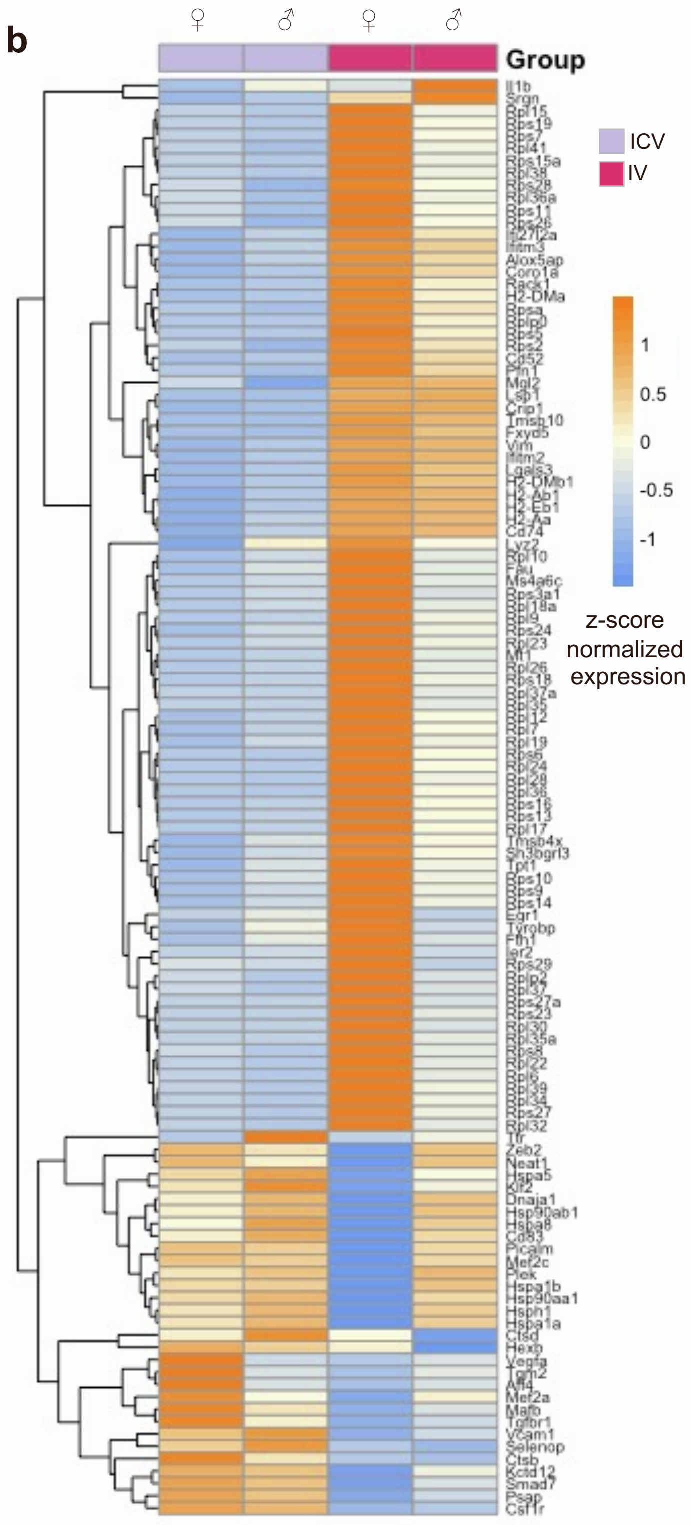

Supplementary Figure 10. Comparison of IV and ICV-derived MLCs. a Correlation analysis of MLCs isolated from IV and ICV dosed animals based on normalized single cell gene expression data. **b** Unsupervised clustering based on the global expression profile of each sample type (top). Heatmap showing top differentially expressed genes for each sample type.

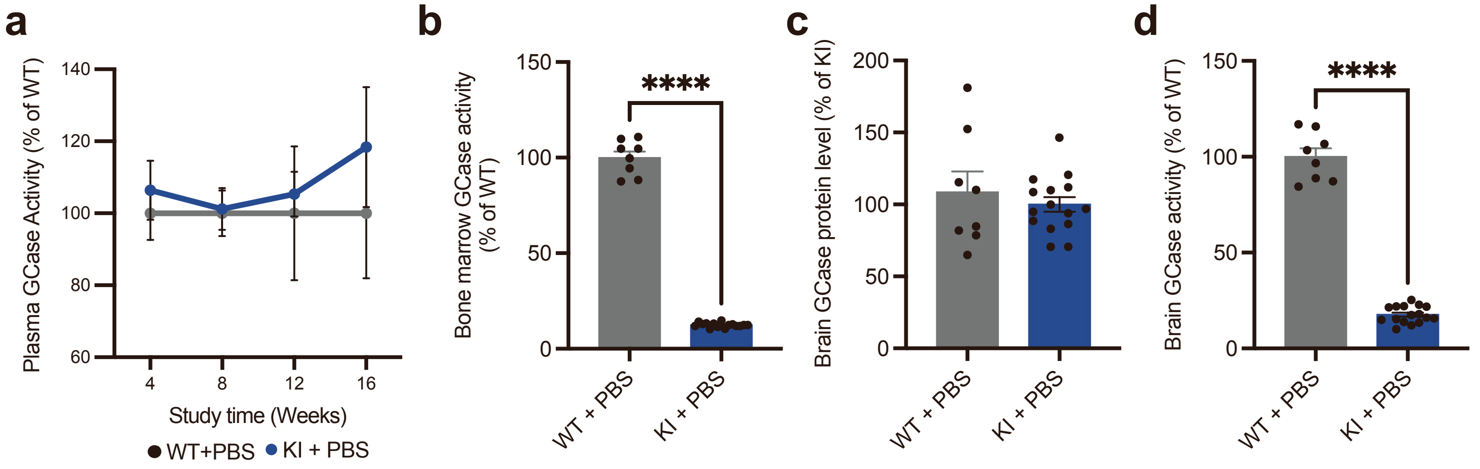

Supplementary Figure 11. Baseline characterization of homozygous Gba D409V knock-in mouse model. **a** GCase activity was measured from plasma at four week intervals through the course of the study and normalized to wild-type levels. Lack of difference between wild-type and knock-in animals is likely due to low endogenous activity and background noise. **b** GCase activity (normalized to wild-type animals) at the terminal timepoint of 16-weeks in the bone marrow. **c-d** GCase protein levels (normalized to knock-in animals) and activity (normalized to wild-type animals) were measured in the brain at the terminal timepoint of 16-weeks. T-test was used for statistical analysis. **, $p<0.01$. Bars represent means ± SEM.

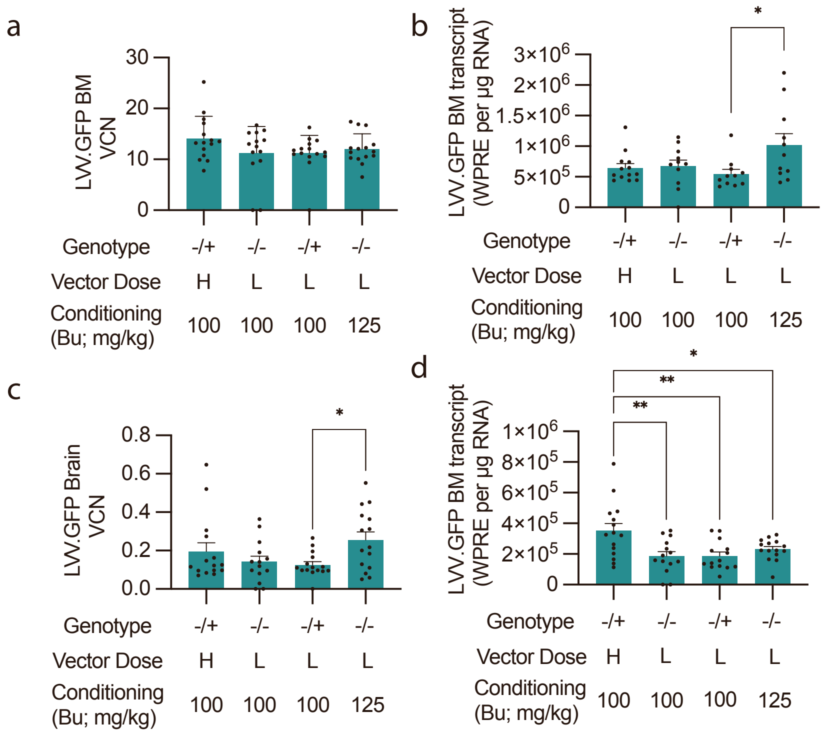

Supplementary Figure 12. VCN and WPRE quantification for LVV.GFP-treated animals at 16 weeks post-transplantation a Bone marrow VCN **b** Bone marrow WPRE **c** Brain VCN **d** Brain WPRE**.** All error bars are SEM. Tukey's multiple post-hoc comparison test was conducted for all statistical measures shown, with only significant differences indicated.

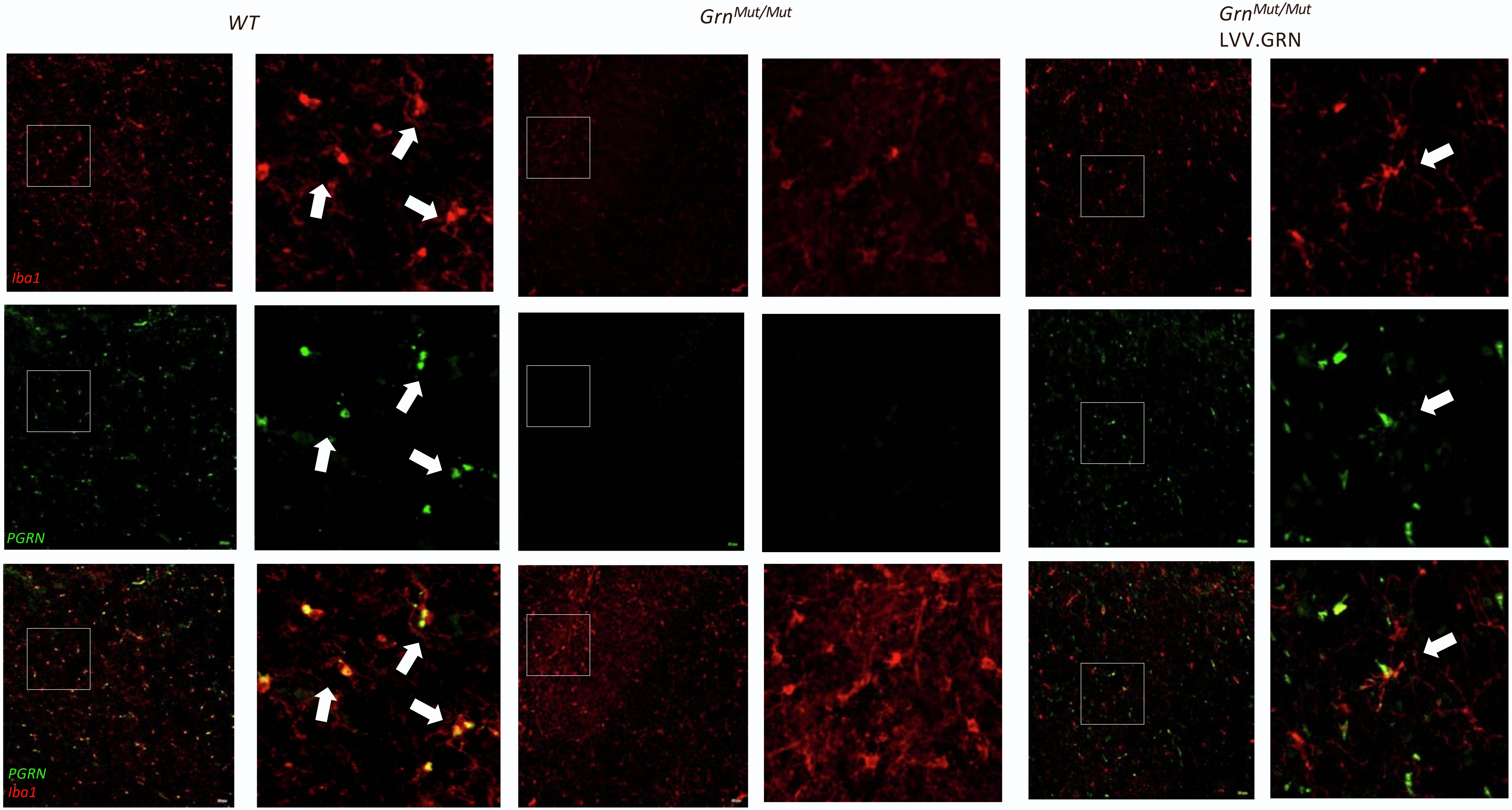

Supplementary Figure 13. Representative images of progranulin and Iba1 staining in WT, *Grn* mutant mice, and treated *Grn* mutant mice. Lack of staining in naïve *Grn* mutant animals demonstrates the specificity of the progranulin antibody. Staining is restored in *Grn* mutant mice after treatment. White boxes on the left image reflect the magnified image on the right. White arrows signify positive progranulin staining.
