# Supplementary material for: High-throughput analysis of hematopoietic stem cell engraftment after intravenous and intracerebroventricular dosing
Source: Mol Ther. 2022 May 25;30(10):3209–25. doi: 10.1016/j.ymthe.2022.05.022 (PMC9552809; doi:10.1016/j.ymthe.2022.05.022)
Supplement: Document S2. Article plus supplemental information [file mmc3.pdf]

Molecular Therapy
Original Article

High-throughput analysis of hematopoietic stem cell engraftment after intravenous and intracerebroventricular dosing

Robert N. Plasschaert,[1,5] Mark P. DeAndrade,[1,5] Fritz Hull,[1] Quoc Nguyen,[1] Tara Peterson,[1] Aimin Yan,[1] Mariana Loperfido,[1] Cristina Baricordi,[1] Luigi Barbarossa,[1] John K. Yoon,[1] Yildirim Dogan,[1] Zeenath Unnisa,[1] Jeffrey W. Schindler,[1] Niek P. van Til,[1,2] Luca Biasco,[1,3,6] and Chris Mason[1,4,6]

[1]AVROBIO, Inc, Cambridge, MA 02139, USA; [2]Department of Child Neurology, Amsterdam Leukodystrophy Center, Emma Children's Hospital, Amsterdam University Medical Centers, VU University, and Amsterdam Neuroscience, Amsterdam, the Netherlands; [3]Great Ormond Street Institute of Child Health, University College London, London, UK; [4]Advanced Centre for Biochemical Engineering, University College London, London, UK

Hematopoietic stem/progenitor cell gene therapy (HSPC-GT) has shown clear neurological benefit in rare diseases, which is achieved through the engraftment of genetically modified microglia-like cells (MLCs) in the brain. Still, the engraftment dynamics and the nature of engineered MLCs, as well as their potential use in common neurogenerative diseases, have remained largely unexplored. Here, we comprehensively characterized how different routes of administration affect the biodistribution of genetically engineered MLCs and other HSPC derivatives in mice. We generated a high-resolution single-cell transcriptional map of MLCs and discovered that they could clearly be distinguished from macrophages as well as from resident microglia by the expression of a specific gene signature that is reflective of their HSPC ontogeny and irrespective of their long-term engraftment history. Lastly, using murine models of Parkinson's disease and frontotemporal dementia, we demonstrated that MLCs can deliver therapeutically relevant levels of transgenic protein to the brain, thereby opening avenues for the clinical translation of HSPC-GT to the treatment of major neurological diseases.

INTRODUCTION

Hematopoietic stem/progenitor cell gene therapy (HSPC-GT) is a well-established paradigm for treating monogenic diseases, with more than 300 patients currently dosed.[1–4] Clinical applications of HSPC-GT include treatments of inherited immune deficiencies (e.g., X-linked severe combined immunodeficiency [SCID-X1], adenosine deaminase [ADA]-SCID, Wiskott-Aldrich syndrome), blood disorders (e.g., sickle cell disease, transfusion-dependent beta-thalassemia, X-linked chronic granulomatous disease), and lysosomal storage disorders (e.g., Hurler syndrome, Hunter syndrome, Fabry disease).[5–13] In general, a patient's bone marrow cells are mobilized and collected from the periphery, and HSPCs are isolated and transduced *ex vivo* with a lentiviral vector carrying a therapeutic payload. The genetically engineered cells are then intravenously infused back into the patient after a conditioning regimen using a myeloablative agent, such as busulfan. In mice, non-human primates, and humans, gene-modified HSPCs can engraft long term throughout the body, including within the hematopoietic compartment as cells of the bone marrow and blood, within the peripheral organs as tissue-resident macrophages, and within the brain and spinal cord as microglia-like cells (MLCs).[14–17]

The migration of genetically engineered cells across the blood-brain barrier and their engraftment within the brain parenchyma is key to HSPC-GT's ability to address neurological dysfunction. The conditioning agent regimen is thought to cause a partial depletion of microglia that can be repopulated by genetically modified MLCs that produce a therapeutic protein.[18] These MLCs can secrete this therapeutic protein and thereby provide a local source for uptake by neighboring cells, such as neurons, astrocytes, and oligodendrocytes. Late-stage HSPC-GT clinical trials for metachromatic leukodystrophy (MLD) and cerebral adrenoleukodystrophy (CALD) have demonstrated halting of expected CNS-related disease progression, with most patients being free of severe motor and cognitive dysfunction.[19–21] The fact that HSPC-GT can address the neurological symptoms of rare genetic diseases supports its possible translation to more common neurodegenerative disorders, such as Parkinson's disease and dementia.

Still, a wider application of HSPC-GT to neurological diseases requires a deeper understanding of the nature and dynamics of genetically engineered MLCs and their engraftment in the brain. Unknowns include the extent and durability of MLC engraftment within the CNS, the effect of different routes of HSPC administration

Received 12 November 2021; accepted 21 May 2022;
https://doi.org/10.1016/j.ymthe.2022.05.022.

[5]These authors contributed equally

[6]These authors contributed equally

Correspondence: Chris Mason, Advanced Centre for Biochemical Engineering, University College London, London, UK.
E-mail: chris.mason@ucl.ac.uk

This is an open access article under the CC BY license (http://creativecommons.org/licenses/by/4.0/).

on MLCs, and the comparability of gene-modified MLCs to endogenous microglia. In the present study, we have addressed these key questions in preclinical murine models of HSPC-GT and demonstrated that this platform can provide expression of a transgene at potentially therapeutic levels throughout the periphery and the brain in two mouse models of neurodegenerative disease.

RESULTS

Biodistribution of HSPC-GT-derived cells after intravenous (i.v.) and intracerebroventricular (i.c.v.) administration

Works in murine disease models have shown evidence of engraftment and functional rescue in the periphery and the brain after HSPC-GT using i.v. administration.[22–28] Additionally, it has been shown that i.c.v. administration of genetically engineered HSPCs directly into the brain results in widespread engraftment of cells with many characteristics of endogenous microglia.[18] However, the extent of engraftment and durability of HSPC-derived cells in the brain and how the route of administration effects the nature of engrafted cells are still poorly understood. To shine light on these crucial aspects, we performed a comprehensive animal biodistribution study following i.v., i.c.v., or a combination of i.v. and i.c.v. dosing of genetically engineered HSPCs into C57BL/6J mice after busulfan conditioning (Figure 1A).

Lineage negative (Lin-) HSPCs were isolated (Figures S1A and S1B) and transduced with a lentiviral vector encoding GFP (LVV.GFP) at greater than 74% transduction efficiency (range: 74.0%–84.5% GFP-positive cells; Figure 1B) and approximately 6 copies of integrated vector per genome (Figure S1C). No changes to the proliferative potential of the drug product were observed after transduction, as measured using the colony-forming unit assay Figure S1D). Recipient animals received 4 days of 25 mg/kg/q.d. (cumulative dose of 100 mg/kg) of busulfan prior to cell administration and lost 10%–15% of their bodyweight, which was rapidly regained post-treatment (Figure S1E). As expected, robust chimerism of all major cell types in the peripheral blood and bone marrow was observed in animals that received an i.v. dose of genetically engineered HSPCs but not in animals that only received genetically engineered HSPCs via i.c.v. (Figures 1C–1E and S2). Similarly, engraftment of cells throughout the peripheral organs was limited to animals that received an i.v. dose (Figure 1F). In the spleen, we detected integrated vector (Figure 1G), transgene expression (Figure 1H), and GFP protein expression (Figure 1I) at 16 weeks after infusion only in animals that received cells via an i.v. dose. As expected, we observed a significant correlation between the levels of GFP transcript and total amount of GFP protein in the periphery, though this correlation was not significant between vector copy number (VCN) and RNA (Figure 1J).

Within the brain, all three routes of administration led to widespread engraftment of GFP-positive cells throughout the rostral-caudal axis, including the cortex, hippocampus, choroid plexus, thalamus, and cerebellum. Qualitatively, we observed widespread engraftment at 16 weeks post-transplantation (Figures 2A and 2B) and then again at 12–13 months (Figure S3A). At 16 weeks, 78%–89% of GFP-positive (GFP+) cells from all routes of administration co-expressed the microglial marker Iba1 (i.v.: 78.40% ± 3.30%; i.c.v.: 88.67% ± 2.78%; i.v. + i.c.v.: 81.57% ± 3.20%; Figure 2C). No significant differences in GFP+ cell engraftment was observed across the rostral-caudal axis for all routes of administration (Figure S4). Strikingly and in contrast to previous reports,[18] we observed a significant difference in the level of engraftment between the i.v. and i.c.v. routes of administration. Animals dosed by i.v. alone had an average of 19.02% (range: 13.04%–25.44%) GFP+ engraftment in the microglia compartment compared with an average of 5.71% (range: 1.87%–8.26%) in animals dosed by i.c.v. alone (Figure 2D). Animals dosed using both routes of administration (i.v. + i.c.v.) had a similar level to animals dosed only via i.v. (range: 14.98%–26.64%; mean: 19.35% ± 1.57%; Figure 2D). Measurements of VCN per diploid genome (Figure 2E), transgene expression (Figure 2F) and GFP protein levels in the whole brain (Figure 2G) corroborated this difference in engraftment between i.v. and i.c.v. dosing. Statistically significant correlations were found between VCN, mRNA, and protein associated with the GFP transgene in the brain (Figure 2H). Of note, we observed a general lack of correlation of biodistribution metrics (VCN, mRNA, protein) in the peripheral tissues and the brain, consistent with previous clinical and preclinical reports[15,20,29] that engraftment in these two compartments are independent events that occur from separate cell populations derived from the drug product (Figure S5).

Single-cell transcriptional characteristics of HSPC-derived MLCs

The characteristics of HSPC-derived MLCs that engraft in the brain and their equivalence to microglia, which are derived from the embryonic yolk sac, remain contentious. Differences in the source of bone-marrow-derived progenitors (e.g., the bone marrow compartment versus an administered cell bolus), the nature of cell recruitment (e.g., native signaling versus induction from microglial ablation), and the state of the brain (e.g., healthy versus active disease) all influence MLCs and how they might compare to *bona fide* microglia. While transcriptional signatures that separate HSPC-derived MLCs and microglia have been described broadly, the single-cell heterogeneity of these populations and a high-resolution characterization of these differences in HSPC-GT have remained uncovered to date. Similarly, the comparison of transcriptional signatures of long-term engrafted MLCs that engraft via migration from the periphery (i.v. dosed) versus those that directly administered to the brain (i.c.v. dosed) has not been described.

To address these aspects, we combined cell sorting with high-throughput single-cell RNA sequencing (scRNA-seq) to characterize long-term engrafted MLCs and endogenous microglia from the same animals. We isolated endogenous microglia (GFP-negative [GFP-]) and MLCs (GFP+) from enzymatically dissociated whole brains by fluorescence-activated cell sorting (FACS) based on the expression of cell surface markers (Cd45+, Cd11b+, Cx3cr1+) from one male and one female mouse 12–13 months after either i.v. or i.c.v. administration of HSPCs (Figures S3B–S3G). After sequence processing and

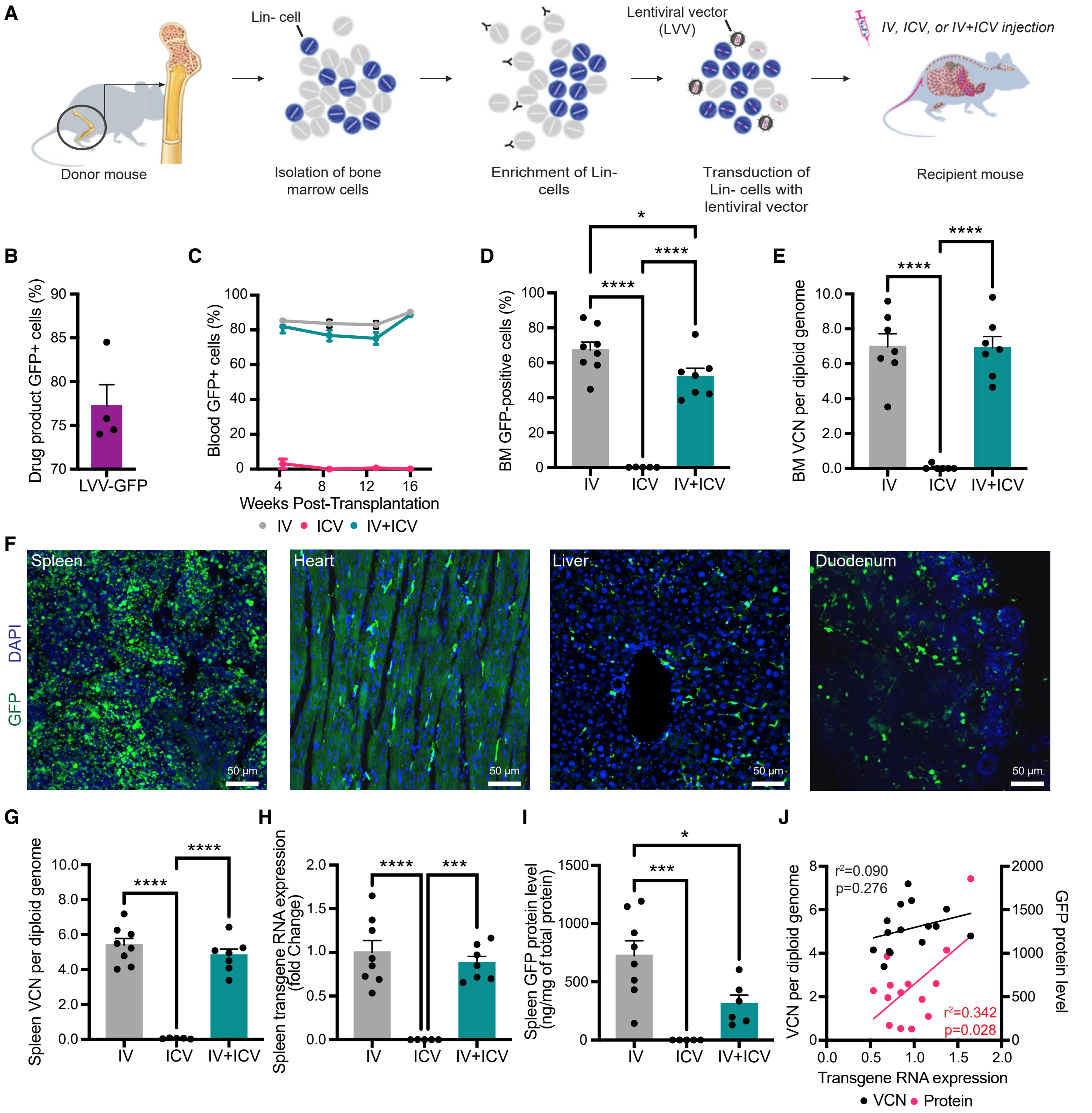

Figure 1. Peripheral engraftment HPSC-derived cells following administration via i.v., i.c.v., or i.v. + i.c.v.

(A) Total bone marrow cells were isolated from donor mice, enriched for lineage-negative (Lin-) hematopoietic stem/progenitor cells, and then transduced with a lentiviral vector (LVV.GFP). Busulfan-conditioned recipient animals received cell doses via i.v., i.c.v., or both (i.v. + i.c.v.). (B–D) Flow cytometry for GFP was performed on the transduced Lin-drug product, at 4 week intervals on peripheral blood, and on the bone marrow (BM) at necropsy (16 weeks post-transplantation). (E) Vector copy number (VCN) in the BM was determined by qPCR. (F) Peripheral organs were harvested, fixed, sectioned, and GFP-positive cells were observed in all organs examined for all i.v.-dosed animals examined, including the spleen, heart, liver, and duodenum. No such engraftment was observed in any animals dosed via i.c.v. alone. (G–I) VCN, transgene RNA expression, and GFP protein levels were measured in spleens from each treatment group at 16 weeks post-treatment. (J) Correlation analysis between splenic levels of RNA and VCN (black dots and bar) and splenic levels of RNA and protein (red dots and bar) for animals that received doses by i.v. or i.v. + i.c.v. Bars represent means ± standard error of the mean (SEM), * represent $p < 0.05$, *** represent $p < 0.001$, and **** represent $p < 0.0001$. Closed circles in each graph represent individual data points. For (C)–(E) and (G)–(I), a one-way ANOVA with Tukey's multiple post-hoc comparison test was conducted. For (J), the Pearson correlation coefficient for each correlation is shown.

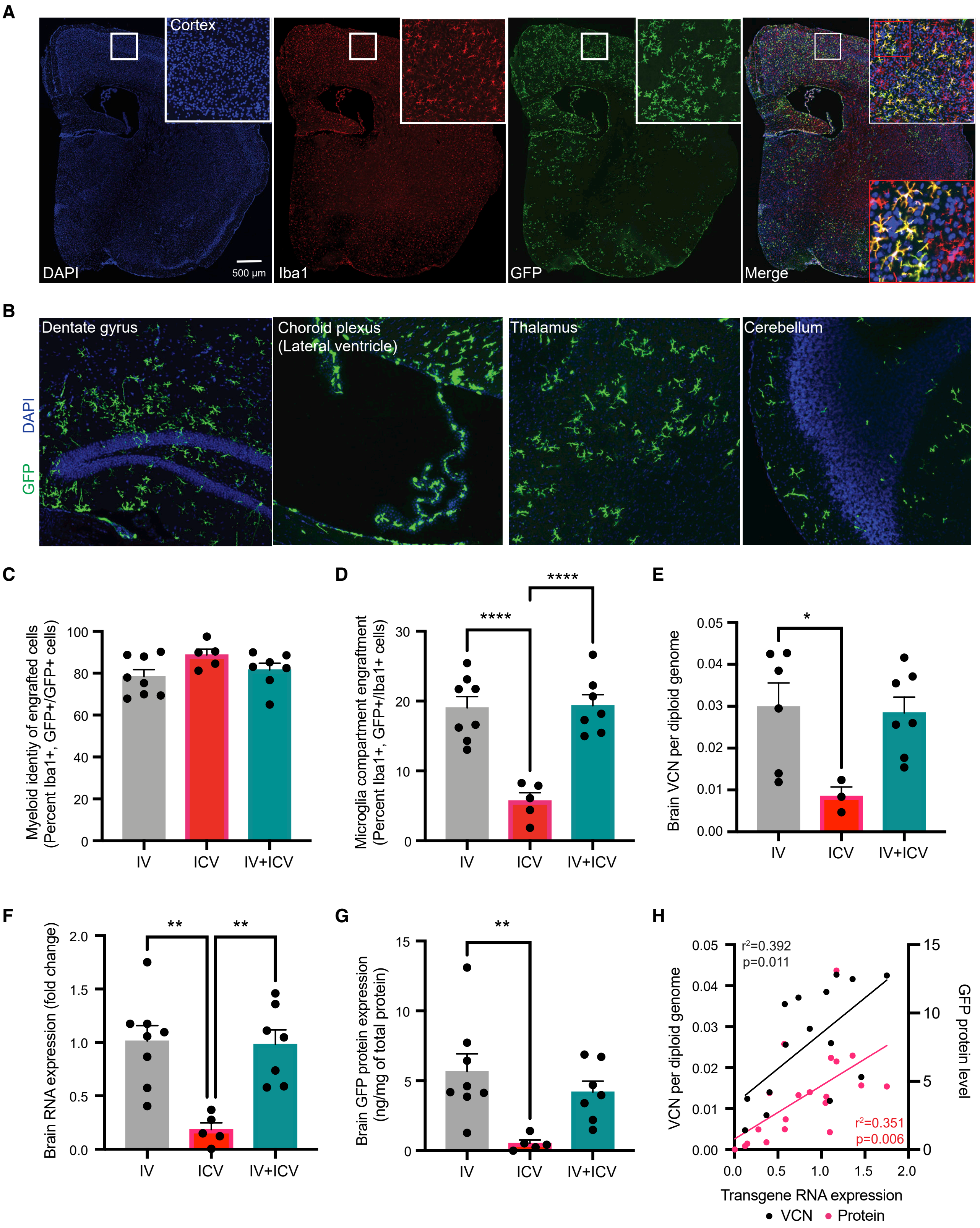

(legend on next page)

data analysis, we obtained a total of 29,085 individual scRNA profiles across the eight combined datasets for MLCs and endogenous microglia. We also generated comparator datasets from a 12-week-old, treatment-naive C57BL/6J animal for several cell populations: microglia (Cd45+, Cd11b+, Cx3cr1+), neurons (Cd45-, Thy1+, Acsa2+), astrocytes (Cd45-, Thy1-, Acsa2+), and peripheral blood mononuclear cells (PBMCs; Figure 3A). By t-stochastic neighbor embedding (tSNE) dimensionality reduction of the most variable genes, we observed that MLCs are in distinct but neighboring clusters to endogenous microglia (Figures 3B, 3C, and S6). The majority of PBMCs, neurons, and astrocytes occupied largely distinct single-cell clusters compared with microglia and MLCs except, notably, a population of PBMC circulating monocytes, which cluster with MLCs. Hierarchical clustering analysis confirmed that MLCs are similar to endogenous microglia and share higher similarity with bone-marrow-derived PBMCs than with neurons and astrocytes (Figures 3D and 3E). We then directly compared the normalized levels of individual transcripts in all GFP+ MLCs with that of their endogenous GFP- microglia counterparts to assess the degree of similarity between these two populations. We observed a strong correlation across most transcripts (Figure 3F; adjusted $R^2 = 0.9071$), supporting the notion that the transcriptome of HSPC-derived MLCs are distinguishable but largely similar to that of endogenous microglia.

We then analyzed in detail the single-cell heterogeneity of GFP+ MLCs and GFP- endogenous microglia in our treated mice. By tSNE dimensionality reduction of highly variable genes and Louvain clustering of single cells, we detected 8 main single-cell clusters when comparing these two populations. Notably, we observed a clear separation of MLC and microglia populations using clustering analysis (Figures 4A, 4B, and S7). GFP- endogenous microglia were enriched in clusters 1, 2, 4, and 6, while GFP+ MLCs were enriched in clusters 0, 3, and 5. Clusters 7 and 8 were also composed of a very small number of contaminating glia (astrocytes and oligodendrocytes), which we preserved as an outgroup comparison. The top 10 significantly enriched genes defining each cluster highlight the developmental differences between MLCs and microglia and potential differences in their inflammatory state (Figures 4C and 4D). Clusters containing mostly GFP- cells were enriched for markers associated with microglia homeostasis including *Mef2a*, *Hexb*, *Ctsc3*, *P2ry12*, *Selplg*, and *Sparc*, with this signature being most highly enriched in clusters 2 and 6. Clusters 1 and 4 showed relatively lower levels of homeostatic genes, a hallmark of mild microglia activation that is likely in part a result of the enzymatic isolation process. Notably, cluster 1 clearly separates from cluster 4 based almost entirely on the sex of the recipient animal, with differences driven largely by the expression of the X-inactivation genes *Xist* and *Tsix*. This sex difference was not observed in GFP+ MLC-enriched clusters, given that all donor animals for this experiment were males (Figure S8). Clusters containing mostly GFP+ MLCs were enriched with core transcriptional markers previously associated with bone-marrow-derived "CNS-associated macrophages" (CAMs) *Ms4a7*, *Mrc1*, *Pf4*, and *Stab1*.[30] Moreover, GFP+ enriched clusters also show stronger expression of activation markers of endogenous microglia including genes associated with reactive oxidation (*Cybb*, *Fyb*; cluster 0) and metabolism (*Apoe*, *Lyz2*; cluster 3) and genes that encode for the major histocompatibility complexes (MHCs) such as *Cd74*, *H2-Aa*, *H2-Eb1*, and *H2Ab1* (cluster 5).

To identify highly expressed markers that could distinguish MLCs from endogenous microglia, we first performed a differential gene expression analysis comparing the composite single-cell transcriptome of GFP+ and GFP- cells (Figure 4E). Homeostatic genes used as canonical microglia markers including *SallI*, *Csf1*, and *Tmem119* were enriched in GFP- endogenous microglia over GFP+ MLCs. Accordingly, Tmem119 expression was evident in endogenous microglia but largely excluded from GFP+ cells in the brain (Figure 4F). Genes enriched in MLCs include *Apobec1* and *Ms4a14*, markers that have been previously associated with bone marrow (BM)-derived, CAMs/peripheral nervous system (PNS)-associated macrophages when compared with endogenous microglia.[31,32] Gene Ontology (GO) and pathway analysis comparing the two populations highlight differences in genes associated with immune function, including chemokine production, cell adhesion, and responses to interferon, all processes associated with the known functional roles of endogenous microglia in the brain (Figure S9).

Our analysis comparing MLCs and microglia confirms and expands upon the information from previous studies on CAMs/PNS-associated macrophages, where expression of markers associated with inflammation in parenchymal microglia are enriched in BM-derived macrophages isolated from healthy animals without overt neuroinflammation.[29,32] In addition, our data show clear concordance with markers previously identified using bulk RNA-seq comparing MLCs and microglia after busulfan conditioning and i.v. dosing of HSPCs (Figure 5A).[29] We then compared our datasets to previous work characterizing the transcriptional profiles of CNS-invading, inflammatory macrophages that are mobilized from the periphery in the context of disease or injury.[33] Our comparison using this gene

Figure 2. Engraftment of MLCs in the brain following different routes of administration of genetically engineered HSPCs

(A) Representative image of a coronal slice from an i.v.-dosed animal visualizing Iba1, DAPI, and GFP at 16 weeks post-transplantation. Top right insert surrounded by white box is a digital magnification of a region of the primary motor cortex from the whole brain. Bottom right insert surrounded by red box is a magnification of the image in the white box. (B) Representative images from an i.v.-dosed animal at 16 weeks post-transplantation with GFP-positive cells located throughout the rostral-caudal axis of the brain, including the hippocampus, choroid plexus, thalamus, and cerebellum. (C) Quantification of GFP-positive engrafted cells that express the myeloid marker Iba1. (D) Quantification of Iba1+ cells that expressed GFP. (E–G) Quantification of integrated vector into the genome (VCN) via qPCR, transgene RNA expression via qRT-PCR, and GFP protein levels via ELISA in the brain for all treatment groups. (H) Correlation analysis of transgene RNA expression and VCN and transgene RNA expression and protein levels. Error bars represent means ± SEM, * represent $p < 0.05$, ** represent $p < 0.01$, and **** represent $p < 0.0001$. For (C)–(G), Tukey's multiple post-hoc comparison test was conducted. For (H), the Pearson correlation coefficient for each correlation is shown.

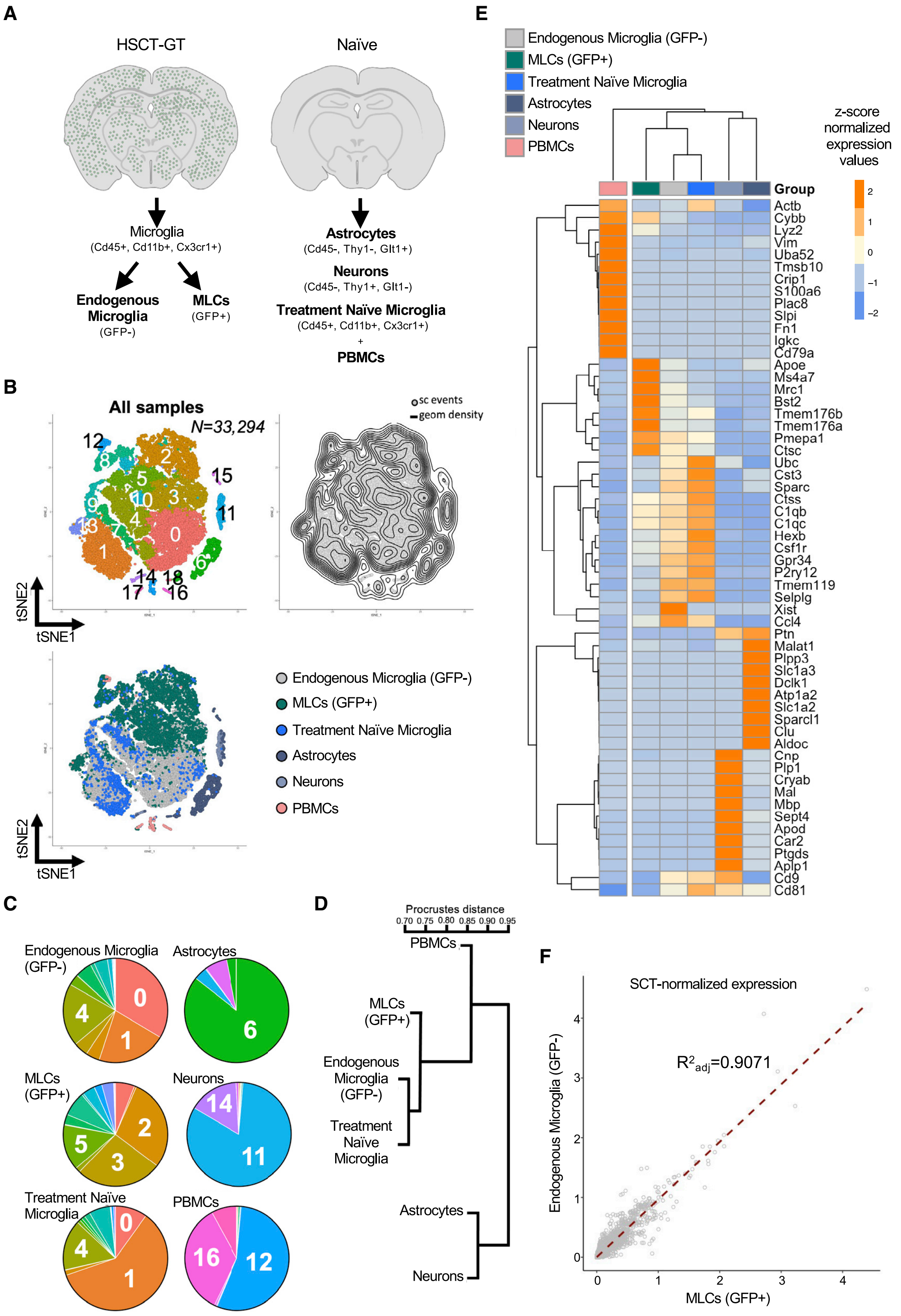

(legend on next page)

list associated with invading macrophages showed that our GFP+ MLCs show generally low expression of genes associated with invading macrophages and relatively higher expression of the homeostatic microglia signature (Figure 5B). These results suggest that certain differences we observed between MLCs and microglia may result from their distinct developmental origin rather than from distinct activation states.

Lastly, we wanted to investigate whether the route of administration impacted the transcriptional profile of MLCs. Surprisingly, and despite a vastly different engraftment history, we observed a significant overlap in the transcriptional signatures of i.v.-dosed GFP+ and i.c.v.-dosed GFP+ cells, with most transcripts showing strong correlation in expression (Figure S10A; R^2 = 0.9134). i.v. and i.c.v. GFP+ cells had similar representation in each cluster, apart from cluster 5 (MHC^{high}), which was enriched for i.v. GFP+ cells over i.c.v. GFP+ cells (Figure 4B). Expression of specific MHC genes was previously reported to be associated with border-associated macrophages (BAMs), which are largely yolk-sac-derived macrophages that occupy a niche near the blood-brain barrier.[34] This signal is consistent with the engraftment history of i.v.-derived MLCs, which must migrate across the blood-brain barrier. Accordingly, clustering analysis show differences between i.v. and i.c.v. groups in the expression of several MHC genes, including *H2-a*, *H2-b*, and *Cd74* (Figure S10B).[35]

LVV.GBA and LVV.GRN increase GCase/progranulin expression and secretion

Our characterization of MLCs and our assessment of biodistribution of gene-modified cells supports a wider application of HSPC-GT to address the peripheral and CNS components of neurodegenerative disease with well-defined genetic risk factors. Two such disorders are GBA-associated Parkinson's disease (GBA-PD) and progranulin-associated frontotemporal dementia (GRN-FTD), both of which are associated with the heterozygous loss of function of a lysosomal protein (beta glucocerebrosidase and progranulin, respectively). We generated lentiviral vectors expressing either codon-optimized human GBA (LVV.GBA) or codon-optimized human GRN (LVV.GRN) (Figure 6A) and transduced mouse macrophage RAW264.7 cells with increasing amounts of vector. Increased integrated vector copies per diploid genome predictably increased the amount of transgene-derived RNA transcript and protein expression for both vectors (Figures 6B–6Q). Importantly, we observed active beta-glucocerebrosidase (GCase) and human progranulin (hGRN) in the conditioned media even at low VCNs, supporting that lentiviral-mediated, supraphysiological expression of these proteins results in their secretion from macrophage-like cells (Figures 6E, 6G, 6M, and 6O). This suggests that GCase or progranulin could be secreted by MLCs for uptake by other cells within the brain.

HSPC-GT in a model of GBA-PD results in widespread distribution of gene-modified cells

Mutations in *GBA* are causative for the lysosomal storage disorder Gaucher disease and are among the most prevalent genetic risk factors for PD[36,37] and Lewy body dementia.[38] To assess the utility of lentiviral HSPC-GT for patients with PD associated with a *GBA* mutation (GBA-PD), we conducted a biodistribution study of key organs in homozygous Gba^{D409V} mice, a model of GBA-PD.[39] We isolated Lin- cells and transduced them with either a lentiviral vector encoding our previously characterized human GBA transgene (LVV.GBA) or a control GFP transgene (LVV.GFP) at a similar number of vector integrations per diploid genome (LVV.GBA: 3.12 ± 0.15, LVV.GFP: 2.93 ± 0.14; Figure 7A). As expected, the GCase activity in the cells transduced with the GBA transgene had significantly higher activity than those transduced with the GFP transgene (LVV.GBA: 3,418.10% ± 612.2%, LVV.GFP: 20.38% ± 4.31%; Figure 7B).

We then transplanted the genetically modified HSPCs into homozygous Gba^{D409V} knockin mice, which have lower levels of enzyme activity in the BM and brain compared with wild-type controls (Figures S11A–S11D). Transplantation of LVV.GBA HSPCs led to detectable and stable levels of VCNs in white blood cells (Figure 7C). Furthermore, we observed GCase activity in the plasma starting at 4 weeks post-transplantation (earliest time point) through 16 weeks (terminal time point). In contrast, there was minimal, if any, GCase activity in LVV.GFP-treated animals (Figure 7D). At 16 weeks, we observed engraftment in the spleen (Figure 7E), duodenum (Figure 7F), and BM (Figure 7G) in both LVV.GBA- and LVV.GFP-treated animals. Importantly, we measured higher levels of GCase activity in the BM of LVV.GBA-treated animals compared with animals transplanted with LVV.GFP HSPCs (LVV.GBA: 1,547.75% ± 102.2%, LVV.GFP: 11.06% ± 0.50%; Figure 7H). Within the brain, we saw detectable levels of integrated vector (Figure 7I) and transgene RNA expression (Figure 7J) in both animal groups. We observed higher levels of GCase protein (LVV.GBA: 134.4% ± 7.24%, LVV.GFP: 105% ± 9.16%; Figure 7K) and GCase enzymatic activity (LVV.GBA: 19.19% ± 1.93%, LVV.GFP: 13% ± 1.63%; Figure 7L) in the LVV.GBA cohort versus the LVV.GFP cohort. Taken together, we observed widespread and robust biodistribution of the

Figure 3. Single-cell comparison of MLCs, endogenous microglia, PBMCs, neurons, and astrocytes

(A) MLCs and endogenous microglia were isolated via enzymatic digestion, FACS purified, and then single-cell sequenced from i.v.- and i.c.v.-dosed male and female animals. PBMCs, neurons, astrocytes, and microglia were similarly isolated from a treatment-naive animal. (B) Global tSNE plot generated from a total of 33,294 cells (n = 5 mice; 12 samples). The plot on the left shows the Louvain clustering (resolution 0.4), while the plot on the right shows the density of single-cell events on the map. (C) Pie charts showing the cluster breakdown by sample, with labels showing the top represented clusters for each sample type. Seven MLC-enriched clusters (2–5, 8, 10, 12, 13, 15), 5 microglia-enriched clusters (0, 1, 4, 7, 9), 1 neuron cluster (11), 1 astrocyte cluster (6), and 4 PBMC clusters (14, 16–18) were identified. (D) Unsupervised clustering based on the global expression profile of each sample type. (E) Heatmap showing top 10 differentially expressed genes for each sample type. Dendrogram at the top shows samples clustering based on expression profiles of these genes. (F) Correlation analysis of endogenous microglia versus MLCs based on normalized single-cell gene-expression data.

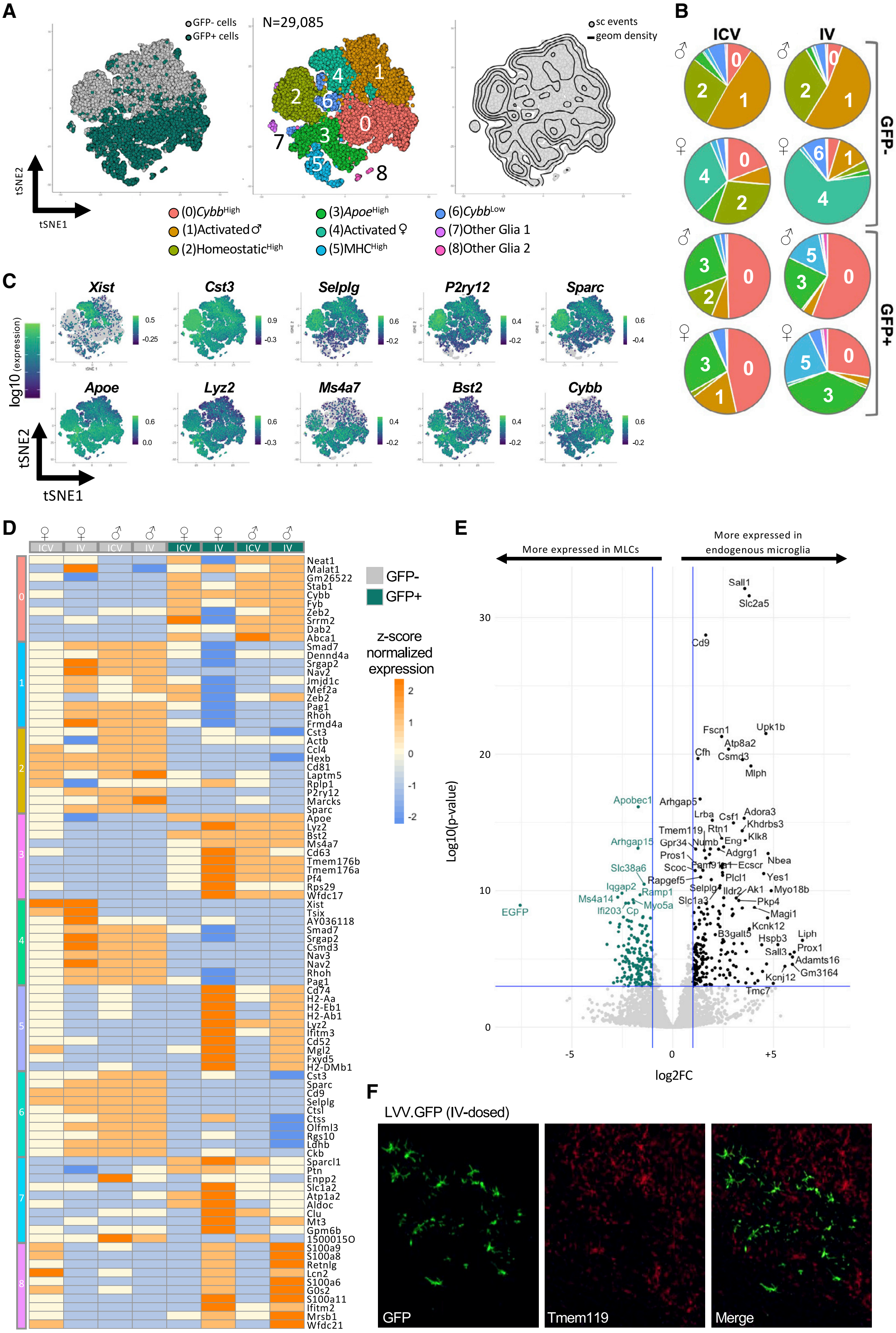

(legend on next page)

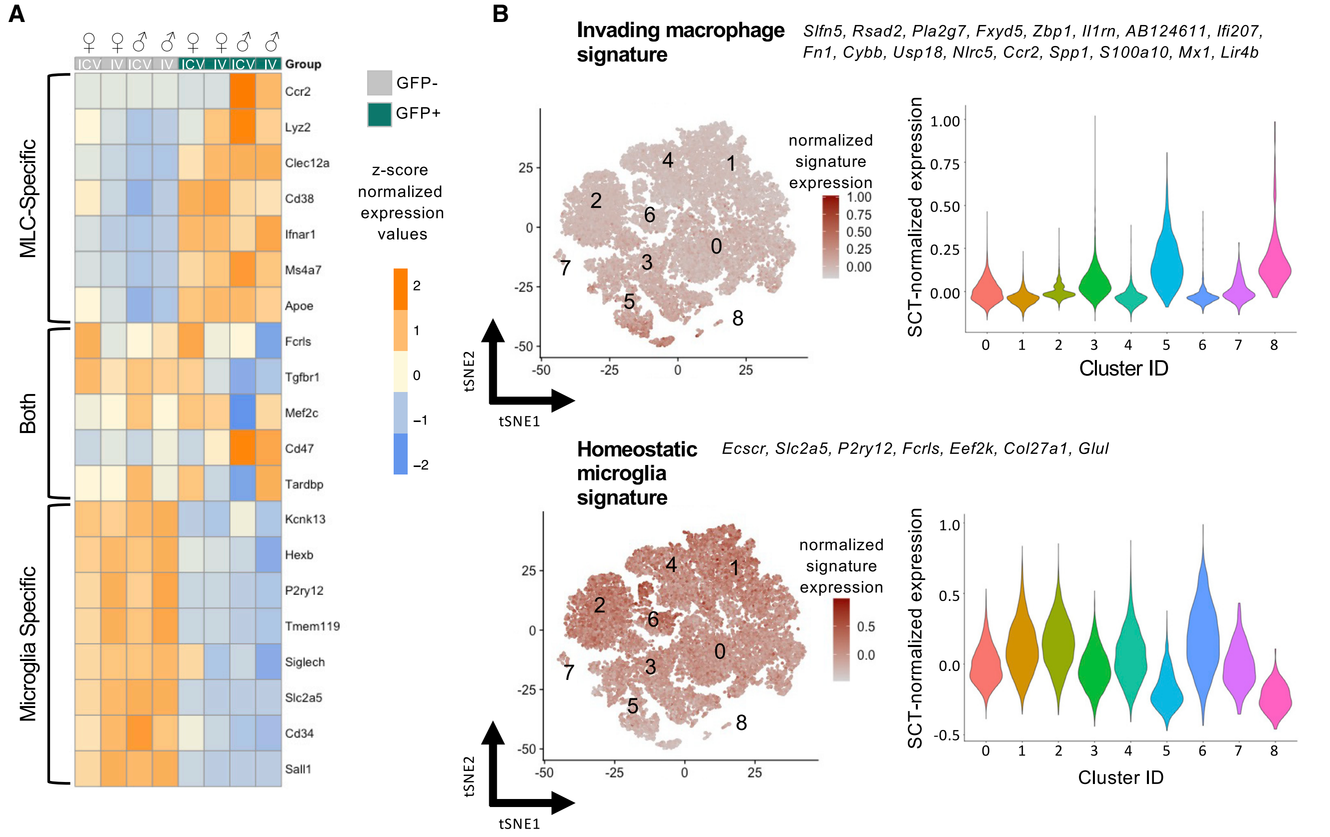

Figure 5. Projection of MLC-, macrophage-, and microglia-specific markers on the single-cell landscape

(A) Heatmap showing the single-cell normalized average expression values of MLC-specific, microglia-specific, and shared gene markers as described in Shemer et al.[29] (2018) in GFP- and GFP+ cells from treated mice. (B) Projection of invading versus homeostatic microglia gene signatures as described in DePaula-Silva et al. (2019) over the tSNE map of Figure 4A (left panels), and violin plots showing the expression of these signatures in the clusters identified in Figure 4A (right panels). (C) Violin plots showing the expression of genes associated with invading macrophages/monocytes (left panels) or homeostatic microglia (right panels) as described in Haage et al.[33] (2019) in GFP- (gray squares) or GFP+ (green squares) treated mice labeled according to their sex and identification number.

gene-modified cells in key organs, including the gastrointestinal tract, spleen, BM, and CNS.

HSPC-GT in a model of GRN-FTD increases progranulin and may address lysosomal dysfunction

We then applied our HSPC-GT platform to treat progranulin deficiency in a mouse model of genetic FTD (GRN-FTD). GRN-FTD is a familial form of neurodegeneration caused by the haploinsufficiency of GRN/progranulin, a lysosomal precursor protein that is widely expressed in the brain and secreted from microglia during overt disease.[40] Heterozygous and homozygous Grn^{R493X} mutant mice, a model of GRN-FTD,[41] were dosed with Lin- cells transduced with high or low vector doses of LVV.GFP or LVV.GRN after standard (100 mg/kg) or increased (125 mg/kg) levels of busulfan conditioning. As in our previous studies, we observed biodistribution via vector integration quantification and transgene expression in the BM and the brain for all treatment groups for LVV.GFP and LVV.GRN (Figures 8A, 8B, 8E, 8F, and S12A–S12D). Across all

Figure 4. Characterization of the single-cell transcriptional landscape of MLCs versus endogenous microglia in treated mice

(A) Global tSNE plot generated from a total of 29,085 single cells from endogenous microglia (GFP) and MLCs (GFP+, in green) (n = 4 mice; 8 samples). The plot on the left shows single cells separated in the main GFP- (gray dots) and GFP+ (green dots) groups. The plot in the middle shows the Louvain clustering (resolution 0.2). Putative functional associations and/or key markers differentiating each cluster are listed below. The plot on the right shows the density of single-cell events in the map. (B) Feature plots showing expression of individual genes characterizing the top (microglia enriched) or the bottom (MLC enriched) sections of the map. (C) Heatmap showing single-cell normalized expression of the top 10 differentially expressed for each cluster compared with the rest of the map (note: clusters might share top represented genes). Columns represent GFP- (gray squares) and GFP+ (green squares) cells from treated mice labeled according to their sex, identification number, and route of administration (i.c.v., i.v.). (D) Volcano plot highlighting differentially expressed genes between GFP+ MLCs (left-hand side highlighted in green) and GFP+ microglia (x axis log2 fold change in average gene expression, y axis log). (E) Representative IHC images of GFP and Tmem119 from an i.v.-dosed animal.

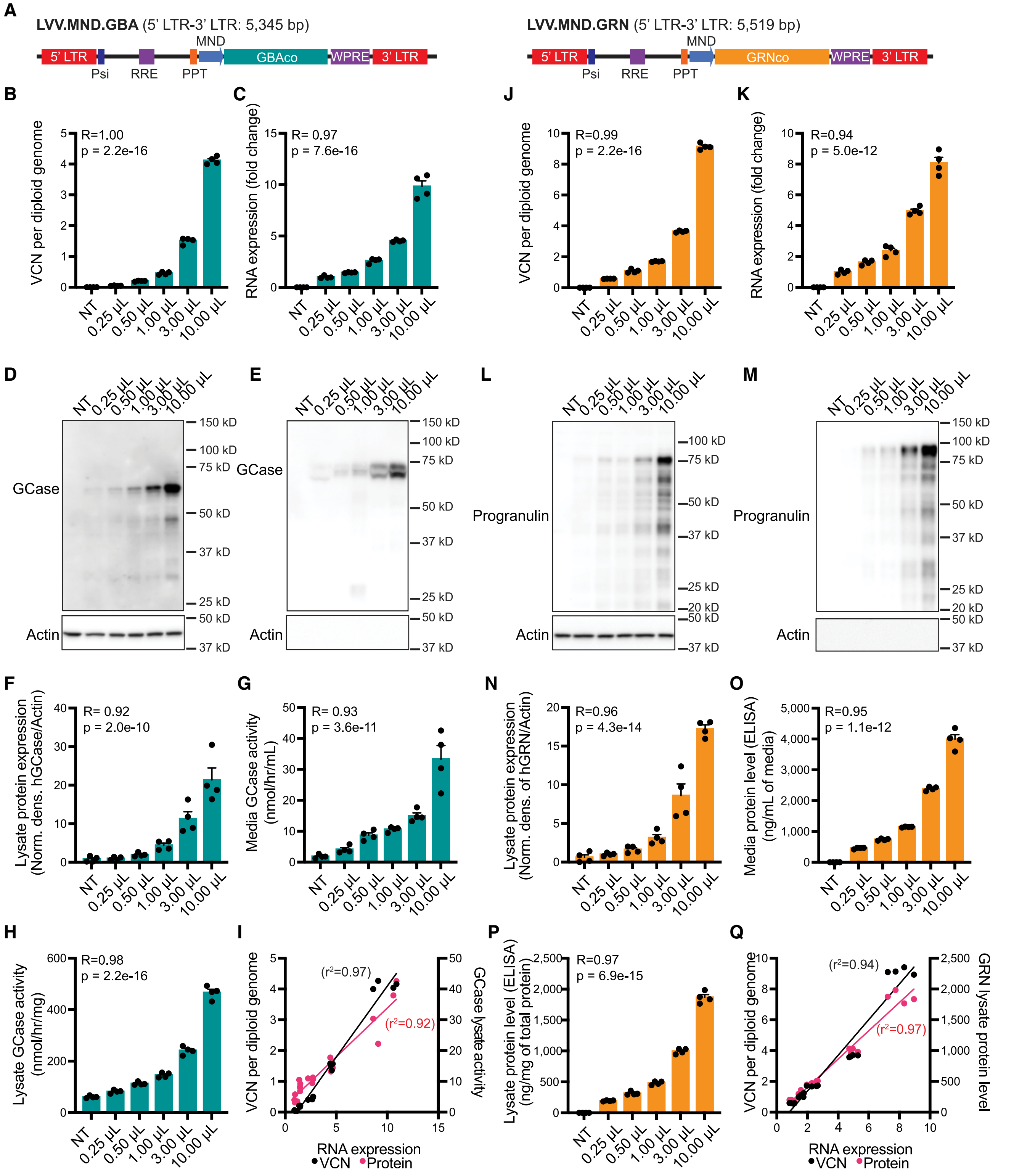

(legend on next page)

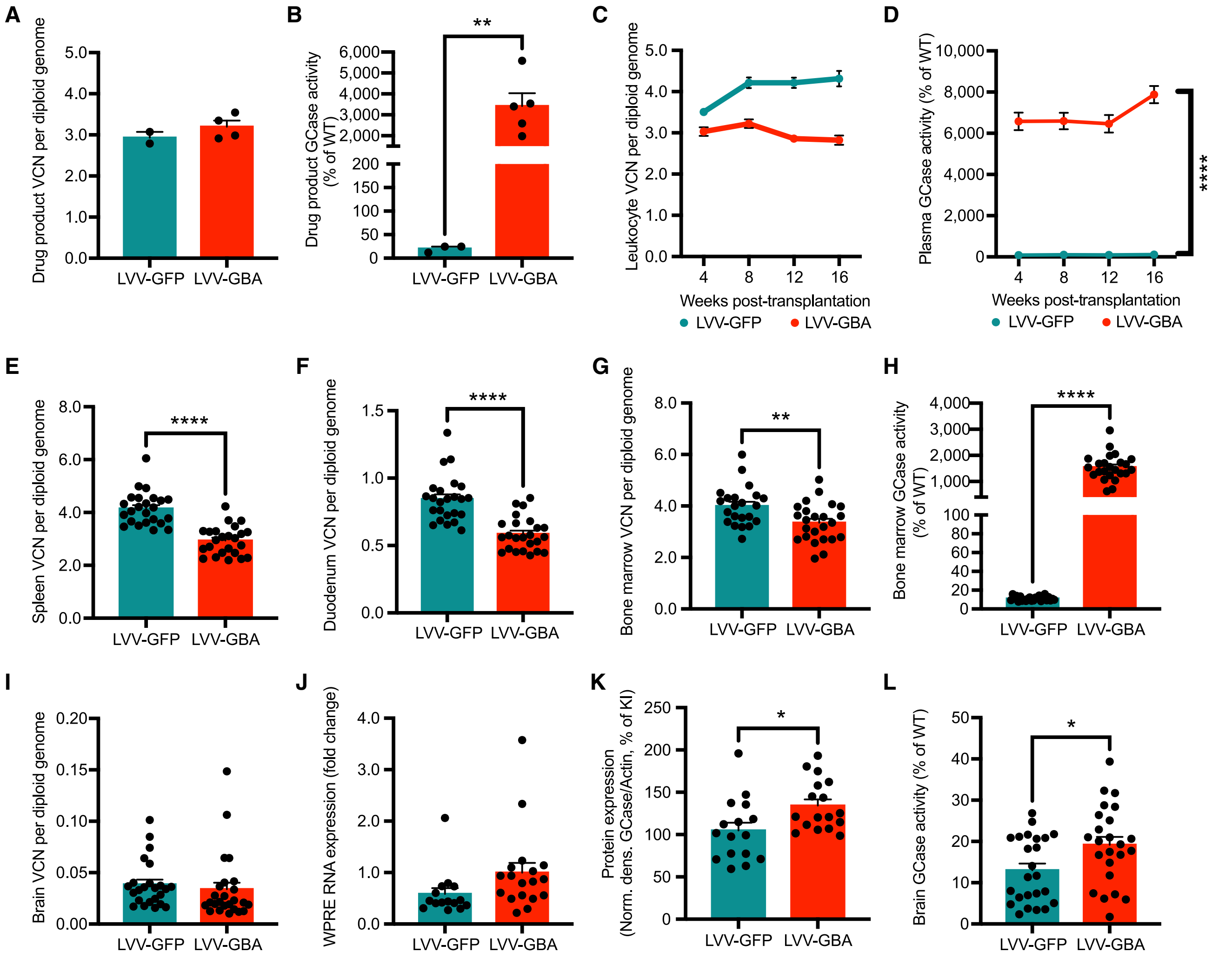

Figure 7. Biodistribution analysis after transplantation of LVV.GBA modified HSPCs into *Gba* mutant mice

(A) VCN in the drug product in the LVV-GFP- and LVV-GBA- treated groups kept in culture for 4 days *in vitro* (DIV). (B) GCase enzymatic activity in the drug product at DIV 4. (C and D) VCN and GCase enzymatic activity in white blood cells and plasma, respectively, measured longitudinally every 4 weeks post-transplantation. (E and F) VCN in spleen and duodenum at 16 weeks post-transplantation. (G and H) VCN and GCase enzymatic activity in the BM at 16 weeks post-transplantation. (I–L) VCN, transgene RNA expression, GCase protein levels, and GCase enzymatic activity in the brain at 16 weeks post-transplantation. Bars represent means ± SEM, * represent $p < 0.05$, ** represent $p < 0.01$, and **** represent $p < 0.0001$. For (A), (B), and (E–L), t test was used for statistical analysis.

LVV.GRN-treated groups, we measured supraphysiological levels of human progranulin via ELISA in the plasma and the BM, equivalent to an average of 5-to 11-fold above previously reported wild-type levels (Figures 8C and 8D).[42] We also observed human progranulin levels in whole-brain lysate of LVV.GRN-treated animals, equivalent to 30%–50% of previously reported mouse progranulin levels in the

Figure 6. Characterization of lentiviral vectors for GBA and GRN in a mouse macrophage cell line

(A) Schematic of integrating component of lentiviral vectors from the 5′ long terminal repeat (LTR) to 3′ LTR. RRE, Rev response element; PPT, polypurine tract; MND promoter, myeloproliferative sarcoma virus enhancer, negative control region deleted, dl587rev primer-binding site substituted; WPRE, woodchuck hepatitis virus post-transcriptional regulatory element. (B–I) LVV-GBA increased integrated VCN (B), transgene RNA expression (C), GCase protein expression in lysates (D and F) and media (E), GCase enzymatic activity levels in the lysate (H) and conditioned media (G). Correlation analysis of VCN per diploid genome to transgene RNA expression and transgene RNA expression to lysate GCase activity (I). (J–Q) LVV-GRN increased integrated VCN (J), transgene RNA expression (K), and progranulin protein levels in the lysate (L, N, and P) and conditioned media (M and O). Correlation analysis fo VCN per diploid genome to transgene RNA expression and RNA transgene expression to lysate GRN protein levels (Q). Bars represent means ± SEM. Pearson correlation analysis is shown where indicated, correlating the VCN to RNA expression for cells transduced with LVV.GBA (I) and LVV.GRN (Q).

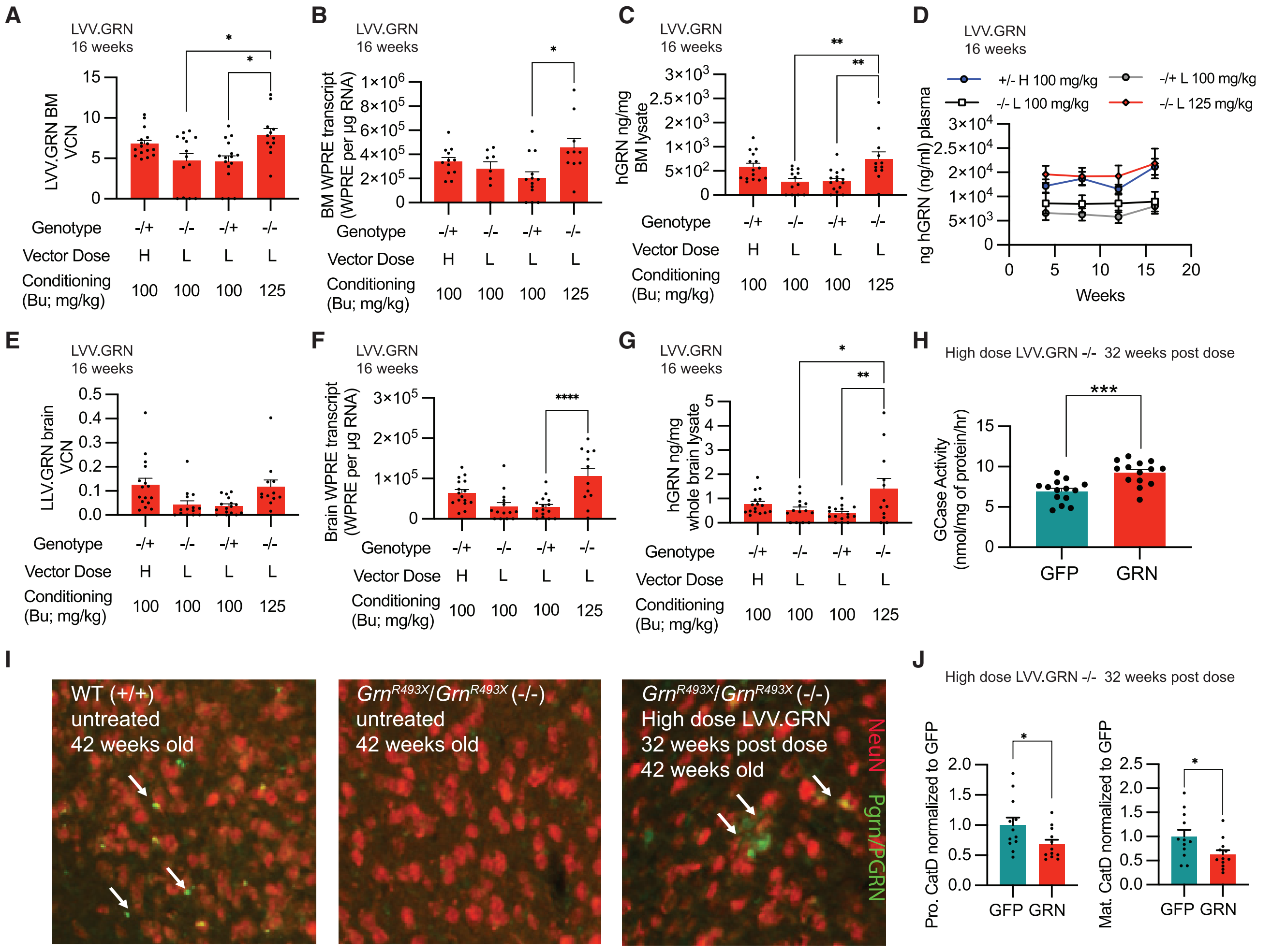

Figure 8. Genetically engineered HSPC administration in a mouse model of GRN-FTD shows widespread GRN in the periphery and the brain

(A–C) VCN, transgene RNA, and human progranulin protein measurements in BM for all LVV.GRN-treated groups at 16 weeks post-transplantation. (D) Longitudinal human progranulin protein measurements in plasma for all LVV.GRN-treated groups up to 16 weeks post-transplantation. (E–G) VCN, transgene RNA, and human progranulin protein measurements in brain for all LVV.GRN-treated groups at 16 weeks post-transplantation. (H) GCase activity in whole brain lysate of homozygous mutant *Grn* mutant mice treated with LVV.GFP and LVV.GRN at 32 weeks post-transplantation. (I) IHC imaging of neurons (NeuN, red) and progranulin (PGRN/Pgrn, green) in the cortex of untreated wild-type, homozygous mutant *Grn* mutant mice, or homozygous mutant *Grn* mutant mice treated with LVV.GRN. (J) Quantification of levels of the pro form and the mature form of cathepsin D (CatD) in whole-brain lysates of homozygous mutant *Grn* mutant mice treated with LVV.GFP and LVV.GRN at 32 weeks post-transplantation. Bars represent means ± SEM. Tukey's multiple post-hoc comparison test was conducted for all statistical measures shown, with only significant differences ($p < 0.05$) indicated (* represent $p < 0.05$, ** represent $p < 0.01$, **** represent $p < 0.0001$).

brain (Figure 8G).[43] Using immunohistochemical visualization of mouse and human progranulin, we observed progranulin+ cells in the cortex of LVV.GRN-treated homozygous mutant animals, consistent with the engraftment of LVV.GRN+ cells in the brain (Figure 8I). We observed no progranulin staining in treatment-naive *Grn* mutant mice and saw restoration of progranulin puncta after LVV.GRN HSPC-GT. Many, but not all, of these cells colocalized with the microglia marker Iba1, consistent with our previous experiments (Figure S13). Finally, and notably, increased levels of busulfan conditioning resulted in significantly increased VCNs, transgene expression, and GRN protein levels in the periphery and the brain (Figures 8A–8G).

While the reasons GRN deficiency leads to FTD are still unclear, progranulin is thought to play a role in regulating lysosomal homeostasis.[43] The protein and activity levels of several lysosomal enzymes, including decreased GCase and increased cathepsin D (CatD) have been observed in mouse models of GRN-FTD and human brain tissue from GRN-FTD patients.[44,45] Other models of GRN-targeted therapeutics have shown the ability to normalize these changes after treatment.[44] We observed similar changes of lysosomal dysfunction after treatment, with LVV.GRN-treated homozygous *Grn* mutant animals 32 weeks post-transplantation showing an average of 33% increased GCase activity levels compared with LVV.GFP-treated groups (Figure 8H) and decreased

mature CatD protein (Figure 8J) compared with LVV.GFP-treated groups.

DISCUSSION

We show here that HSPC-GT using an i.v. route of administration in mice results in the widespread engraftment of genetically engineered cells throughout the periphery and engraftment of MLCs in the brain. Similar levels of engraftment in the brain were previously reported by others.[28,46] In contrast, when HSPCs are administered via i.c.v., we observed that engraftment is limited to the CNS. Strikingly, and in contrast to earlier work,[18] we observed significantly more engrafted MLCs using i.v. administration compared with i.c.v. administration. While our methods and time points were similar for all key aspects, subtle technical disparities could explain the difference (e.g., cell dose, injection location, and/or cell-administration timing). Further studies are needed to explore these parameters and how they affect engraftment after i.c.v. administration of HSPCs, if at all. We also observed that HSPC-derived MLCs were durably engrafted within the brain over a year post-transplantation and that these cells bore many of the transcriptional hallmarks of microglia, which suggests that they have the capacity to self-renew much like endogenous microglia, though careful lineage-tracing experiments would be required to explicitly confirm this. This is consistent with long-term follow up of HSPC-GT in non-human primates, where HSPC-derived myeloid cells in the brain were observed 10 years post-transplantation after i.v. dosing.[15] It is similarly consistent with the durable phenotypic rescue in most patients enrolled in clinical trials for MLD and CALD who receive their cell doses i.v.[47] Importantly, these data strongly support the use of murine models for preclinical and translational research, as they can effectively recapitulate the long-term engraftment dynamics of MLCs observed in humans.

Our comparison of MLCs with endogenous microglia suggests that most transcriptional features of myeloid cells in the brain are niche dependent, as the two cell populations are largely similar. This observation parallels the ones from studies where monocytes and macrophages isolated from various tissues take on functional and transcriptional characteristics of tissue-resident macrophages of the lung, kidney, and liver when transplanted.[48–51] Furthermore, we show here that MLCs do not exhibit the signatures of invading macrophages associated with disease and injury, likely because these signatures are niche dependent and reflect the damaged status of the brain. Our data show, however, that MLCs and microglia can be clearly distinguishable by specific genes not reprogrammed during engraftment, such as canonical genes associated with resident microglia like *Tmem119*. It is likely that this is in part driven by the developmental differences between BM-derived myeloid cells and embryonic yolk-sac-derived microglia. How HSPC-derived MLCs and microglia differ on a functional level as a result of these differences in developmental origin will require further study, though as stated, our characterization shows that the transcriptional signatures are highly similar. The differences we observe in MLCs versus endogenous microglia will be informative for the further development of MLCs as a therapeutic modality and to understand what may underlie any functional differences between them and endogenous microglia.

Our work applying HSPC-GT in murine models of neurodegenerative disease shows that for both GBA-PD and GRN-FTD, engrafted cells have the capacity to produce potentially therapeutic proteins at significant levels. Notably, gastrointestinal issues are a common symptom in patients with PD, and mutations in *GRN* are implicated in cardiac dysfunction.[52,53] A systemic GT approach like HSPC-GT conceivably has advantages over other platforms including AAV GTs, where therapeutic effect is generally limited to either the periphery or the CNS. However, if CNS-restricted expression is therapeutically advantageous, our data suggest that i.c.v. administration of genetically engineered HSPCs can achieve this.

Furthermore, previous work has demonstrated that AAV administration into the brain parenchyma leads to extremely high levels of transgene expression close to the injection site but a vastly lower expression level in distal brain regions.[54,55] Importantly, the cell type that expresses the therapeutic protein will depend on the AAV capsid and promoter used.[56] Conversely, we showed here that the tiling behavior of MLCs expands the reach of HSPC-derived cells to the whole brain, resulting in an even and widespread distribution of genetically modified cells. It is worth noting that AAV-mediated delivery of GBA and GRN to neurons in mouse models of GBA-PD and GRN-FTD have been successful in modifying disease-associated phenotypes.[44,57] Our biodistribution studies and our initial biochemical characterization of lysosomal dysfunction in *Grn* mutant mice support the capacity of reaching similar results with HSPC-GT. Further characterization is required to determine (1) whether there are potential changes to the characteristics of MLCs carrying a therapeutic transgene and (2) if HSPC-derived MLCs can provide the levels and modality of transgene expression necessary to correct the wider functional changes in the brain associated with these disorders, with careful inclusion of comparison to wild-type animals. These follow-up study will elucidate the true therapeutic potential of this platform.

While this work addresses fundamental questions around HSPC-GT in the CNS, such as the identity of MLCs relative to endogenous microglia and the durability of MLC engraftment, there are broader questions that remain unresolved. These include understanding how the myeloablative agent acts in the CNS. Our results clearly demonstrate that busulfan conditioning is instrumental in enabling brain engraftment, with higher doses leading to higher engraftment and protein expression. Notably, busulfan conditioning may also be associated with cellular senescence of endogenous microglia, making increased busulfan conditioning a potential detriment despite increased engraftment.[58] Recent preclinical work has highlighted the use of CSF1R inhibition after treatment with busulfan, reporting up to 90% engraftment of the microglial niche, suggesting the potential for using other conditioning regimens outside of busulfan.[59] In addition, the identity of the cell that crosses the blood-brain barrier (e.g., HSPC, myeloid progenitor cell) after i.v. administration or that first engrafts after i.c.v. administration is unclear. It is also worth

examining if injection of myeloid cells alone could achieve similar levels of engraftment. Future studies should assess what the window of opportunity is for cells to cross the blood-brain barrier following myeloablation and how genetic payloads might change engraftment of MLCs.

In conclusion, our work provides critical information substantially expanding our understanding of the nature of MLCs derived from long-term HSPCs and opens new avenues for the potential use of genetically engineered MLCs for the treatment of common neurodegenerative disorders such as FTD and PD.

MATERIALS AND METHODS

Mouse models and tissue collection

All experiments were carried out with proper oversight by institutional review boards for animal care. Comparisons of i.v. and i.c.v. routes of administration were completed using wild-type (WT) C57BL/6J mice (Jackson Laboratory, stock number 000664). Proof-of-concept experiments for GBA-PD were completed using homozygous $Gba^{D409V/D409V}$ mutant mice (Jackson Laboratory, C57BL/6N-$Gba^{tm1.1Mjff}$/J, stock number 019106)[39] and WT C57BL/6NJ mice (Jackson Laboratory, stock number 005304) as controls. Proof-of-concept experiments for GRN-FTD were completed using heterozygous and homozygous Grn^{R493X} mutant mice on the C57BL/6J background.[41] Donor and recipient animals for all experiments were 8–12 weeks of age. Terminal collection for all animals occurred 14–16 weeks after cell administration except for the long-term cohort for scRNA-seq experiments (12–13 months post-transplantation) and the long-term cohort for the GRN-FTD proof-of-concept study (32–36 weeks post-transplantation). At necropsy, animals first underwent whole-body transcardial perfusion with either heparinized saline or PBS followed by tissue harvesting. Samples for biochemistry analysis were flash frozen and stored at -80°C. Samples for immunohistochemistry (IHC) analysis were fixed in 4% PFA in PBS overnight at 4°C.

Generation of genetically engineered HSPC drug product

For all studies, Lin- cells were enriched from total BM isolated from femurs and tibias of donor mice (6–12 weeks of age) using the EasySep Mouse Hematopoietic Progenitor Cell Isolation Kit (STEMCELL Technologies) in conjunction with the RoboSep-S (STEMCELL Technologies) automated cell-isolation machine and confirmed by flow analysis. Cells were stained for lineage markers with a PE-Cy5-conjugated lineage marker cocktail including B220, Ter119, Tcrb, Cd8a, Cd3ε, Cd4, Ly6g/Ly6c, and Cd11b and with hematopoietic stem cell markers APC-conjugated anti-Cd117 (c-Kit) and PE-conjugated Ly6a/e (Sca-1) (BD Biosciences). Lin- cells were transduced with a lentiviral vector in a cell incubator at 2×10^6 cells/mL in either StemSpan SFEM II (STEMCELL Technologies) or StemMACS HSC Expansion Media (Miltenyi Biotec) growth media freshly supplemented with TPO (10 ng/mL), SCF (100 ng/mL), and FLT3 (50 ng/mL). Approximately 16–22 h later, cells were collected, washed at least three times with DPBS (without Ca^{2+} and Mg^{2+}), and resuspended in an appropriate volume for dosing. A small number of cells (3,000–4,000 cells) were set aside for use in the colony-forming unit assay using M3434 media (STEMCELL Technologies) and were analyzed 7–10 days later.

Conditioning of recipient animals and drug-product administration

Four days prior to cell administration, recipient animals (6–12 weeks of age) received daily i.p. injections of busulfan (Busulfex, Otsuka Pharmaceutical) at 25 mg/kg for a cumulative dose of 100 or 125 mg/kg. For comparisons of routes of administration, drug-product pools were generated from an equal number of male and female mice. For scRNA-seq experiments, drug-product pools were generated from male mice only. For both i.v. and i.c.v. administration, animals were dosed with 5×10^5 cells. i.c.v. administration was performed using a stereotaxically guided Hamilton syringe and infusion system (Harvard Apparatus). The single infusion was performed unilaterally in the lateral ventricle in the following coordinates: AP = -0.5 mm (anterior from bregma); ML = +1.0 mm; DV = -1.75 mm. For animals dosed using both i.v. and i.c.v., animals were dosed with 5×10^5 for both routes for a total of 1×10^6 cells. Five days after drug-product administration, all animals despite route of administration received an additional i.v. administration of non-transduced total BM cells (5×10^5 cells in 50 μL per animal, resuspended in DPBS without Ca^{2+} or Mg^{2+}) to support the survival of animals that only received i.c.v. administration of drug product. Subsequent studies (GBA-PD and GRN-FTD proof-of-concept studies) only administered drug product i.v., and the second total BM administration was eliminated. Additionally, in these studies, donors and recipients were sex matched, and for the GRN-FTD proof-of-concept study, an additional analysis of 5 days of busulfan conditioning was added.

Lentiviral vector generation

VSV-G pseudotyped third-generation self-inactivating lentiviral vectors for LVV.GFP, LVV.GRN, and LVV.GBA were commercially generated (University of Cincinnati Vector Production Facility, VIVEbiotech, VectorBuilder) using HEK293T cells and standard protocols. Lentiviral vector functional titers ranged from 1.7×10^8 to 3×10^9 TU/mL.

Sample preparation and molecular characterization

Genomic DNA (gDNA) was isolated using the QIAamp DNA Mini QIAcube Kit (Qiagen) in conjunction with the QIAcube (Qiagen). Immortalized cell line gDNA was isolated using the DNeasy Blood & Tissue kit (Qiagen). VCN quantification was completed either by digital droplet PCR (ddPCR) or quantitative PCR (qPCR). Specific primers targeting the WPRE element (WPRE v1) or HIV Psi element were used to detect the integrated lentiviral vector, and specific primers targeting *Gtdc1* or *Tfrc* were used as genomic reference (Table S1).

RNA isolation and reverse transcription for murine samples from the GRN-FTD proof-of-concept study was completed using the QIAsymphony RNA kit (Qiagen). Absolute quantification of

transgene copies per microgram of tissue was completed using a standard curve of *in-vitro*-transcribed WPRE RNA and specific primers and probes targeting WPRE (WPRE v2; Table S1). RNA isolation and reverse transcription for all other samples were completed using the PureLink RNA Mini kit (ThermoFisher) followed by SuperScript IV VILO with ezDNase (ThermoFisher). Specific primers and probes targeting the WPRE element (WPRE v1; Table S1) of the integrating lentiviral vector and *Actb* (ThermoFisher), as a housekeeper, were used. Primers and probes, cDNA, and TaqMan Fast Advanced Master Mix (ThermoFisher) were combined according to the manufacturer's guidelines and amplified using a QuantStudio 7 Flex (ThermoFisher). The $2^{-\Delta\Delta Ct}$ method was used to calculate transgene expression.

Protein was extracted using a detergent-based lysis buffer dependent on the assay followed by removal of cellular debris by centrifugation at 14,000 × *g* for 15 min. Protein concentration was determined using the Pierce BCA Protein Assay Kit (ThermoFisher). SDS-PAGE was used to measure beta GCase and progranulin protein expression. GCase enzymatic activity was measured using the artificial substrate 4-methylumbelliferyl b-D-glucopyranoside (4-MUG) following a similar method to that previously published.[60,61] Progranulin protein levels were measured using a human-specific ELISA (Quantikine and DuoSet; R&D Systems) according to the manufacturer's instructions, with minor modifications for the measurement of human progranulin in brain lysates. For brain samples, reagents were diluted in 0.05% Tween 20 in Tris-buffered saline (TBS). Seventy-five μg of protein was loaded of mouse brain lysate and 1.5 μg of protein was loaded for mouse BM, and plasma was measured at a final dilution of 1:500 for the human progranulin ELISA assay. Thirty to 500 ng of protein was used to measure transgene expression of progranulin in RAW264.7 cells. Animal cohort sizes and sample numbers for all analysis shown are listed in Table S2.

Data and materials availability

Transcriptomic data discussed in this publication have been deposited in NCBI's Gene Expression Omnibus (GEO) database and are available through GEO: GSE201756. Additional data may be made available from AVROBIO, Inc., upon request with the appropriate material transfer agreement. All other data are available in the main or the supplemental information.

Additional methods and materials are included within the supplemental information.

SUPPLEMENTAL INFORMATION

Supplemental information can be found online at https://doi.org/10.1016/j.ymthe.2022.05.022.

ACKNOWLEDGMENTS

We thank all the members of AVROBIO, Inc., for their continued support of our work. We would like to thank Maurine Braun, Maria Grigorova, Christine Oborski, Daniella Pizzurro, Richard Pfeifer, Jurgen Poci, Chris Treleaven, Steven Tyler, Claudia Harper, Carolina Romano, Steven Avruch, Monique da Silva, Geoff MacKay, and Deanna Petersen for scientific support and assistance. We would like to also thank Taneli Heikkinen, Teija Parkkari, Sarah Davis, Karen Wong, Daphne Gordon, and Jaime Nederhoed at Charles River Laboratories (CRL) for study execution and diligent care of study animals. Additionally, we thank Scott W. Allen (BioAgilytix), Kelly Colletti (CRL), Katherine Domingue (QPS), Romain Genard (CRL), Lauriane Padet (CRL), Kaisa Paldanius (CRL), Taina-Kaisa Stenius (CRL), and Jenifer Vija (CRL) for their contributions to sample analysis. The *Grn*^{R493X} mice were generated in the Robert Farese Laboratory at The J. David Gladstone Institutes with grant funding provided by the Consortium for Frontotemporal Dementia Research and under a subaward of a University of California, San Francisco grant from the Alzheimer's Disease Research Center (ADRC) of the National Institutes of Health. The work was funded by AVROBIO, Inc. Certain schematic elements were generated using BioRender.com.

AUTHOR CONTRIBUTIONS

Conceptualization, R.N.P., M.P.D., L.B., C.M., and N.P.v.T.; methodology, R.N.P., M.P.D., L.B., M.L., C.B., Y.D., Z.U., J.W.S., J.K.Y., F.H., Q.N., and T.P.; project administration, R.N.P. and M.P.D.; supervision, R.N.P., M.P.D., and L.B.; experimental execution, R.N.P., M.P.D., L.B., M.L., C.B., Y.D., Z.U., J.W.S., J.K.Y., F.H., Q.N., and T.P.; writing, R.N.P., M.P.D., and L.B.

DECLARATION OF INTERESTS

All authors are current employees of AVROBIO, Inc.

REFERENCES

1. Ferrari, G., Thrasher, A.J., and Aiuti, A. (2021). Gene therapy using haematopoietic stem and progenitor cells. Nat. Rev. Genet. *22*, 216–234. https://doi.org/10.1038/s41576-020-00298-5.
2. Morgan, R.A., Gray, D., Lomova, A., DB, K., and Kohn, D.B. (2017). Hematopoietic stem cell gene therapy: progress and lessons learned. Cell Stem Cell *21*, 574–590. https://doi.org/10.1016/j.stem.2017.10.010.
3. Dunbar, C.E., High, K.A., Joung, J.K., Kohn, D.B., Ozawa, K., and Sadelain, M. (2018). Gene therapy comes of age. Science *359*, eaan4672. https://doi.org/10.1126/science.aan4672.
4. Sagoo, P., and Gaspar, H.B. (2021). The transformative potential of HSC gene therapy as a genetic medicine. Gene Ther. https://doi.org/10.1038/s41434-021-00261-x.
5. De Ravin, S.S., Wu, X., Moir, S., Anaya-O'Brien, S., Anaya-O'Brien, S., Littel, P., Theobald, N., Choi, U., Su, L., Marquesen, M., et al. (2016). Lentiviral hematopoietic stem cell gene therapy for X-linked severe combined immunodeficiency. Sci. Transl. Med. *8*, 335ra57. https://doi.org/10.1126/scitranslmed.aad8856.
6. Aiuti, A., Roncarolo, M.G., and Naldini, L. (2017). Gene therapy for ADA-SCID, the first marketing approval of an ex vivo gene therapy in Europe: paving the road for the next generation of advanced therapy medicinal products. EMBO Mol. Med. *9*, 737–740. https://doi.org/10.15252/emmm.201707573.
7. Ferrua, F., Cicalese, M.P., Galimberti, S., Giannelli, S., Dionisio, F., Barzaghi, F., Migliavacca, M., Bernardo, M.E., Calbi, V., Assanelli, A.A., et al. (2019). Lentiviral haemopoietic stem/progenitor cell gene therapy for treatment of Wiskott-Aldrich syndrome: interim results of a non-randomised, open-label, phase 1/2 clinical study. Lancet Haematol. *6*, e239–e253. https://doi.org/10.1016/S2352-3026(19)30021-3.
8. Ribeil, J.A., Hacein-Bey-Abina, S., Payen, E., Magnani, A., Semeraro, M., Magrin, E., Caccavelli, L., Neven, B., Bourget, P., El Nemer, W., et al. (2017). Gene therapy in a patient with sickle cell disease. N. Engl. J. Med. *376*, 848–855. https://doi.org/10.1056/NEJMoa1609677.
9. Thompson, A.A., Walters, M.C., Kwiatkowski, J., Rasko, J.E.J., Ribeil, J.A., Hongeng, S., Magrin, E., Schiller, G.J., Payen, E., Semeraro, M., et al. (2018). Gene therapy in

patients with transfusion-dependent beta-thalassemia. N. Engl. J. Med. *378*, 1479–1493. https://doi.org/10.1056/NEJMoa1705342.

10. Taylor, M., Khan, S., Stapleton, M., Wang, J., Chen, J., Wynn, R., Yabe, H., Chinen, Y., Boelens, J.J., Mason, R.W., et al. (2019). Hematopoietic stem cell transplantation for mucopolysaccharidoses: past, present, and future. Biol. Blood Marrow Transpl. *25*, e226–e246. https://doi.org/10.1016/j.bbmt.2019.02.012.

11. Khan, A., Barber, D.L., Huang, J., Rupar, C.A., Rip, J.W., Auray-Blais, C., Boutin, M., O'Hoski, P., Gargulak, K., McKillop, W.M., et al. (2021). Lentivirus-mediated gene therapy for Fabry disease. Nat. Commun. *12*, 1178. https://doi.org/10.1038/s41467-021-21371-5.

12. Kohn, D.B., Booth, C., Kang, E.M., Pai, S.Y., Shaw, K.L., Santilli, G., Armant, M., Buckland, K.F., Choi, U., De Ravin, S.S., et al. (2020). Lentiviral gene therapy for X-linked chronic granulomatous disease. Nat. Med. *26*, 200–206. https://doi.org/10.1038/s41591-019-0735-5.

13. Kohn, D.B., Booth, C., Shaw, K.L., Xu-Bayford, J., Garabedian, E., Trevisan, V., Carbonaro-Sarracino, D.A., Soni, K., Terrazas, D., Snell, K., et al. (2021). Autologous ex vivo lentiviral gene therapy for adenosine deaminase deficiency. N. Engl. J. Med. *384*, 2002–2013. https://doi.org/10.1056/NEJMoa2027675.

14. Dahl, M., Doyle, A., Olsson, K., Månsson, J.E., Marques, A.R.A., Mirzaian, M., Aerts, J.M., Ehinger, M., Rothe, M., Modlich, U., et al. (2015). Lentiviral gene therapy using cellular promoters cures type 1 Gaucher disease in mice. Mol. Ther. *23*, 835–844. https://doi.org/10.1038/mt.2015.16.

15. Peterson, C.W., Adair, J.E., Wohlfahrt, M.E., Deleage, C., Radtke, S., Rust, B., Norman, K.K., Norgaard, Z.K., Schefter, L.E., Sghia-Hughes, G.M., et al. (2019). Autologous, gene-modified hematopoietic stem and progenitor cells repopulate the central nervous system with distinct clonal variants. Stem Cell Rep. *13*, 91–104. https://doi.org/10.1016/j.stemcr.2019.05.016.

16. Wolf, N.I., Breur, M., Plug, B., Beerepoot, S., Westerveld, A.S.R., van Rappard, D.F., de Vries, S.I., Kole, M.H.P., Vanderver, A., van der Knaap, M.S., et al. (2020). Metachromatic leukodystrophy and transplantation: remyelination, no cross-correction. Ann. Clin. Transl. Neurol. *7*, 169–180. https://doi.org/10.1002/acn3.50975.

17. Koelle, S.J., Espinoza, D.A., Wu, C., Xu, J., Lu, R., Li, B., Donahue, R.E., and Dunbar, C.E. (2017). Quantitative stability of hematopoietic stem and progenitor cell clonal output in rhesus macaques receiving transplants. Blood *129*, 1448–1457. https://doi.org/10.1182/blood-2016-07-728691.

18. Capotondo, A., Milazzo, R., Garcia-Manteiga, J.M., Cavalca, E., Montepeloso, A., Garrison, B.S., Peviani, M., Rossi, D.J., and Biffi, A. (2017). Intracerebroventricular delivery of hematopoietic progenitors results in rapid and robust engraftment of microglia-like cells. Sci. Adv. *3*, e1701211. https://doi.org/10.1126/sciadv.1701211.

19. Biffi, A., Montini, E., Lorioli, L., Cesani, M., Fumagalli, F., Plati, T., Baldoli, C., Martino, S., Calabria, A., Canale, S., et al. (2013). Lentiviral hematopoietic stem cell gene therapy benefits metachromatic leukodystrophy. Science *341*, 1233158. https://doi.org/10.1126/science.1233158.

20. Sessa, M., Lorioli, L., Fumagalli, F., Acquati, S., Redaelli, D., Baldoli, C., Canale, S., Lopez, I.D., Morena, F., Calabria, A., et al. (2016). Lentiviral haemopoietic stem-cell gene therapy in early-onset metachromatic leukodystrophy: an ad-hoc analysis of a non-randomised, open-label, phase 1/2 trial. Lancet *388*, 476–487. https://doi.org/10.1016/S0140-6736(16)30374-9.

21. Eichler, F., Duncan, C., Musolino, P.L., Orchard, P.J., De Oliveira, S., Thrasher, A.J., Armant, M., Dansereau, C., Lund, T.C., Miller, W.P., et al. (2017). Hematopoietic stem-cell gene therapy for cerebral adrenoleukodystrophy. N. Engl. J. Med. *377*, 1630–1638. https://doi.org/10.1056/NEJMoa1700554.

22. Stok, M., de Boer, H., Huston, M.W., Jacobs, E.H., Roovers, O., Visser, T.P., Jahr, H., Duncker, D.J., van Deel, E.D., Reuser, A.J.J., et al. (2020). Lentiviral hematopoietic stem cell gene therapy corrects murine pompe disease. Mol. Ther. Methods Clin. Dev. *17*, 1014–1025. https://doi.org/10.1016/j.omtm.2020.04.023.

23. Gleitz, H.F., Liao, A.Y., Cook, J.R., Rowlston, S.F., Forte, G.M., D'Souza, Z., O'Leary, C., Holley, R.J., and Bigger, B.W. (2018). Brain-targeted stem cell gene therapy corrects mucopolysaccharidosis type II via multiple mechanisms. EMBO Mol. Med. *10*, e8730. https://doi.org/10.15252/emmm.201708730.

24. Visigalli, I., Delai, S., Politi, L.S., Di Domenico, C., Cerri, F., Mrak, E., D'Isa, R., Ungaro, D., Stok, M., Sanvito, F., et al. (2010). Gene therapy augments the efficacy of hematopoietic cell transplantation and fully corrects mucopolysaccharidosis type I phenotype in the mouse model. Blood *116*, 5130–5139. https://doi.org/10.1182/blood-2010-04-278234.

25. Langford-Smith, A., Wilkinson, F.L., Langford-Smith, K.J., Holley, R.J., Sergijenko, A., Howe, S.J., Bennett, W.R., Jones, S.A., Wraith, J., Merry, C.L., et al. (2012). Hematopoietic stem cell and gene therapy corrects primary neuropathology and behavior in mucopolysaccharidosis IIIA mice. Mol. Ther. *20*, 1610–1621. https://doi.org/10.1038/mt.2012.82.

26. Holley, R.J., Ellison, S.M., Fil, D., O'Leary, C., McDermott, J., Senthivel, N., Langford-Smith, A.W.W., Wilkinson, F.L., D'Souza, Z., Parker, H., et al. (2018). Macrophage enzyme and reduced inflammation drive brain correction of mucopolysaccharidosis IIIB by stem cell gene therapy. Brain *141*, 99–116. https://doi.org/10.1093/brain/awx311.

27. Biffi, A., Capotondo, A., Fasano, S., Carro, U.d., Marchesini, S., Azuma, H., Malaguti, M.C., Amadio, S., Brambilla, R., Grompe, M., et al. (2006). Gene therapy of metachromatic leukodystrophy reverses neurological damage and deficits in mice. J. Clin. Invest. *116*, 3070–3082. https://doi.org/10.1172/JCI28873.

28. Cartier, N., Hacein-Bey-Abina, S., Bartholomae, C.C., Veres, G., Schmidt, M., Kutschera, I., Vidaud, M., Abel, U., Dal-Cortivo, L., Caccavelli, L., et al. (2009). Hematopoietic stem cell gene therapy with a lentiviral vector in X-linked adrenoleukodystrophy. Science *326*, 818–823. https://doi.org/10.1126/science.1171242.

29. Shemer, A., Grozovski, J., Tay, T.L., Tao, J., Volaski, A., Süß, P., Ardura-Fabregat, A., Gross-Vered, M., Kim, J.S., David, E., et al. (2018). Engrafted parenchymal brain macrophages differ from microglia in transcriptome, chromatin landscape and response to challenge. Nat. Commun. *9*, 5206. https://doi.org/10.1038/s41467-018-07548-5.

30. Jordão, M.J.C., Sankowski, R., Brendecke, S.M., Sagar Locatelli, G., Tai, Y.H., Tay, T.L., Schramm, E., Armbruster, S., Hagemeyer, N., Gross, O., Groß, O., Groß, O., Mai, D., Çiçek, Ö., Çiçek, Ö., Falk, T., Kerschensteiner, M., Grün, D., Grün, D., and Prinz, M. (2019). Single-cell profiling identifies myeloid cell subsets with distinct fates during neuroinflammation. Science *363*, eaat7554. https://doi.org/10.1126/science.aat7554.

31. Hohsfield, L.A., Najafi, A.R., Ghorbanian, Y., Soni, N., Hingco, E.E., Kim, S.J., Jue, A.D., Swarup, V., Inlay, M.A., and Green, K.N. (2020). Effects of long-term and brain-wide colonization of peripheral bone marrow-derived myeloid cells in the CNS. J. Neuroinflammation *17*, 279. https://doi.org/10.1186/s12974-020-01931-0.

32. Wang, P.L., Yim, A.K.Y., Kim, K.W., Avey, D., Czepielewski, R.S., Colonna, M., Milbrandt, J., and Randolph, G.J. (2020). Peripheral nerve resident macrophages share tissue-specific programming and features of activated microglia. Nat. Commun. *11*, 2552. https://doi.org/10.1038/s41467-020-16355-w.

33. Haage, V., Semtner, M., Vidal, R.O., Hernandez, D.P., Pong, W.W., Chen, Z., Hambardzumyan, D., Magrini, V., Ly, A., Walker, J., et al. (2019). Comprehensive gene expression meta-analysis identifies signature genes that distinguish microglia from peripheral monocytes/macrophages in health and glioma. Acta Neuropathol. Commun. *7*, 20. https://doi.org/10.1186/s40478-019-0665-y.

34. Van Hove, H., Martens, L., Scheyltjens, I., De Vlaminck, K., Pombo Antunes, A.R., De Prijck, S., Vandamme, N., De Schepper, S., Van Isterdael, G., Scott, C.L., et al. (2019). A single-cell atlas of mouse brain macrophages reveals unique transcriptional identities shaped by ontogeny and tissue environment. Nat. Neurosci. *22*, 1021–1035. https://doi.org/10.1038/s41593-019-0393-4.

35. Hammond, T.R., Dufort, C., Dissing-Olesen, L., Giera, S., Young, A., Wysoker, A., Walker, A.J., Gergits, F., Segel, M., Nemesh, J., et al. (2019). Single-cell RNA sequencing of microglia throughout the mouse lifespan and in the injured brain reveals complex cell-state changes. Immunity *50*, 253–271.e6. https://doi.org/10.1016/j.immuni.2018.11.004.

36. O'Regan, G., deSouza, R.M., Balestrino, R., and Schapira, A.H. (2017). Glucocerebrosidase mutations in Parkinson disease. J. Parkinsons Dis. *7*, 411–422. https://doi.org/10.3233/JPD-171092.

37. Jesús, S., Huertas, I., Bernal-Bernal, I., Bonilla-Toribio, M., Cáceres-Redondo, M.T., Vargas-González, L., Gómez-Llamas, M., Carrillo, F., Calderón, E., Carballo, M., et al. (2016). GBA variants influence motor and non-motor features of Parkinson's disease. PLoS One *11*, e0167749. https://doi.org/10.1371/journal.pone.0167749.

38. Nalls, M.A., Duran, R., Lopez, G., Kurzawa-Akanbi, M., McKeith, I.G., Chinnery, P.F., Morris, C.M., Theuns, J., Crosiers, D., Cras, P., et al. (2013). A multicenter study of glucocerebrosidase mutations in dementia with Lewy bodies. JAMA Neurol. *70*, 727. https://doi.org/10.1001/jamaneurol.2013.1925.

39. Polinski, N.K., Martinez, T.N., Gorodinsky, A., Gareus, R., Sasner, M., Herberth, M., Switzer, R., Ahmad, S.O., Cosden, M., Kandebo, M., et al. (2021). Decreased glucocerebrosidase activity and substrate accumulation of glycosphingolipids in a novel GBA1 D409V knock-in mouse model. PLoS One *16*, e0252325. https://doi.org/10.1371/journal.pone.0252325.

40. Greaves, C.V., and Rohrer, J.D. (2019). An update on genetic frontotemporal dementia. J. Neurol. *266*, 2075–2086. https://doi.org/10.1007/s00415-019-09363-4.

41. Nguyen, A.D., Nguyen, T.A., Zhang, J., Devireddy, S., Zhou, P., Karydas, A.M., Xu, X., Miller, B.L., Rigo, F., Ferguson, S.M., et al. (2018). Murine knockin model for progranulin-deficient frontotemporal dementia with nonsense-mediated mRNA decay. Proc. Natl. Acad. Sci. U S A *115*, E2849–E2858. https://doi.org/10.1073/pnas.1722344115.

42. Yang, Y., Aloi, M.S., Cudaback, E., Josephsen, S.R., Rice, S.J., Jorstad, N.L., Keene, C.D., and Montine, T.J. (2014). Wild-type bone marrow transplant partially reverses neuroinflammation in progranulin-deficient mice. Lab Invest. *94*, 1224–1236. https://doi.org/10.1038/labinvest.2014.113.

43. Chang, M.C., Srinivasan, K., Friedman, B.A., Suto, E., Modrusan, Z., Lee, W.P., Kaminker, J.S., Hansen, D.V., and Sheng, M. (2017). Progranulin deficiency causes impairment of autophagy and TDP-43 accumulation. J. Exp. Med. *214*, 2611–2628. https://doi.org/10.1084/jem.20160999.

44. Arrant, A.E., Onyilo, V.C., Unger, D.E., and Roberson, E.D. (2018). Progranulin gene therapy improves lysosomal dysfunction and microglial pathology associated with frontotemporal dementia and neuronal ceroid lipofuscinosis. J. Neurosci. *38*, 2341–2358. https://doi.org/10.1523/JNEUROSCI.3081-17.2018.

45. Tayebi, N., Lopez, G., Do, J., and Sidransky, E. (2020). Pro-cathepsin D, prosaposin, and progranulin: lysosomal networks in parkinsonism. Trends Mol. Med. *26*, 913–923. https://doi.org/10.1016/j.molmed.2020.07.004.

46. Capotondo, A., Milazzo, R., Politi, L.S., Quattrini, A., Palini, A., Plati, T., Merella, S., Nonis, A., di Serio, C., Montini, E., et al. (2012). Brain conditioning is instrumental for successful microglia reconstitution following hematopoietic stem cell transplantation. Proc. Natl. Acad. Sci. U S A *109*, 15018–15023. https://doi.org/10.1073/pnas.1205858109.

47. Tucci, F., Scaramuzza, S., Aiuti, A., and Mortellaro, A. (2021). Update on clinical ex vivo hematopoietic stem cell gene therapy for inherited monogenic diseases. Mol. Ther. *29*, 489–504. https://doi.org/10.1016/j.ymthe.2020.11.020.

48. Liu, F., Dai, S., Feng, D., Qin, Z., Peng, X., Sakamuri, S.S.V.P., Ren, M., Huang, L., Cheng, M., Mohammad, K.E., et al. (2020). Distinct fate, dynamics and niches of renal macrophages of bone marrow or embryonic origins. Nat. Commun. *11*, 2280. https://doi.org/10.1038/s41467-020-16158-z.

49. Li, F., Okreglicka, K.M., Pohlmeier, L.M., Schneider, C., and Kopf, M. (2020). Fetal monocytes possess increased metabolic capacity and replace primitive macrophages in tissue macrophage development. EMBO J. *39*, e103205. https://doi.org/10.15252/embj.2019103205.

50. Merlin, S., Bhargava, K.K., Ranaldo, G., Zanolini, D., Palestro, C.J., Santambrogio, L., Prat, M., Follenzi, A., and Gupta, S. (2016). Kupffer cell transplantation in mice for elucidating monocyte/macrophage biology and for potential in cell or gene therapy. Am. J. Pathol. *186*, 539–551. https://doi.org/10.1016/j.ajpath.2015.11.002.

51. van de Laar, L., Saelens, W., De Prijck, S., Martens, L., Scott, C.L., Van Isterdael, G., Hoffmann, E., Beyaert, R., Saeys, Y., Lambrecht, B.N., and Guilliams, M. (2016). Yolk sac macrophages, fetal liver, and adult monocytes can colonize an empty niche and develop into functional tissue-resident macrophages. Immunity *44*, 755–768. https://doi.org/10.1016/j.immuni.2016.02.017.

52. Zhu, Y., Ohama, T., Kawase, R., Chang, J., Inui, H., Kanno, K., Okada, T., Masuda, D., Koseki, M., Nishida, M., et al. (2020). Progranulin deficiency leads to enhanced age-related cardiac hypertrophy through complement C1q-induced β-catenin activation. J. Mol. Cell. Cardiol. *138*, 197–211. https://doi.org/10.1016/j.yjmcc.2019.12.009.

53. Poewe, W., Seppi, K., Tanner, C., Halliday, G., Brundin, P., Volkmann, J., Schrag, A., and Lang, A. (2017). Nature reviews. Dis. Primers *3*, 17013. https://doi.org/10.1038/nrdp.2017.13.

54. Arrant, A.E., Filiano, A.J., Unger, D.E., Young, A.H., and Roberson, E.D. (2017). Restoring neuronal progranulin reverses deficits in a mouse model of frontotemporal dementia. Brain *140*, 1447–1465. https://doi.org/10.1093/brain/awx060.

55. Samaranch, L., Blits, B., San Sebastian, W., Hadaczek, P., Bringas, J., Sudhakar, V., Macayan, M., Pivirotto, P.J., Petry, H., and Bankiewicz, K.S. (2017). MR-guided parenchymal delivery of adeno-associated viral vector serotype 5 in non-human primate brain. Gene Ther. *24*, 253–261. https://doi.org/10.1038/gt.2017.14.

56. Bohlen, M.O., McCown, T.J., Powell, S.K., El-Nahal, H.G., Daw, T., Basso, M.A., Sommer, M.A., and Samulski, R.J. (2020). Adeno-associated virus capsid-promoter interactions in the brain translate from rat to the nonhuman primate. Hum. Gene Ther. *31*, 1155–1168. https://doi.org/10.1089/hum.2020.196.

57. Rocha, E.M., Smith, G.A., Park, E., Cao, H., Brown, E., Hayes, M.A., Beagan, J., McLean, J.R., Izen, S.C., Perez-Torres, E., et al. (2015). Glucocerebrosidase gene therapy prevents alpha-synucleinopathy of midbrain dopamine neurons. Neurobiol. Dis. *82*, 495–503. https://doi.org/10.1016/j.nbd.2015.09.009.

58. Sailor, K.A., Agoranos, G., López-Manzaneda, S., Tada, S., Gillet-Legrand, B., Guerinot, C., Masson, J.B., Vestergaard, C.L., Bonner, M., Gagnidze, K., et al. (2022). Hematopoietic stem cell transplantation chemotherapy causes microglia senescence and peripheral macrophage engraftment in the brain. Nat. Med. *28*, 517–527. https://doi.org/10.1038/s41591-022-01691-9.

59. Shibuya, Y., Kumar, K.K., Mader, M.M.D., Yoo, Y., Ayala, L.A., Zhou, M., Mohr, M.A., Neumayer, G., Kumar, I., Yamamoto, R., et al. (2022). Treatment of a genetic brain disease by CNS-wide microglia replacement. Sci. Transl. Med. *14*, eabl9945. https://doi.org/10.1126/scitranslmed.abl9945.

60. Berger, Z., Perkins, S., Ambroise, C., Oborski, C., Calabrese, M., Noell, S., Riddell, D., and Hirst, W.D. (2015). Tool compounds robustly increase turnover of an artificial substrate by glucocerebrosidase in human brain lysates. PLoS One *10*, e0119141. https://doi.org/10.1371/journal.pone.0119141.

61. Sanyal, A., DeAndrade, M.P., Novis, H.S., Lin, S., Chang, J., Lengacher, N., Tomlinson, J.J., Tansey, M.G., and LaVoie, M.J. (2020). Lysosome and inflammatory defects in GBA1-mutant astrocytes are normalized by LRRK2 inhibition. Mov Disord. *35*, 760–773. https://doi.org/10.1002/mds.27994.

YMTHE, Volume *30*

Supplemental Information

High-throughput analysis of hematopoietic stem cell engraftment after intravenous and intracerebroventricular dosing

Robert N. Plasschaert, Mark P. DeAndrade, Fritz Hull, Quoc Nguyen, Tara Peterson, Aimin Yan, Mariana Loperfido, Cristina Baricordi, Luigi Barbarossa, John K. Yoon, Yildirim Dogan, Zeenath Unnisa, Jeffrey W. Schindler, Niek P. van Til, Luca Biasco, and Chris Mason

SUPPLEMENTARY METHODS

SDS-PAGE of beta glucocerebrosidase, progranulin, and Cathepsin D

For RAW264.7 cells, 2.5 µg of protein lysate was mixed with 4x protein sample buffer (XT Sample Buffer, Bio-Rad) and water to a volume of 15 µL. The samples were heated at 95°C for 5 minutes. Samples were loaded into precast 10% Bis-Tris polyacrylamide gels (Bio-Rad) with MOPS buffer (Bio-Rad) and electrophoresed. For brain lysates, 10 µg of protein lysate was mixed with 4x protein sample buffer (Invitrogen or Bio-Rad) and water or lysis buffer to a volume of 15 µL. The samples were heated at 70-95°C for 5-10 minutes. Samples were loaded into precast 4-20% Tris-HCl polyacrylamide gels (Bio-Rad) with Tris/Glycine/SDS buffer (Bio-Rad) and electrophoresed. After completion of electrophoresis, gels were transferred to PVDF membranes using the iBlot 2 system (ThermoFisher). PVDF membranes were blocked for 1 hour with 5% BSA in 0.05% PBS-Tween. Primary antibodies (GCase, progranulin, cathepsin D, beta-actin) were diluted in 5% BSA in 0.05% PBS-Tween and incubated with PVDF membranes overnight at 4°C. PVDF membranes were washed three times with 0.05% PBS-Tween and then incubated with appropriate HRP secondary antibodies (Bio-Rad) for 1 hour at 1:50,000 dilution in 5% BSA in 0.05% PBS-Tween. Blots were washed three times in 0.05% PBS-Tween for 10 minutes each and then incubated with chemiluminescence reagent (SuperSignal West Femto, ThermoFisher). Images were acquired using a charge-coupled camera (G:BOX, Syngene). Bands were quantified using ImageJ.

Transduction of immortalized cell lines

RAW264.7 mouse macrophage cells (ATCC; confirmed by STR profiling, IDEXX) were transduced with increasing multiplicity of infections with LVV.GRN or LVV.GBA vector. Approximately 85,000 cells per well of a 6-well dish were seeded 24 hours prior to transduction in 2 mL of complete growth media (DMEM supplemented with 10% heat-inactivated FBS, pyruvate, GlutaMAX, and penicillin/streptomycin). Transduction media was removed after 16 hours and replaced with fresh complete growth media. Cells were collected 4 days post-transduction, washed with DPBS twice, pelleted by centrifugation at 500xg for 5 min, and frozen at -80°C prior to downstream molecular characterization.

Flow cytometry analysis for myeloid and lymphoid populations

For the blood and bone marrow samples, red blood cells were lysed and resuspended in RPMI medium. White blood cells were split into aliquots. The first aliquot was stained with CD45.1-APC (clone A20), CD45.2-PE (clone 104), CD3ε-PerCP-Cy5.5 (clone 17A2), and B220-APC-Cy7 (clone RA3-6B2) for 20 minutes at 4°C. The second aliquot was stained with CD45.1-APC (clone RB6-8C5), CD45.2-PE (clone 104), TER119-PerCP-Cy5.5, CD11b-PE-Cy7 (M1/70), GR1-APC-Cy7 (clone RB6-8C5) for 20 minutes at 4°C. After two washes, both aliquots were incubated with the viability marker Sytox Blue for 8 minutes at room temperature. Stained cells were measured using a MACSQuant Analyzer 10 (Miltenyi Biotec) and analyzed using FlowJo (FlowJo, LLC).

Quantification of GFP and Iba1 positive cells in the brain

Five sections across the rostral caudal axis of the brain (two sections from approximately +0.2 mm to +3.2 mm relative to bregma, two sections from approximately +0.2 mm to -4.06 mm relative to bregma, and one section covering the cerebellum from approximately -5.2 mm to -6.1 mm to bregma) were uploaded to the HALO platform (Indica Labs, version 3.1.1076.301) for analysis. A tissue classifying algorithm was trained to detect and select tissue from background, thereby creating a region of interest for subsequent analysis. Analysis outputs for each image set included positive signal area for both stains, colocalized positive signal area, cell count for each stain, and colocalized cell count.

Isolation of microglia/MLCs and single cell RNA-Seq

After transcardial perfusion, the brains of one male and female mice from IV and ICV groups were processed by enzymatic digestion (Neural Tissue Dissociation Kit (P), Miltenyi Biotec). The resulting single cell suspension was stained with viability dye and antibodies for microglia cells, neurons, astrocytes and endothelial cells (LIVE/DEAD Fixable Aqua Dead Cell Stain Kit, ThermoFisher; CD45.1 clone A20 in BV421 (BD); CD45.2 clone 104 in BV421 (BD); CD11b clone M1/70 in APC-780 (ThermoFisher); CX3CR1 clone SA011F11 in BV605 (BioLegend); PECy7 Thy1.1 (also known as CD90.1) clone OX-7 in PE-Cy7 (BD); Thy1.2 (also known as CD90.2) clone 53-2.1 in PE-Cy7 (BD);

ACSA-2 clone IH3-18A3 in PE (Miltenyi Biotec); CD31 clone 390 in PerCP/Cy5.5 (BioLegend). GFP-positve and GFP-negative fractions of microglia cells, defined as CD45+ CD11b+ Cx3cr1+, were FACS-sorted using a MA900 Multi-Application Cell Sorter (Sony Biotechnology). For the generation of single cell transcriptomes, $5x10^3$ target cells from each sorted population were run using the Chromium Controller (10x Genomics) using Chromium Next GEM Single Cell 3' Reagent Kits (10x Genomics). The libraries generated were then run on the NextSeq 550 Sequencing System (Illumina) using NextSeq 500/550 High Output v2.5 (150 cycles) Kit (Illumina). The Illumina raw BCL sequencing files were processed through the CellRanger software (10x Genomics) for generating FASTQ files and count matrixes (https://support.10xgenomics.com/single-cell-gene-expression/software/overview/welcome). The count matrixes were then used as input for the SEURAT V4.0 (https://satijalab.org/seurat/) and MONOCLE3 (http://cole-trapnell-lab.github.io/monocle-release/) R tools for single cell genomics analyses. Briefly, single cell barcodes were filtered for the ones containing mitochondrial gene content lower than 15%. Expression data then were normalized, scaled, and searched for variable features using the *SCTransform* function of SEURAT V4.0 followed by tSNE dimensionality reduction and clustering using the *FindClusters* function with resolution set at 0.2. For generating the maps shown in Fig.3b and Fig.4a we run the tSNE coordinates on the R package *ggplot2* (https://cran.r-project.org/web/packages/ggplot2/index.html). For visualization of gene expression shown in Fig.2b the SEURAT object was converted into a MONOCLE object and individual genes were plotted using the *plot_cells* function of MONOCLE3. The heatmaps of Fig. 3e, 4d and 5a were generated based on the average normalized gene expression per sample using the R package pheatmap (https://cran.r-project.org/web/packages/pheatmap/index.html).

Statistics

Statistical analysis was performed using either Prism (GraphPad) or R. For analyzing the relationship between vector volume and transgene metrics in Fig.6, we fit a linear regression model and measured the Pearson correlation and its statistical relevance (p-value). For scRNA-Seq data we used GSEAPreranked analysis to identify the gene sets that are enriched in GFP-negative and GFP-positive

samples. To perform this analysis, we firstly derived a score using log2FoldChange and p-value from differential gene expression analysis (score=-s*log10(p-value) with s=-1 if the log2FoldChange of gene <0, otherwise s=+1). We then sorted the genes based on the score value in decreasing order and used the whole ranked gene list file (including 11,992 genes and their score values) as input for the online tool WebGestalt (http://www.webgestalt.org) to perform GSEAPreranked analysis. We then generated a network-based enrichment map visualization for the 20 enriched gene sets. In this network, the edges correspond to the overlapping between connected gene sets and were calculated using the gene list of the leading-edge genes of each gene set from GSEA. The node size instead indicates the number of the leading-edge genes. The overlapping score was calculated using the following formula: $overlap = \frac{|A \cap B|}{\min(|A|,|B|)}$ where $|A \cap B|$ is the cardinality of the intersection between gene sets A and B while $|A|$ and $|B|$ are the cardinality of gene set A and gene set B, respectively. For the plot shown in Fig. 3D we applied the Procrustes tool from the R Package Vegan (https://cran.r-project.org/web/packages/vegan/vegan.pdf) to perform unsupervised clustering on the global gene*cell expression matrixes in different sample types. Since the number of cells for sample types are different, we downsampled all sample types to the smallest dataset and then calculated Procrustes distance for each sampled data sets and calculated and plotted the average from 10 random samplings. We then perform clustering using the averaged Procrustes distance. For all other data, a Student t-test (when only two groups were present), one-way ANOVA with Tukey's post hoc analysis (when multiple groups were present), or linear regression was used. Significance was defined as $p < 0.05$.

Immunofluorescence staining

For quantification of engraftment within the central nervous system, brains were paraffin embedded, sectioned at 20 µm, and 12 sections were stained for GFP and Iba1 and 12 sections were stained for GFP and TMEM119 at a 300 µm interval. Slides were scanned using VS200 Research Slide Scanner (Olympus). Images were uploaded and analyzed using the HALO platform (v3.1.1076.301, Indica Labs).

For qualitative analysis of GFP engraftment, peripheral tissues were fixed overnight in 4% PFA and then transferred to a solution of 30% sucrose until they sank to the bottom. Subsequently, tissues were mounted in tissue freezing media and stored at -80°C. Tissues were sectioned at 20-50 μm (depending on tissue) using a sliding microtome with a frozen stage (ThermoFisher) or cryostat (Leica). Sections were mounted on glass slides using a glycerol-based mounting media.

For progranulin IHC, brains were processed similar to peripheral tissues. Sections were first incubated with an antigen retrieval solution (Antigen Retrieval Reagent-Basic, R&D Systems) at 80°C for 30 minutes followed by blocking (2% non-fat dry milk, 0.3% Triton X-100, and 0.01% sodium azide in PBS). Next, sections were blocked again in 1% donkey serum in 0.3% Triton X-100 in PBS for 1hr at room temperature. Sections were then incubated with primary antibodies for progranulin (AF2420 R&D Systems) and NeuN (MAB377 MilliporeSigma) overnight at 4°C followed by the appropriate fluorescent secondary antibodies for one hour.

Supplemental Table 1. Primers and probe sequences

Target	Purpose	Primer	Sequence (5'-3')	Vendor
WPRE (v1)	VCN, Expression	Forward	TTCTGGGACTTTCGCTTTCC	IDT
WPRE (v1)	VCN, Expression	Reverse	CCGACAACACCACGGAATTA	IDT
WPRE (v1)	VCN, Expression	Probe	ATCGCCACGGCAGAACTCATCG	IDT
Tfrc	VCN	Forward	Not available	ThermoFisher, 4458367
Tfrc	VCN	Reverse	Not available	ThermoFisher, 4458367
Tfrc	VCN	Probe	Not available	ThermoFisher, 4458367
Gtdc1	VCN	Forward	GAAGTTCAGGTTAATTAGCTGCTG	IDT
Gtdc1	VCN	Reverse	GGCACCTTAACATTTGGTTCTG	IDT
Gtdc1	VCN	Probe	ACGAACTTCTTGGAGTTGTTTGCT	IDT
Actb	Expression	Forward	Not available	ThermoFisher, Mm02619580_g1
Actb	Expression	Reverse	Not available	ThermoFisher, Mm02619580_g1
Actb	Expression	Probe	Not available	ThermoFisher, Mm02619580_g1
WPRE (v2)	Expression	Forward	GGCTTTCRTTTTCTCCTCCTTGTAT	ThermoFisher
WPRE (v2)	Expression	Reverse	CGGGCCACAACTCCTCATAA	ThermoFisher
WPRE (v2)	Expression	Probe	AATCCTGGTTGCTGTCTC	ThermoFisher
Psi element	VCN	Forward	CAGGACTCGGCTTGCTGAAG	IDT
Psi element	VCN	Reverse	TCCCCCGCTTAATACTGACG	IDT
Psi element	VCN	Probe	CGCACGGCAAGAGGCGAGG	IDT

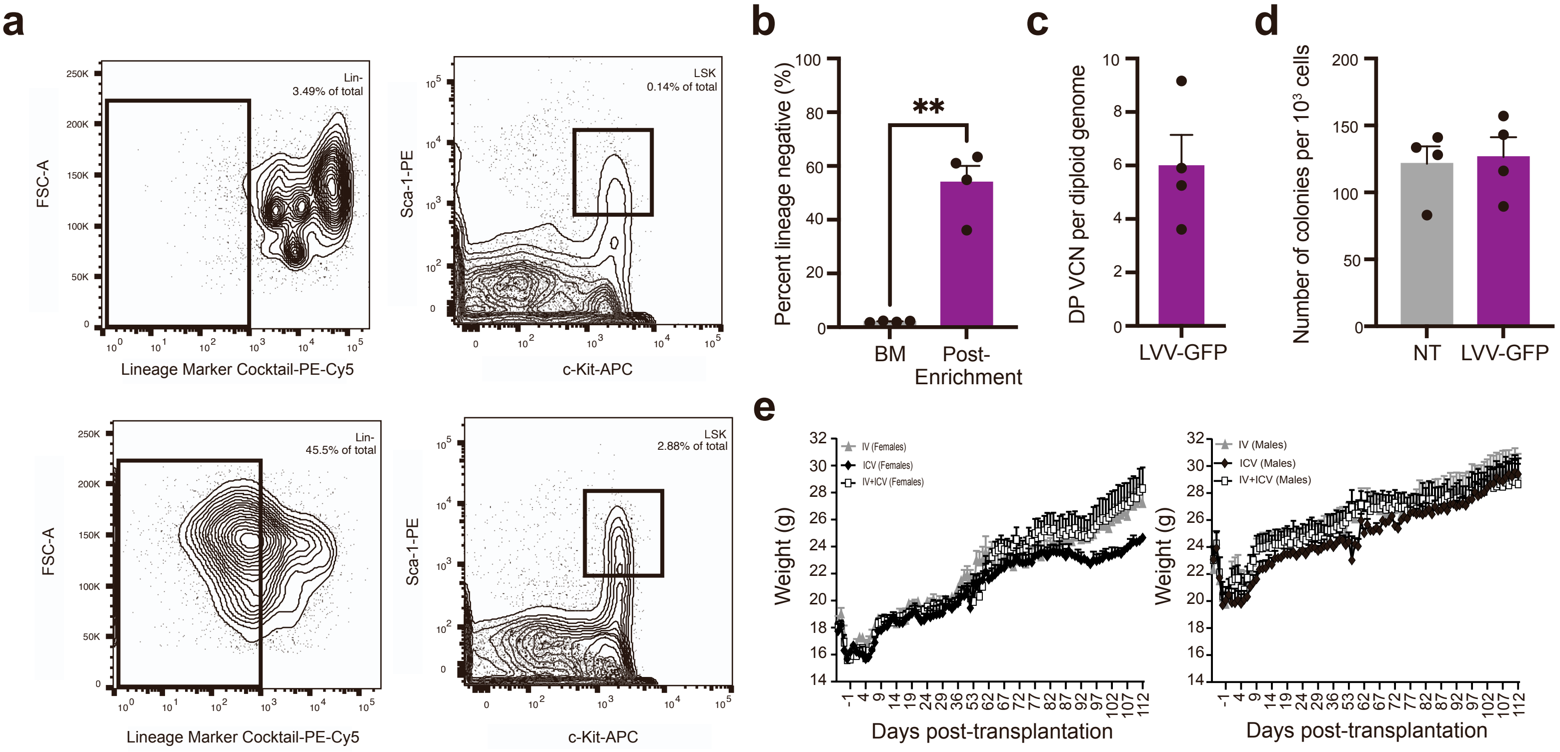

Supplementary Figure 1. Drug product characterization and post-transplantation body weights.
a Representative flow cytometry analysis plots of total bone marrow (top) and lineage negative cells after enrichment (bottom) for lineage markers (left panels) and c-Kit/Sca1 (right panels). **b** Quantification of lineage negative population in four drug product preprarations. **c** Vector copy number per diploid genome in the drug product. **d** Colony forming unit assay to assess pluripotency of lineage negative cells. **e** Body weights of female (left) and male (right) animals through the course of the study. LSK=Lineage negative, Sca1+, c-Kit+. T-test was used for statistical analysis. **, p<0.01. Bars represent means ± SEM.

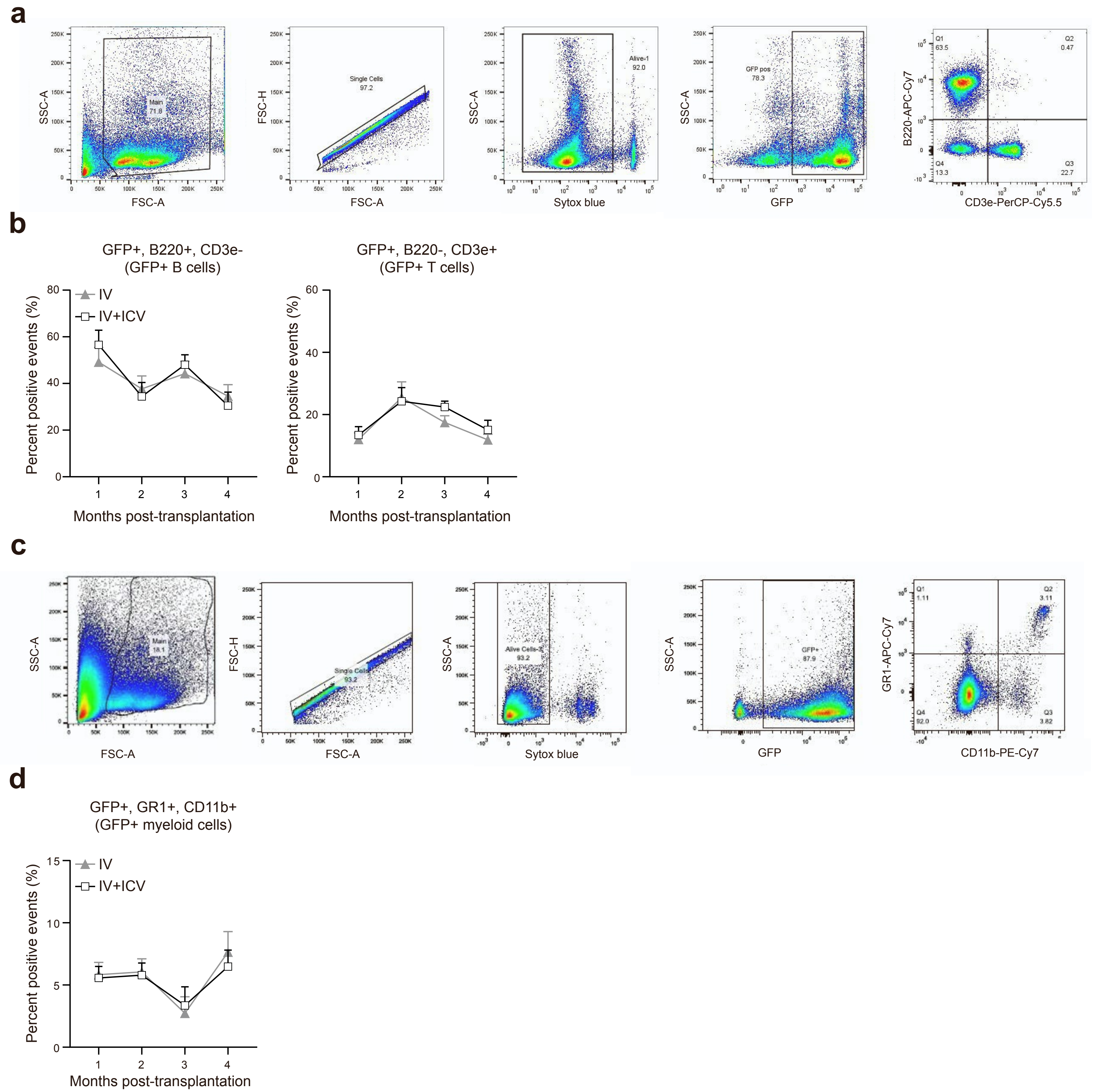

Supplementary Figure 2. Flow cytometry analysis of peripheral blood to measure engraftment in various cell compartments. a-b Representative flow cytometry plots and quantitation of GFP-positive (GFP+) B cells (B220+, CD3e-) and T cells (B220-, CD3e+) in IV and IV+ICV animals. **c-d** Representative flow cytometry plots and quantitation of GFP-positive myeloid cells (GR1+, CD11b+) in IV and IV+ICV animals. Symbols represent means ± SEM.

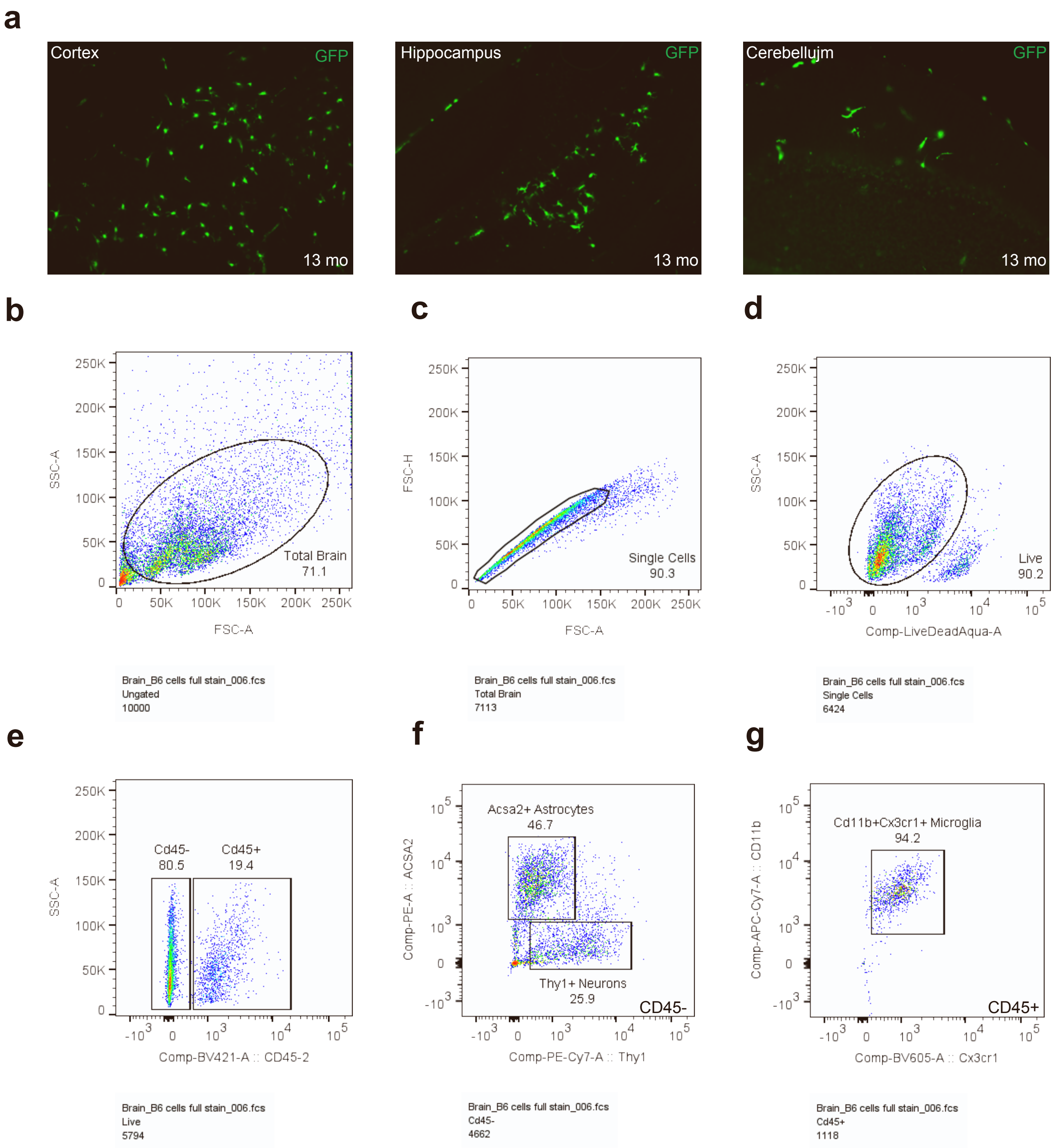

Supplementary Figure 3. Representative images of engraftment at 13 months post-treatment and gating strategy for FACS isolation of microglia, astrocytes, and neurons. (a) Representative images of GFP-positive cells in the cortex, hippocampus, and cerebellum at 13 months post-treatment. Whole adult mouse brains were enzymatically digested to single cell suspension and then stained with cell-surface marker antibodies to enable FACS-assisted isolation of specific cell populations. Representative gating strategy shows the selection of the single cell population **(b,c)**, selection for viability **(d)**, gating on the immune cell marker CD45 **(e)** selection of CD45- ACSA2+, Thy1- astrocytes, and CD45- ACSA2- Thy1+ neurons **(f)**, selection of CD45+ CD11b+, C3X3CR1+ microglia **(g)**. Percentages of parent population are listed next to each gate.

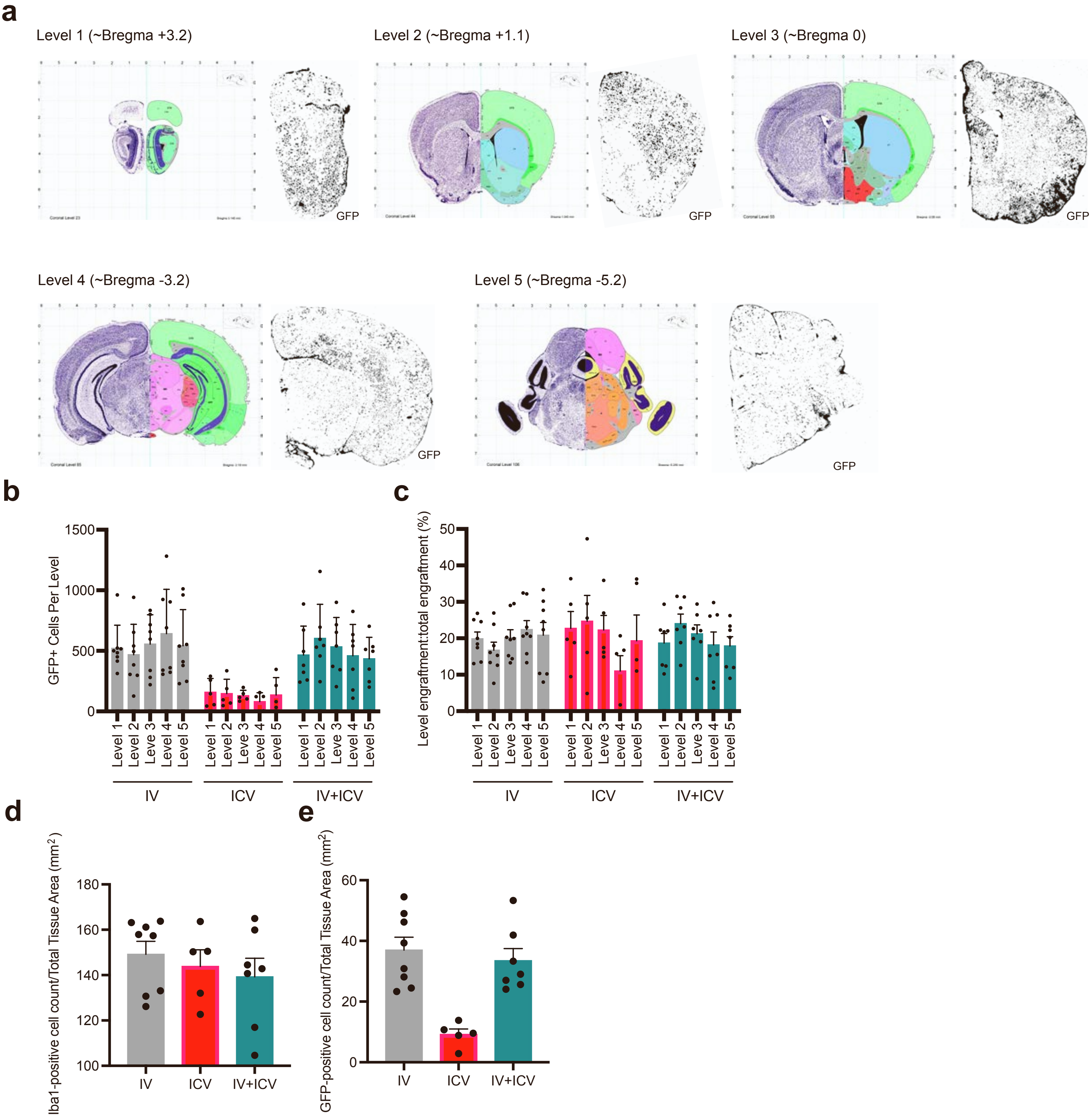

Supplementary Figure 4. Engraftment distribution and density in the brain. (a) Representative reference atlas images (from the Allen Mouse Brain Atlas and Allen Reference Atlas - Mouse Brain) and sudy images of GFP+ cells (threshold applied in ImageJ to remove background) for each level analyzed. (b) Number of GFP+ cells counted for each level analyzed. (c) Number of GFP+ cells for each level normalized to the total number of GFP+ cells found in the brain. (d) Number of Iba1+ cells per mm^2. (e) Number of GFP+ cells per mm^2.

Allen Institute for Brain Science (2004). Allen Mouse Brain Atlas [dataset]. Available from mouse.brain-map.org.Allen Institute for Brain Science (2011).
Allen Reference Atlas – Mouse Brain [brain atlas]. Available from atlas.brain-map.org.

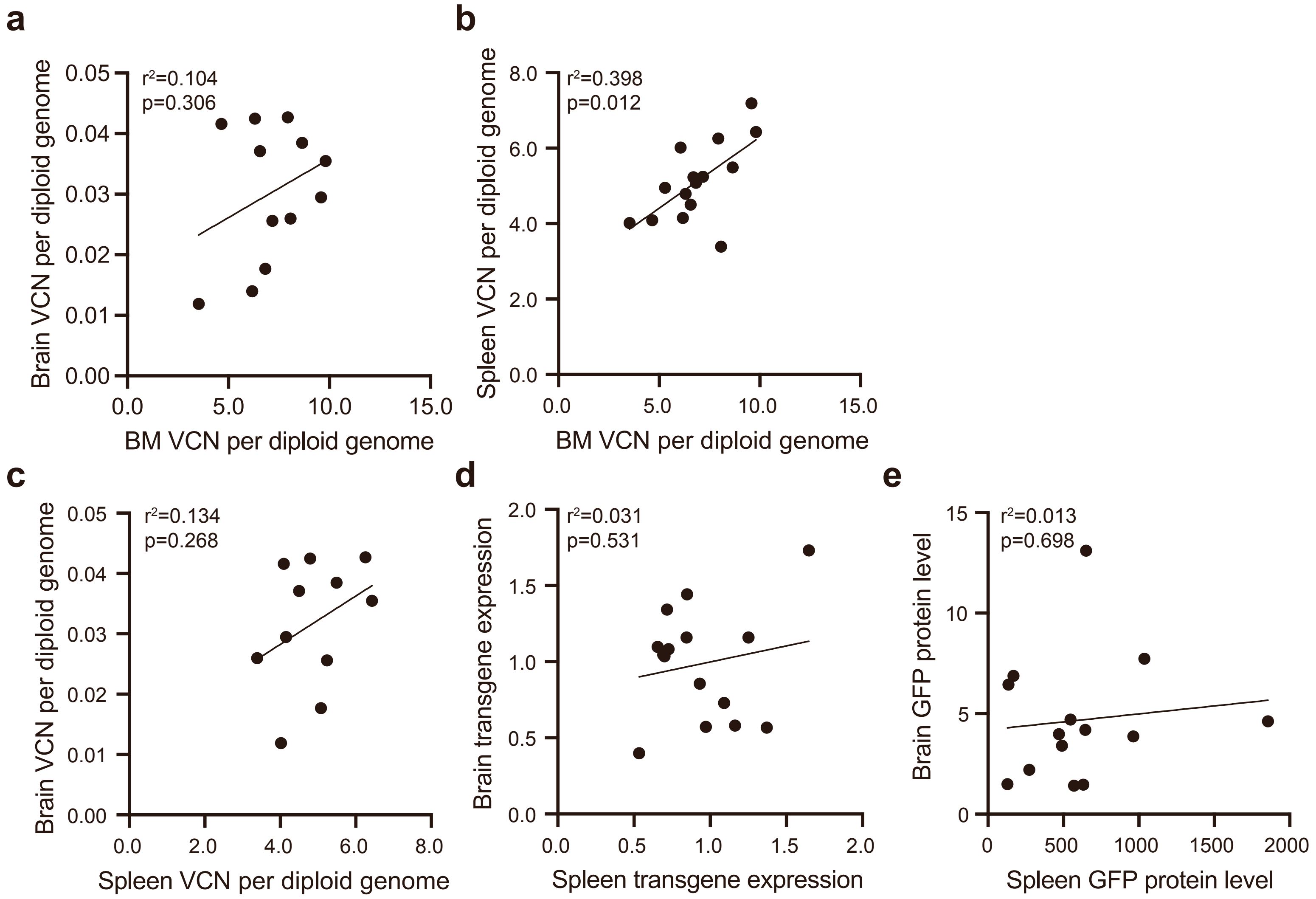

Supplementary Figure 5. Linear regression analysis of brain, bone marrow, and spleen biodistribution metrics from animals dosed IV or IV+ICV.

a Brain vector copy number (VCN) versus bone marrow (BM) VCN per diploid genome. **b** Spleen VCN versus BM VCN. **c** Brain VCN versus spleen VCN. **d** Brain transgene expression vs spleen transgene expression**. e** Brain GFP protein versus spleen GFP protein. Line and statistics represent linear fit after simple linear regression.

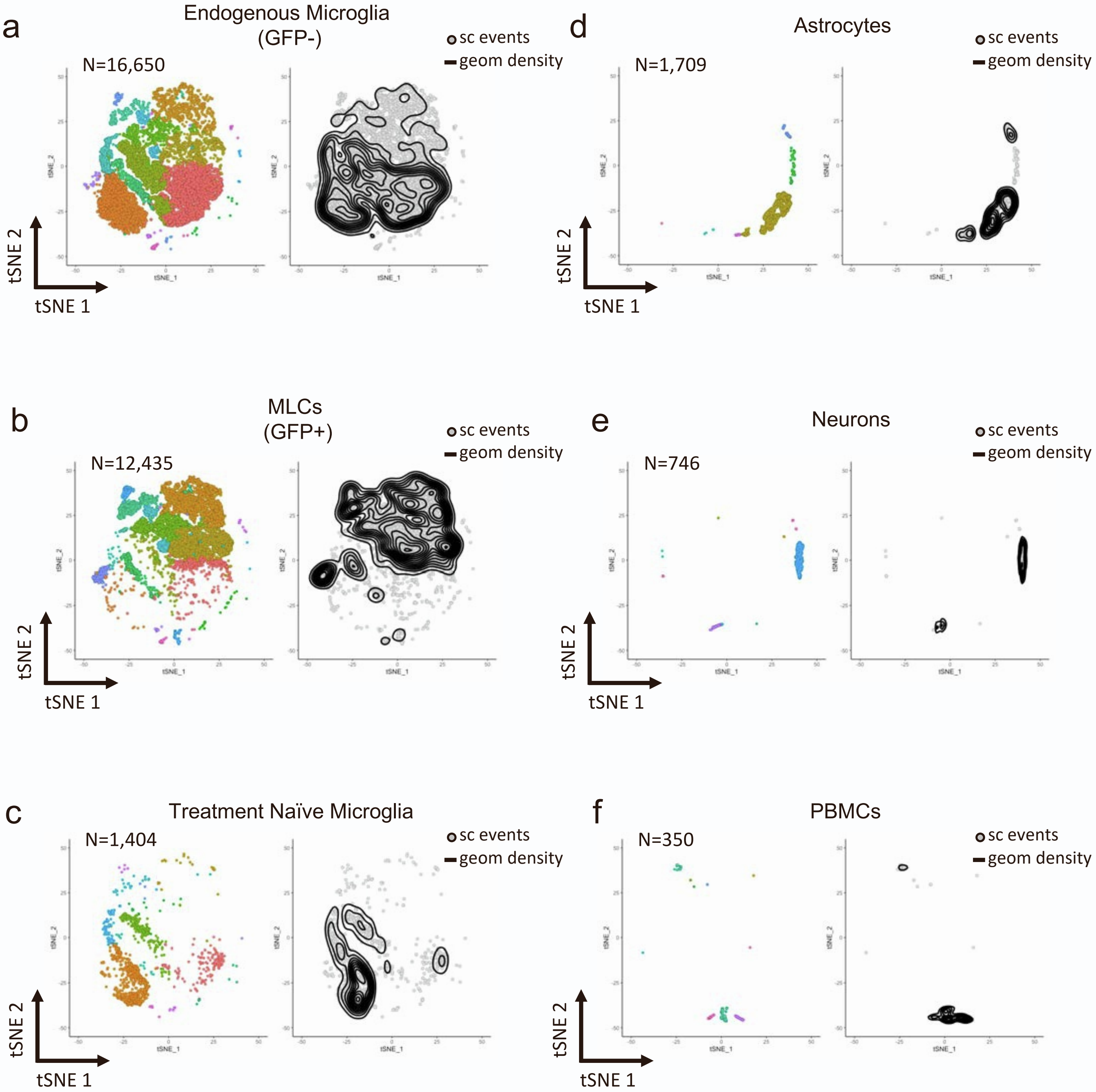

Supplemental Figure 6. tSNE plots for combined analysis from Figure 3 separated by sample
Shown are tSNE plots from the analysis shown in figure 3 separated by sample type: endogenous microglia (**a**), MLCs (**b**), treatment naïve microglia (**c**), astrocytes (**d**), neurons (**e**) and PBMCs (**f**).

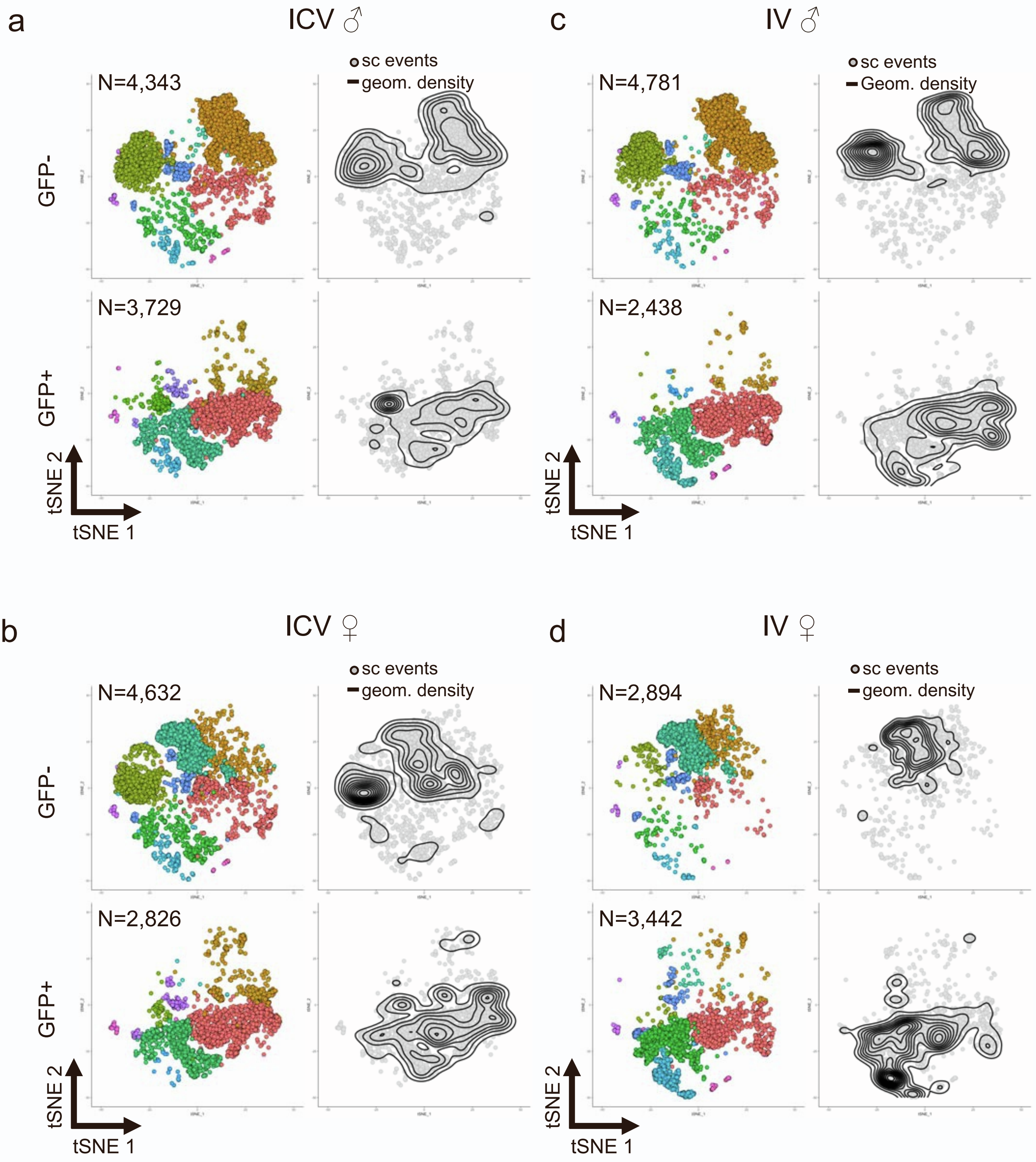

Supplemental Figure 7. tSNE plots for combined analysis from Figure 4 separated by sample type
Shown are tSNE plots from the analysis shown in figure 4 separated by animal. The GFP+ MLC and GFP- microglia samples are shown for the male (**a**) and female (**b**) dosed via ICV and for the male (**c**) and female (**d**) dosed via IV.

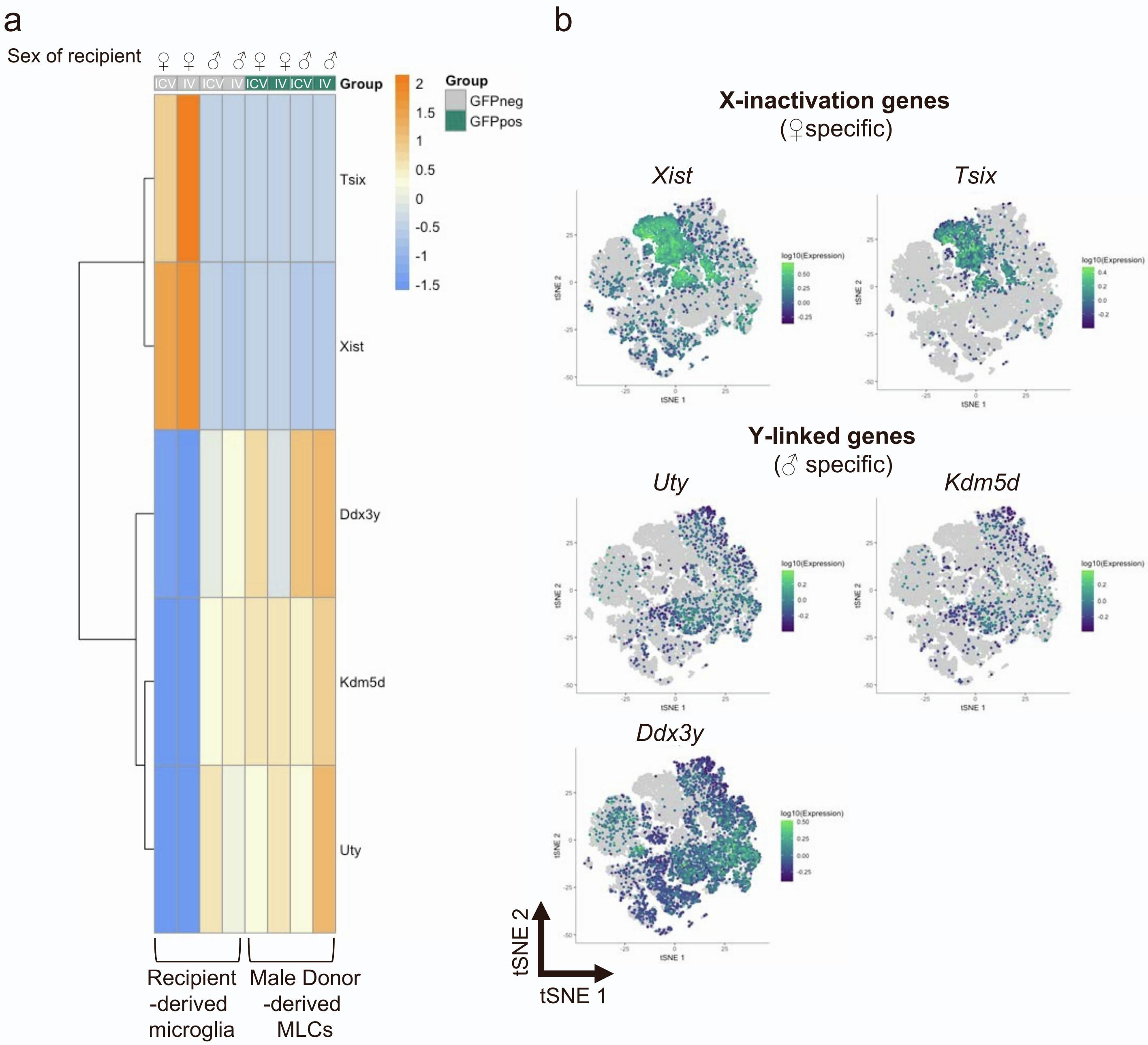

Supplemental Figure 8. Expression of sex-chromosome genes in MLCs and microglia.
a Heat maps of normalized expression for two X-linked genes associated with X-inactivation (*Xist*, *Tsix)* and three Y-linked genes (*Ddx3y*, *Uty*, *Kdm5d)* separated by sample type. **b** tSNE plot colored for expression of the X-linked and Y-linked genes.

a

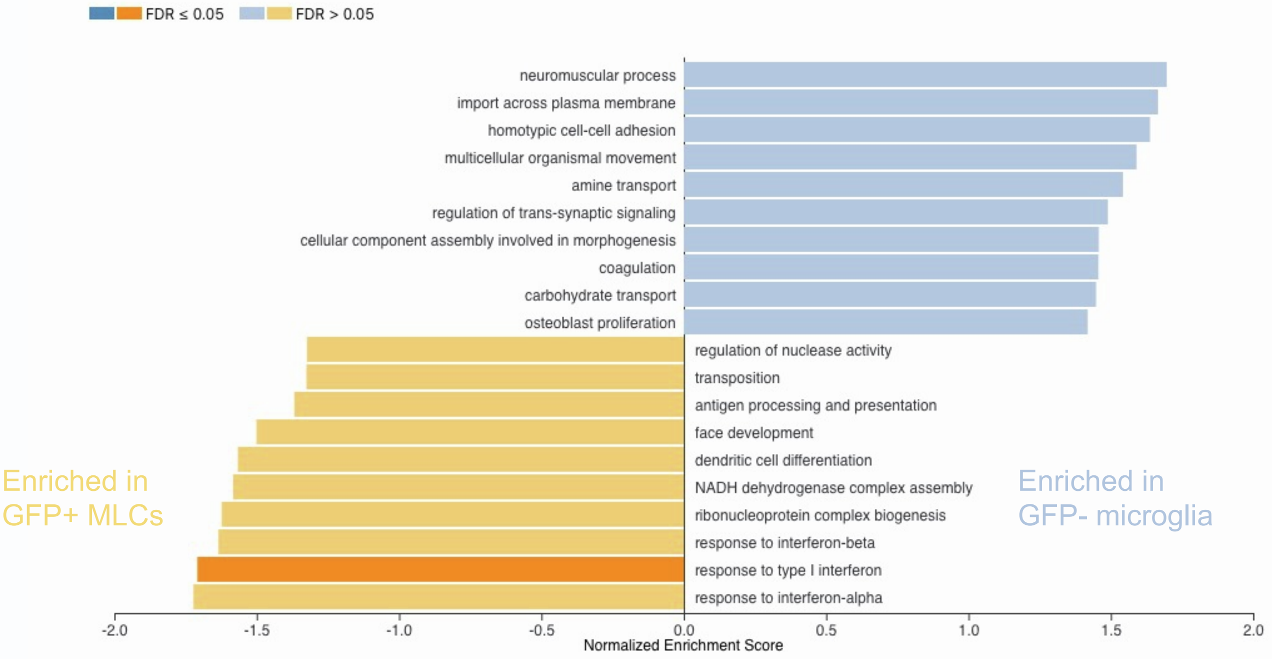

b

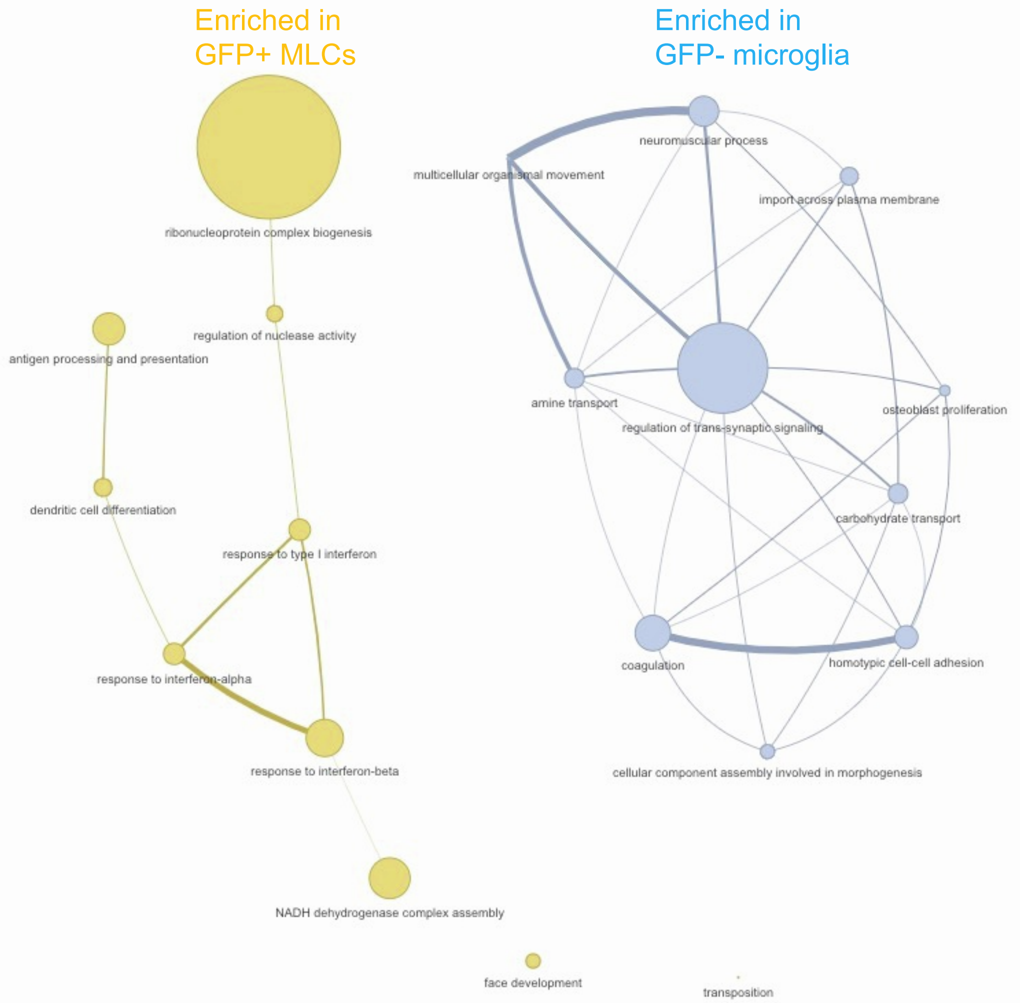

Supplemental Figure 9. Gene Ontology analysis comparing MLCs to microglia. a Top ten gene ontology terms associated with genes enriched in MLCS vs. endogenous microglia. GSEAPreranked analysis via webgestalt was used to generate GO term list. **b** Network-based enrichment map from GO terms is constructed by a R script using visNetwork R package.

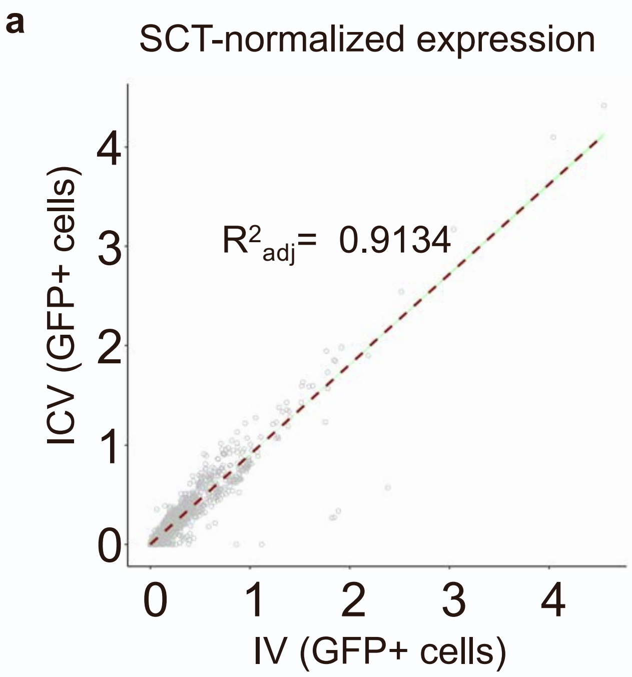

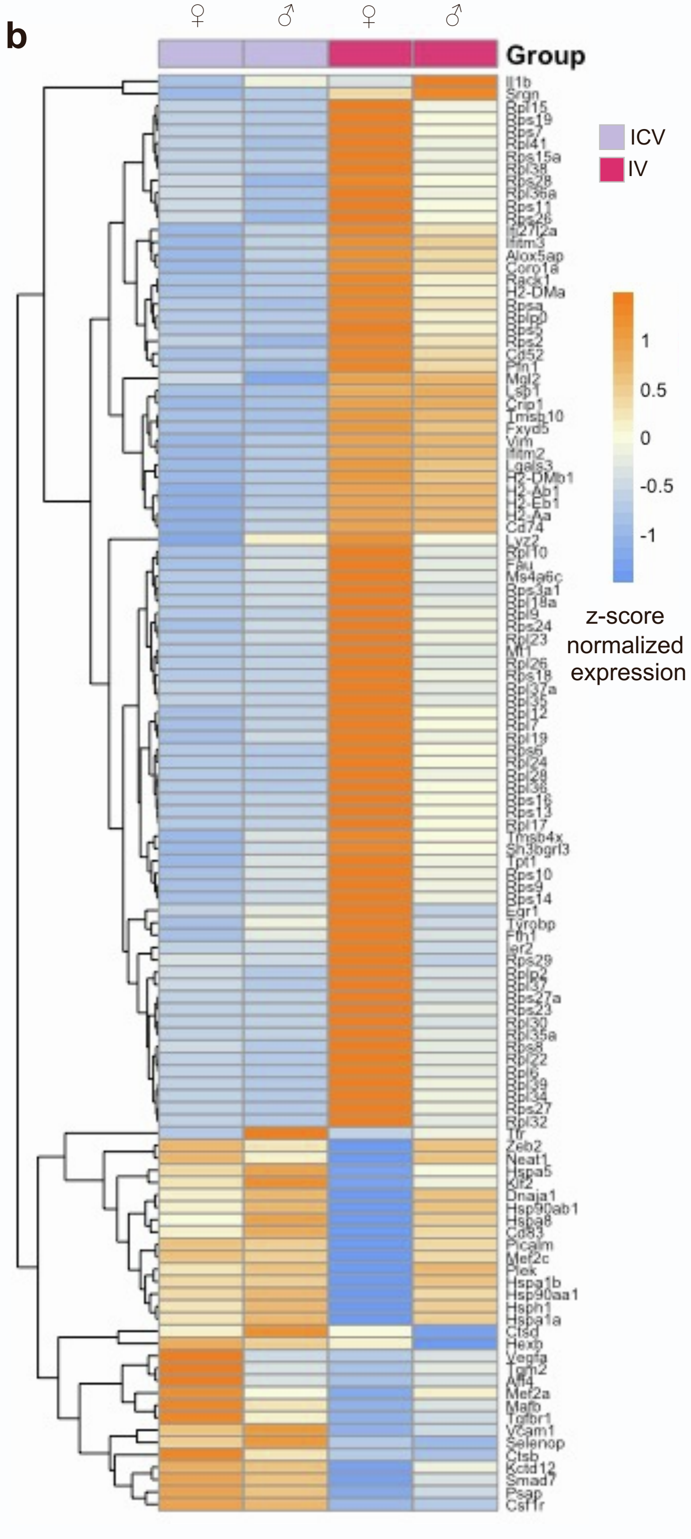

Supplementary Figure 10. Comparison of IV and ICV-derived MLCs. a Correlation analysis of MLCs isolated from IV and ICV dosed animals based on normalized single cell gene expression data. **b** Unsupervised clustering based on the global expression profile of each sample type (top). Heatmap showing top differentially expressed genes for each sample type.

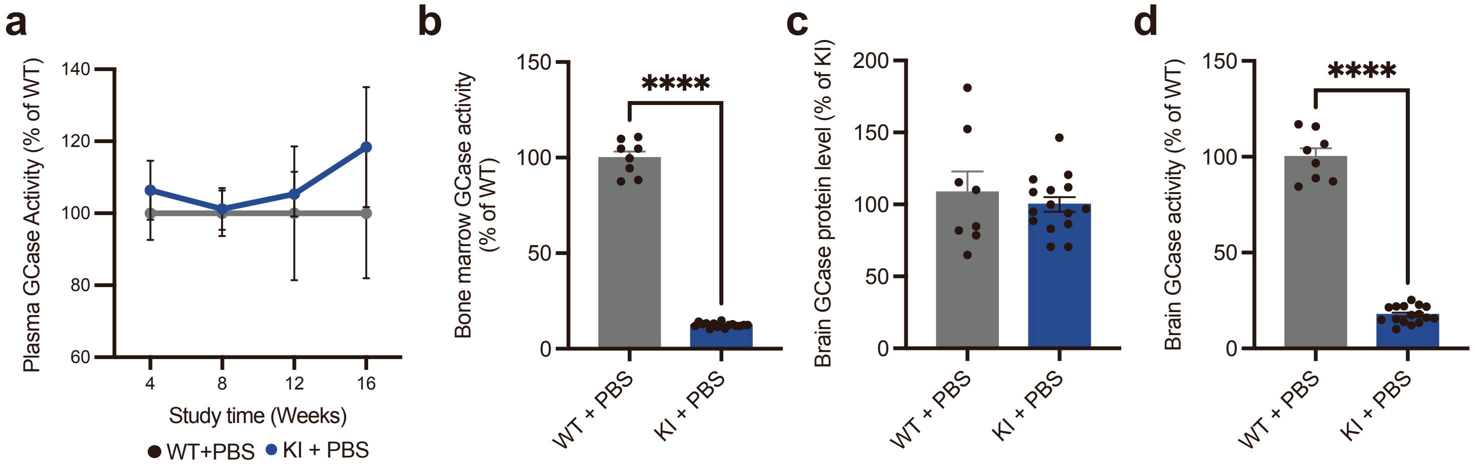

Supplementary Figure 11. Baseline characterization of homozygous Gba D409V knock-in mouse model. **a** GCase activity was measured from plasma at four week intervals through the course of the study and normalized to wild-type levels. Lack of difference between wild-type and knock-in animals is likely due to low endogenous activity and background noise. **b** GCase activity (normalized to wild-type animals) at the terminal timepoint of 16-weeks in the bone marrow. **c-d** GCase protein levels (normalized to knock-in animals) and activity (normalized to wild-type animals) were measured in the brain at the terminal timepoint of 16-weeks. T-test was used for statistical analysis. **, p<0.01. Bars represent means ± SEM.

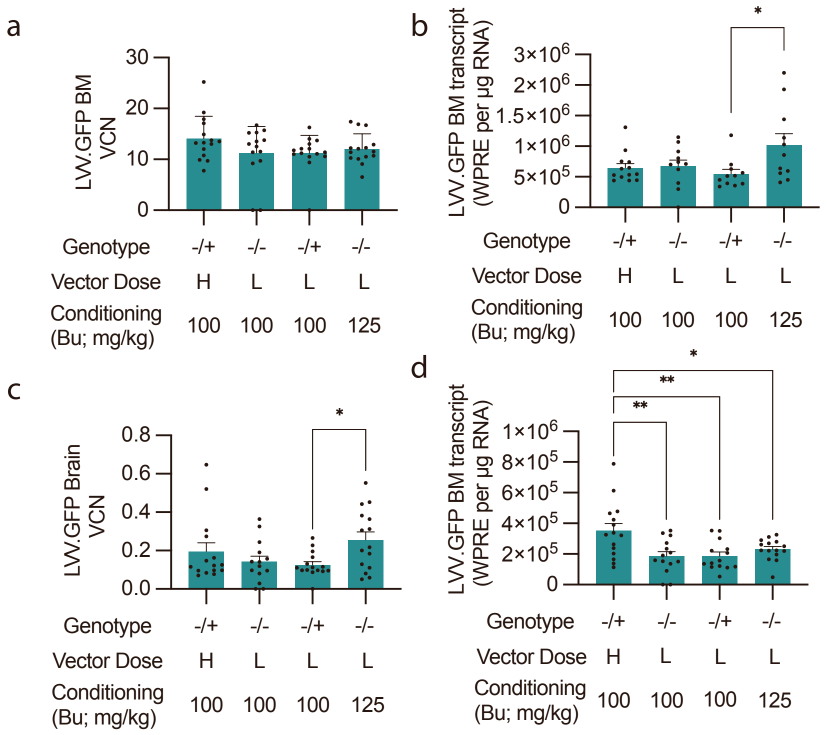

Supplementary Figure 12. VCN and WPRE quantification for LVV.GFP-treated animals at 16 weeks post-transplantation a Bone marrow VCN **b** Bone marrow WPRE **c** Brain VCN **d** Brain WPRE**.** All error bars are SEM. Tukey's multiple post-hoc comparison test was conducted for all statistical measures shown, with only significant differences indicated.

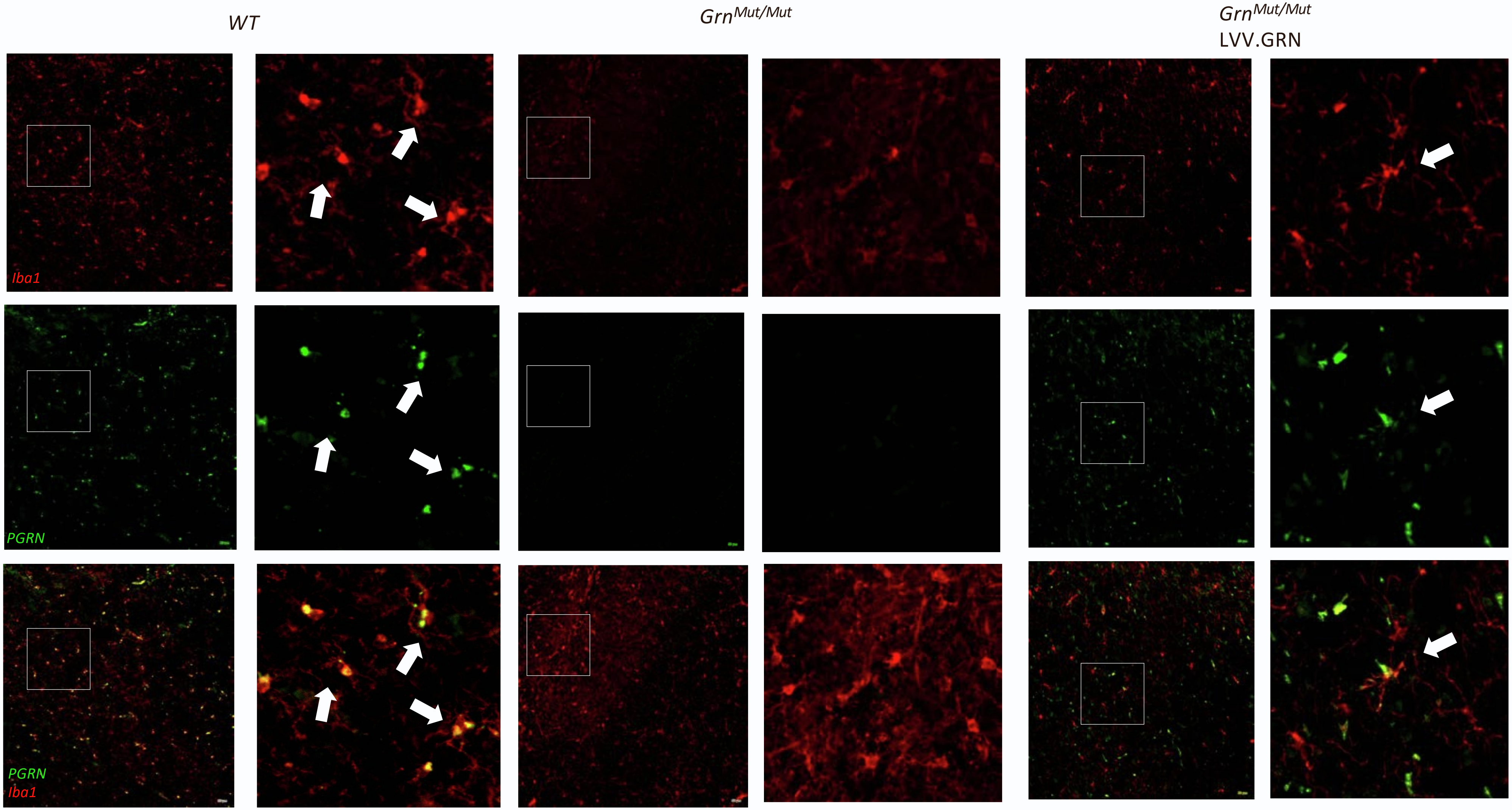

Supplementary Figure 13. Representative images of progranulin and Iba1 staining in WT, *Grn* mutant mice, and treated *Grn* mutant mice. Lack of staining in naïve *Grn* mutant animals demonstrates the specificity of the progranulin antibody. Staining is restored in *Grn* mutant mice after treatment. White boxes on the left image reflect the magnified image on the right. White arrows signify positive progranulin staining.
